# Supplementary material for: Rapid characterization of spike variants via mammalian cell surface display
Source: Mol Cell. 2021 Dec 16;81(24):5099–5111.e8. doi: 10.1016/j.molcel.2021.11.024 (PMC8675084; doi:10.1016/j.molcel.2021.11.024)
Supplement: Document S1. Figures S1–S7, Tables S1–S3, and Methods S1 [file mmc1.pdf]

Molecular Cell, Volume *81*

Supplemental information

Rapid characterization of spike variants

via mammalian cell surface display

Kamyab Javanmardi, Chia-Wei Chou, Cynthia I. Terrace, Ankur Annapareddy, Tamer S. Kaoud, Qingqing Guo, Josh Lutgens, Hayley Zorkic, Andrew P. Horton, Elizabeth C. Gardner, Giaochau Nguyen, Daniel R. Boutz, Jule Goike, William N. Voss, Hung-Che Kuo, Kevin N. Dalby, Jimmy D. Gollihar, and Ilya J. Finkelstein

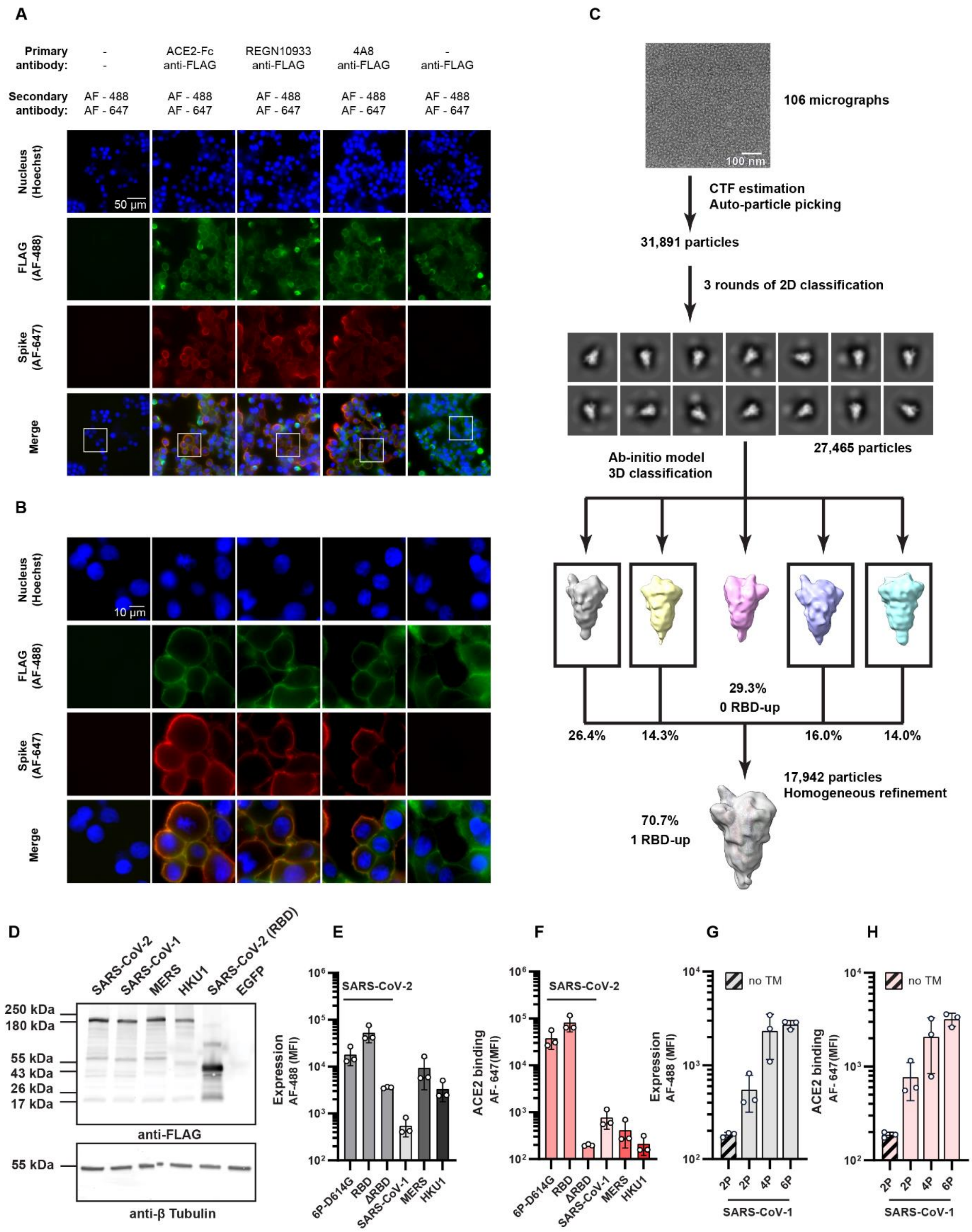
A
Primary antibody:
-
-
ACE2-Fc
anti-FLAG
REGN10933
anti-FLAG
4A8
anti-FLAG
-
anti-FLAG
Secondary antibody:
AF - 488
AF - 647
Nucleus (Hoechst)
FLAG (AF-488)
Spike (AF-647)
Merge
50 µm
B
10 µm
C
106 micrographs
100 nm
CTF estimation
Auto-particle picking
31,891 particles
3 rounds of 2D classification
Ab-initio model
3D classification
27,465 particles
29.3%
0 RBD-up
26.4%
14.3%
16.0%
14.0%
17,942 particles
Homogeneous refinement
70.7%
1 RBD-up
D
SARS-CoV-2
SARS-CoV-1
MERS
HKU1
SARS-CoV-2 (RBD)
EGFP
250 kDa
180 kDa
55 kDa
43 kDa
26 kDa
17 kDa
anti-FLAG
55 kDa
anti-β Tubulin
E
SARS-CoV-2
Expression AF-488 (MFI)
6P-D614G
RBD
ΔRBD
SARS-CoV-1
MERS
HKU1
F
SARS-CoV-2
ACE2 binding AF- 647(MFI)
G
no TM
Expression AF-488 (MFI)
2P
2P
4P
6P
SARS-CoV-1
H
no TM
ACE2 binding AF- 647(MFI)
SARS-CoV-1

Figure S1. Characterization of surface displayed SARS-CoV-2 spikes and related homologs, Related to Figure 1

(A) Fluorescent immunostaining of HEK293T cells transiently expressing SARS-CoV-2 spike (6P-D614G). Cells were immunostained 48 hours post transfection with the spike display construct. Full field view of HEK293Ts at 60X magnification. Top row indicates how cells were immunostained; the first column is a negative control. Last column shows no AF-647 cross-reactivity with anti-FLAG antibody.
(B) Higher magnification images from the white rectangular frames in (A). AF = Alexa Fluor.
(C) Schematic of pre-processing, classification, and refinement procedure to generate the map from 106 micrographs (STAR methods). Boxed maps indicate the maps showing 1-RBD up EM density in alignment with HexaPro (S-6P) 1-RBD up structure (6XKL) (Wrapp et al., 2020).
(D) Western blot of spike displayed proteins and RBD from HEK293T cells. Western blots were stained with anti-FLAG antibodies. EGFP is included as a negative control for anti-FLAG, and anti-β tubulin used as a loading control.
(E-F) Raw expression (E) and ACE2 binding (F) signals measured via spike display and flow cytometry. The ΔRBD construct is a negative control for background ACE2 binding.
(G-H) Raw expression (G) and ACE2 binding (H) signals for pre-fusion stabilized SARS-CoV-1 using prolines. 2P = F799P + A874P. 4P = 2P + A881P + S924P. 6P = 4P + K968P + V969P. no TM = SARS-CoV-1 (2P) with no transmembrane domain, used as a negative control for background expression and ACE2 binding. Circles represent biological replicates. Error bars = S.D.

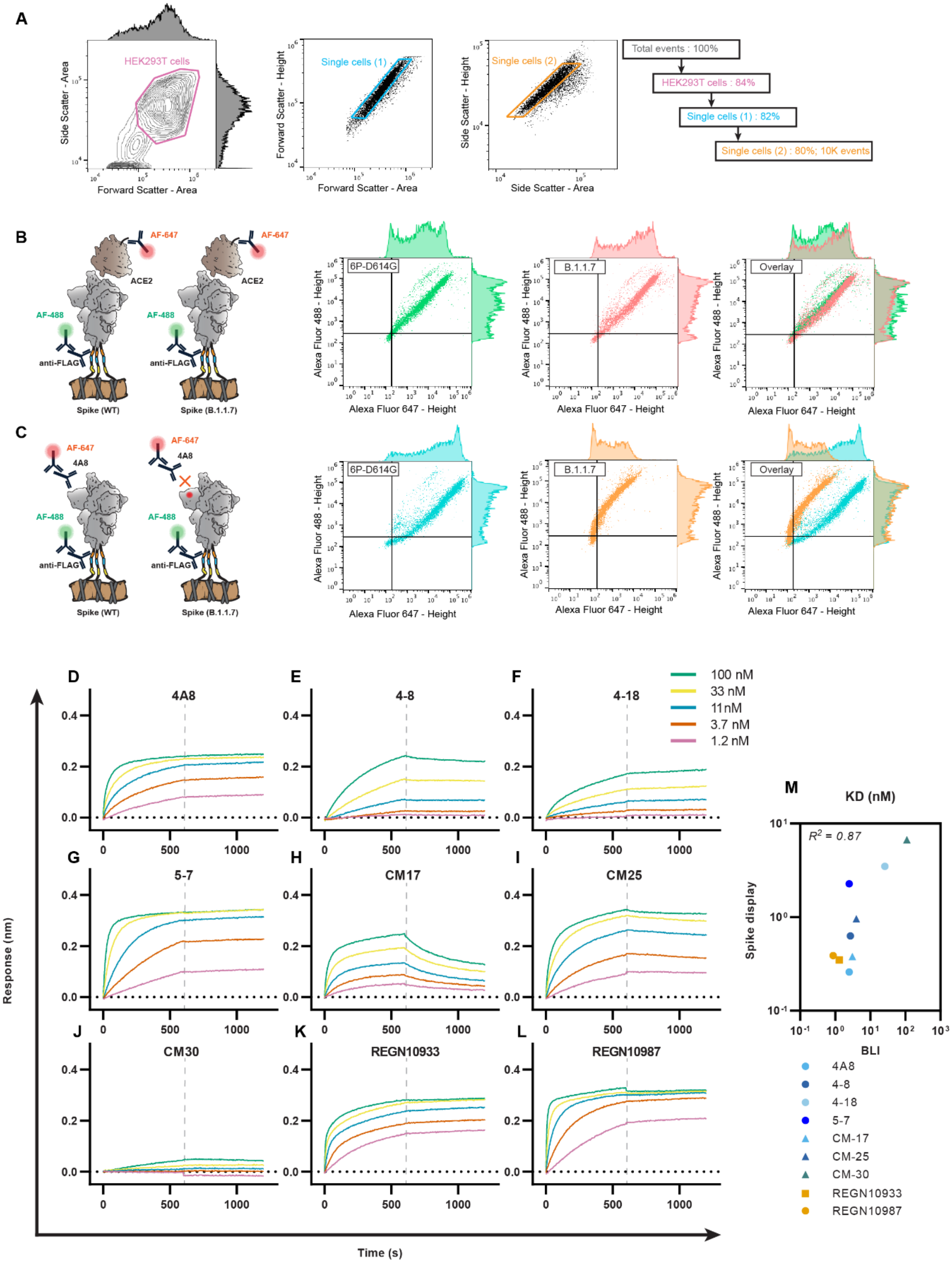
A
HEK293T cells
Side Scatter - Area
Forward Scatter - Area
Single cells (1)
Forward Scatter - Height
Forward Scatter - Area
Single cells (2)
Side Scatter - Height
Side Scatter - Area
Total events : 100%
HEK293T cells : 84%
Single cells (1) : 82%
Single cells (2) : 80%; 10K events
B
AF-647
ACE2
AF-488
anti-FLAG
Spike (WT)
Spike (B.1.1.7)
6P-D614G
B.1.1.7
Overlay
Alexa Fluor 488 - Height
Alexa Fluor 647 - Height
C
AF-647
4A8
AF-488
anti-FLAG
Spike (WT)
Spike (B.1.1.7)
6P-D614G
B.1.1.7
Overlay
Alexa Fluor 488 - Height
Alexa Fluor 647 - Height
D
4A8
E
4-8
F
4-18
100 nM
33 nM
11nM
3.7 nM
1.2 nM
G
5-7
H
CM17
I
CM25
J
CM30
K
REGN10933
L
REGN10987
Response (nm)
Time (s)
M
KD (nM)
R^2 = 0.87
Spike display
BLI
4A8
4-8
4-18
5-7
CM-17
CM-25
CM-30
REGN10933
REGN10987

Figure S2. Flow cytometry data processing and biolayer interferometry (BLI) of nAb binding to spike (6P-D614G), Related to Figure 1

(A) Representative gating used to select for single HEK293T cells expressing surface displayed spikes. HEK293T cells identified with side scatter-area (SSC-A) vs forward scatter-area (FSC-A) gating followed by doublet discrimination using forward scatter-height (FSH) vs forward scatter-area (FSC-A) and side scatter-height (SSC-H) vs side scatter-area (SSC-A) gates. Representative cell% retained from each gating step shown on the right.
(B) Graphical representation of the antibodies used (left) and the resulting flow cytometry data measuring ACE2 binding of 6P-D614G and B.1.1.7 spike variants (right). Single cells were analyzed using two fluorescent channels, Alexa Fluor-488 and Alexa Fluor-647 for measuring spike expression and antigenicity, respectively.
(C) Graphical representation (left) and flow cytometry data measuring 4A8 antibody binding of 6P-D614G and B.1.1.7 spike variants (right).
(D-L) Histograms representing signal distributions for Alexa Fluor-488 and Alexa Fluor-647 are included for (B) and (C). On- and off-rate curves for antibodies (D) 4A8, (E) 4-8, (F) 4-18, (G) 5-7, (H) CM17, (I) CM25, (J) CM30, (K) REGN10933, and (L) REGN10987.
(M) Correlation of the binding affinities measured by spike display and BLI.

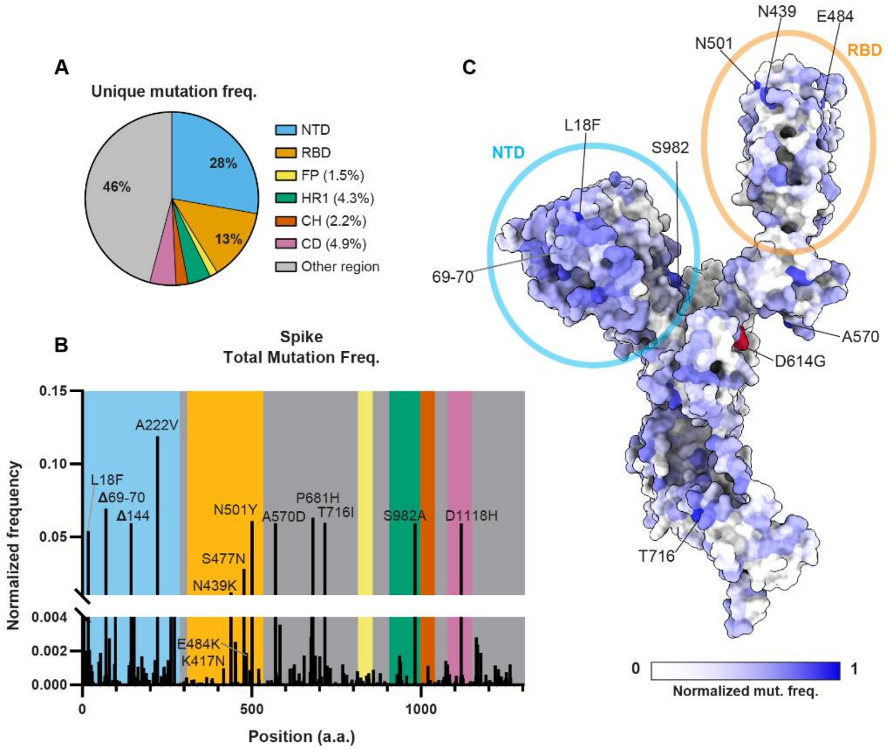

Figure S3. Summary of SARS-CoV-2 spike mutation frequencies, Related to Figure 2

(A) The distribution of spike mutations across key protein domains, as reported in the GISAID (accessed on 24/Feb/2021) (Elbe and Buckland-Merrett, 2017; Shu and McCauley, 2017).
(B) Normalized mutation frequencies, excluding the globally dominant D614G substitution. Spike domains are colored on the graph as in (A) and Figure 2A.
(C) A spike ectodomain monomer colored by mutation frequency (PDB: 7DDN (Zhang et al., 2021)). White: invariant position (no mutations). A few key residues are labeled. Position 614, labeled in red, is excluded from the frequency calculations because it is now globally dominant (Korber et al., 2020).

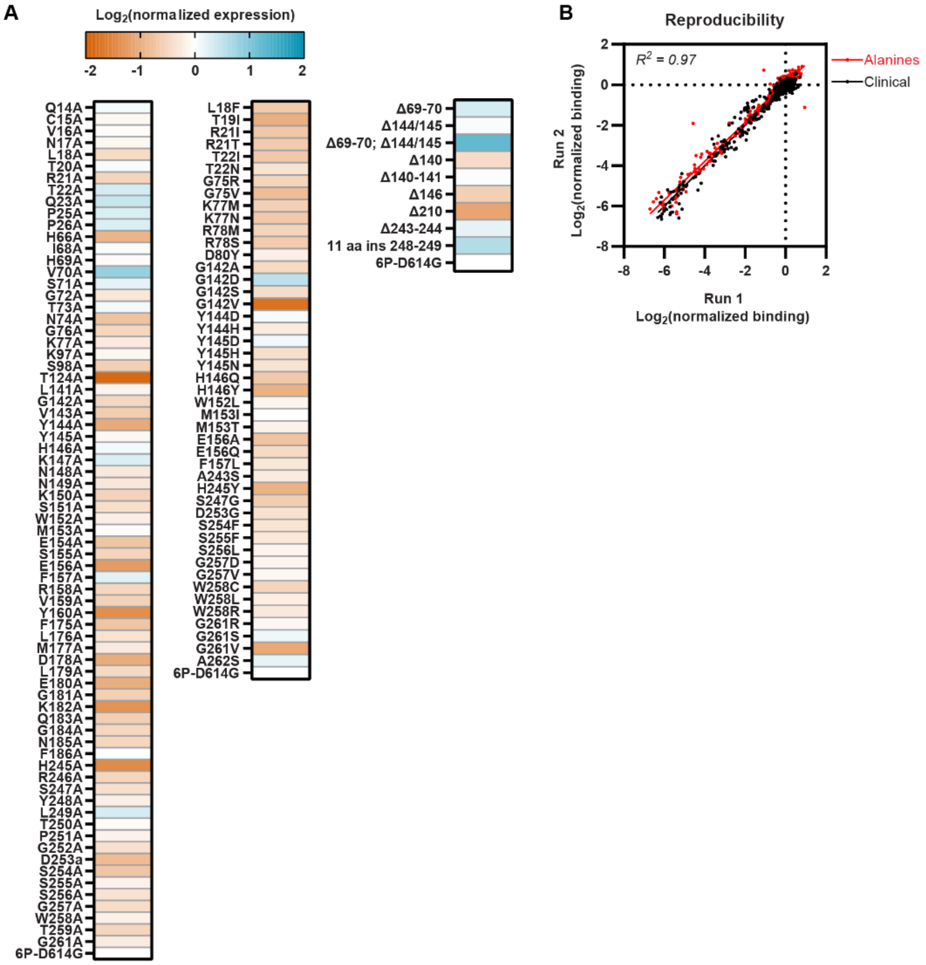

Figure S4. Spike expression measurements and assay reproducibility, Related to Figures 2 and 3

(A) Normalized spike expression for alanine and clinical variants in Figs. 2C, 3A, and 3E.
(B) Comparison of the nAb binding data across two biological replicates and all spike variants indicates excellent reproducibility. Red = alanine scan; Black = all clinical variants. Pearson correlation was identical for both datasets.

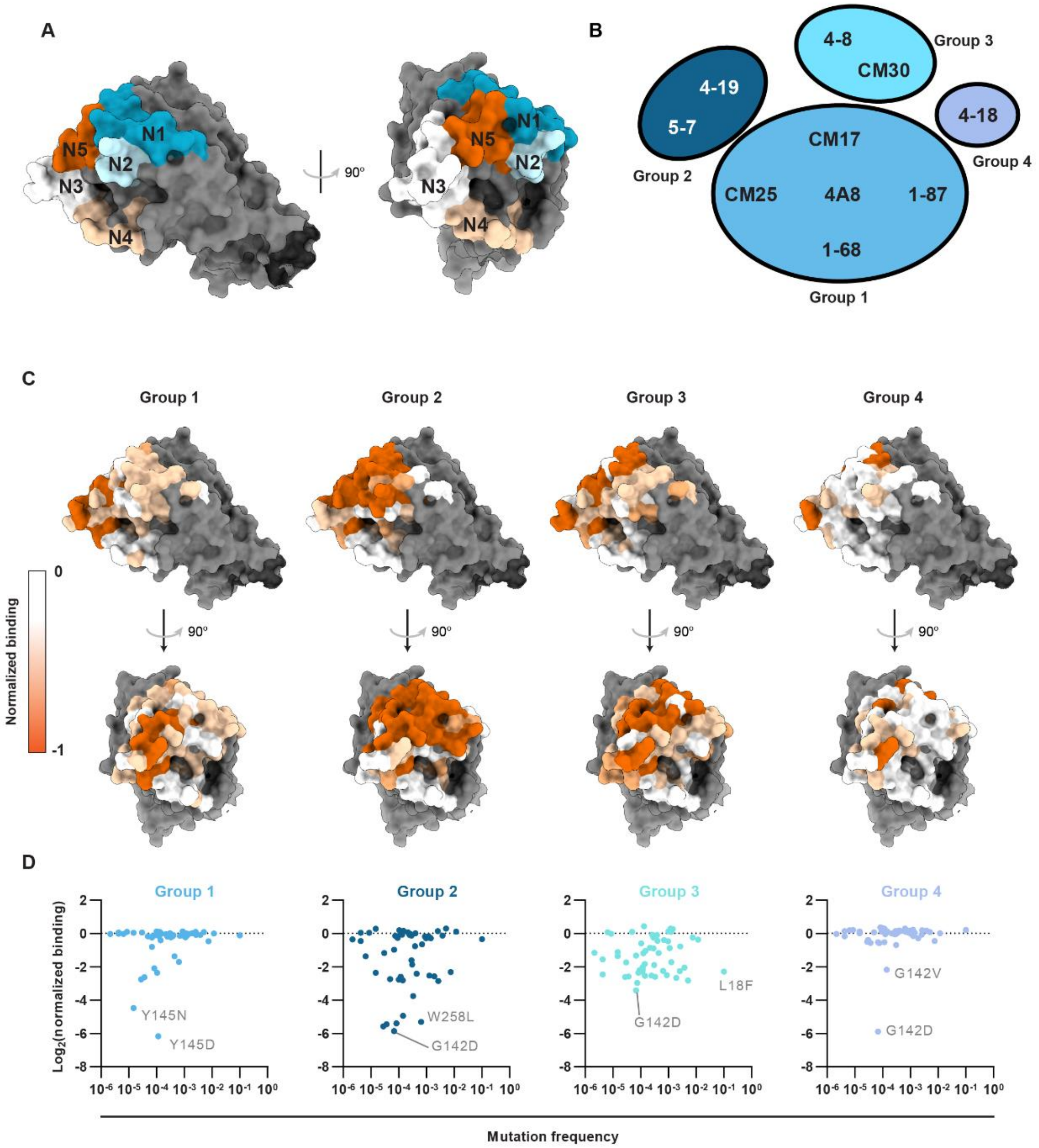
A
N1
N5
N2
N3
N4
90°
B
4-19
5-7
Group 2
4-8
CM30
Group 3
4-18
Group 4
CM17
CM25
4A8
1-87
1-68
Group 1
C
Group 1
Group 2
Group 3
Group 4
Normalized binding
0
-1
90°
D
Group 1
Group 2
Group 3
Group 4
Log2(normalized binding)
Y145N
Y145D
W258L
G142D
L18F
G142D
G142V
G142D
Mutation frequency

Figure S5. Antibody classification and epitope mapping, Related to Figure 3

(A) NTD loops N1 (residues 14-26), N2 (residues 67-79), N3 (residues 141-156), N4 (residues 177-186), and N5 (residues 246-260) uniquely colored and labeled on NTD structure (PDB: 7DDN (Zhang et al., 2021)).
(B) NTD binding nAbs grouped according to Pearson correlation matrix ($r > 0.5$) from Figure 3C.
(C) Normalized binding data from Figures 2C and 3A were averaged for all nAbs in each group and normalized to a -1(loss of binding) to 0 (no change in binding) scale and superimposed on the NTD (PDB: 7DDN (Zhang et al., 2021)). Gray: unmutated positions. Epitope maps highlight distinct binding patterns at the NTD supersite.
(D) Comparison of mutation frequency as a function of NTD nAb escape for each group.

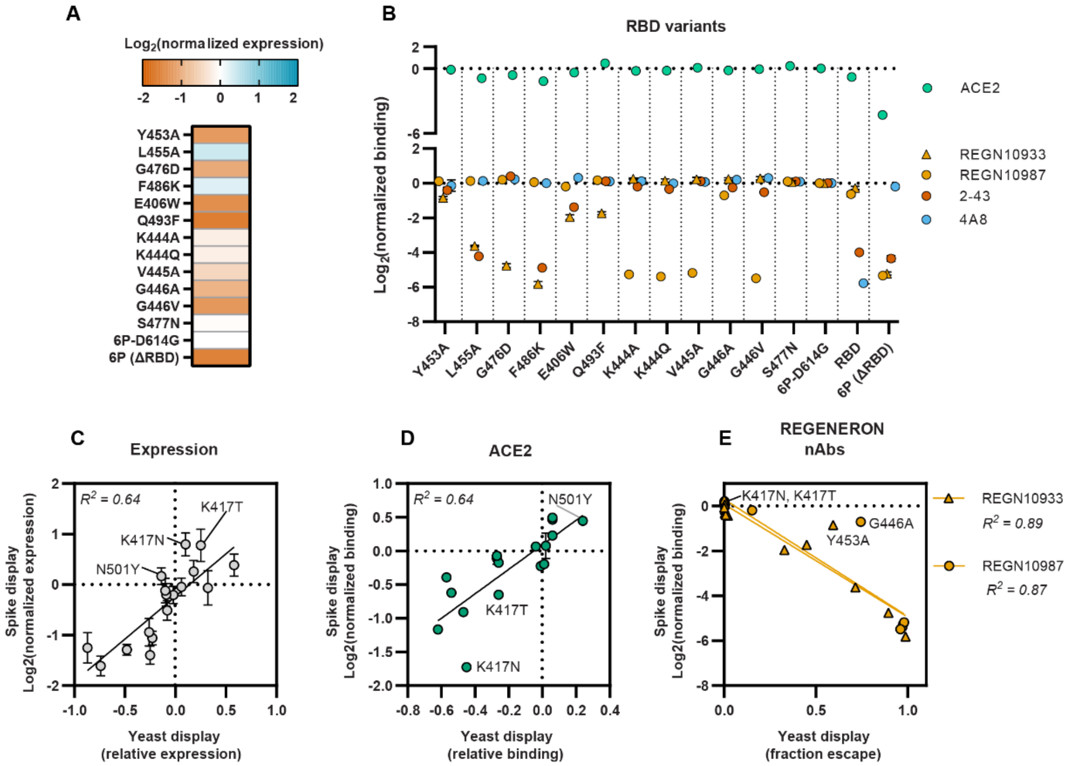

Figure S6. Analysis of RBD mutations and comparison to yeast RBD display, Related to Figure 3

(A-B) Expression (A) and binding (B) data for RBD variants that escape REGENERON nAbs REGN10933 and REGN10987. We also included the high prevalence S477N clinical mutation. Data in (A) and (C) were measured using spike display. Antibodies (2-43 and 4A8) and ACE2 were also screened. (n = 3 biological replicates; error bars = S.D.).

(C-E) Spike display correlates with yeast display of the isolated RBD (Starr et al., 2021, 2020) for (C) expression, (D) ACE2 binding, and (E) REGNERON nAb binding. Pearson correlations are indicated in each panel.

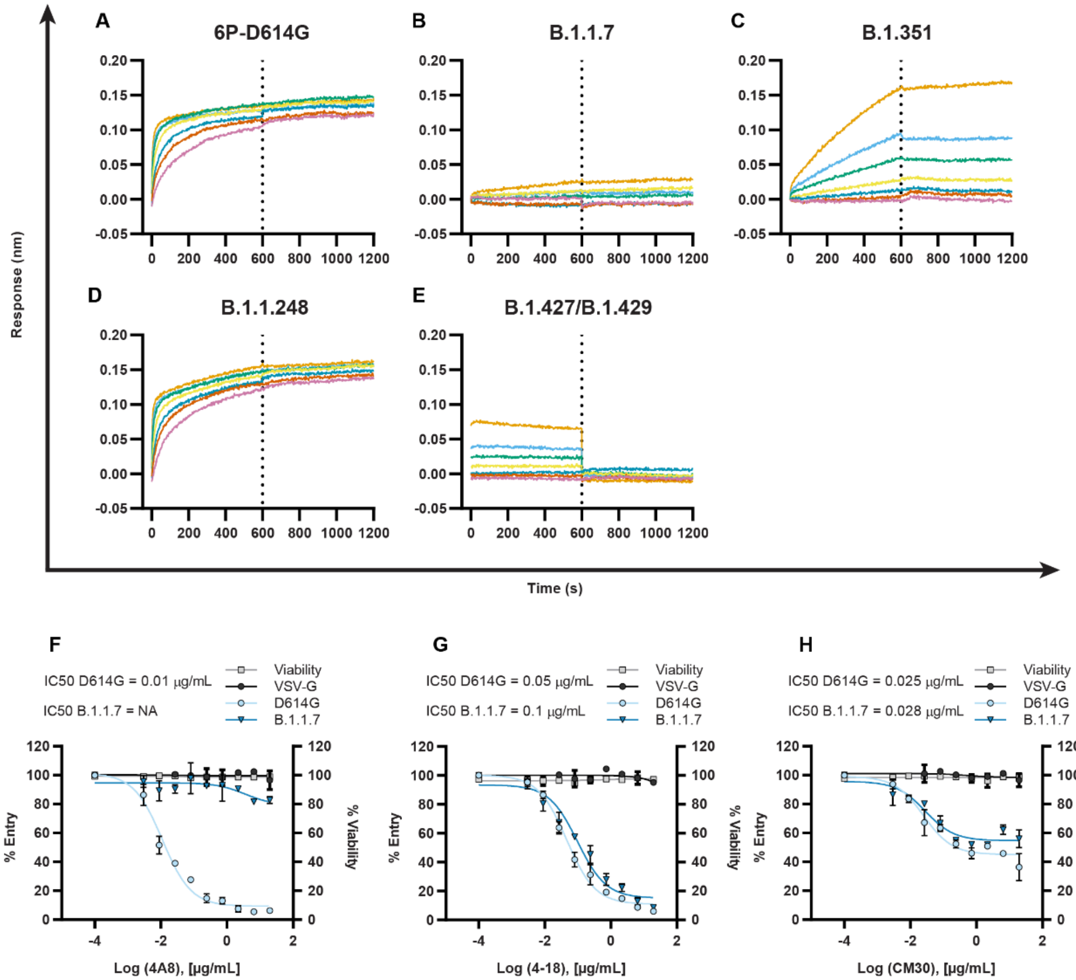

Figure S7. Biolayer interferometry of VOC spikes cleaved from cell surfaces and pseudovirus neutralization assays show nAb escape, Related to Figures 4 and 5

(A-E) BLI spectrogram showing the spike capture strategy. Anti-mouse Fc (AMC) tips are loaded with mouse anti-FLAG antibodies. The tips then capture SARS-CoV-2 spikes cleaved from cell surfaces with 3C protease (STAR methods). BLI tips are further incubated in antibody (4A8) solutions of varying concentrations to observe "on" and "off" rates. (A-E) BLI on and off rate curves for 4A8 against 6P-D614G (A), B.1.1.7 (B), B.1.351 (C), B.1.1.248 (D), and B.1.427/B.1.429 (E) spikes. BLI experimental design matches that shown in (A). On/off rate transition for (A-E) indicated by vertical dotted lines.

(F-H) SARS-CoV-2 pseudovirus neutralization curves using 4A8 (F), 4-18 (G), and CM30 (H) nAbs. Results compare D614G (light blue) and B.1.1.7 (dark blue) pseudovirus variants (STAR methods). VSV-G (black) included as a negative control for neutralization. Cell viability data included in gray. Dots and error bars indicate mean and S.D of three replicates.

Table S1. Comparison of antibody binding affinities using different platforms, Related to Figure 1

Antibody	Specificity	Spike Display K_D (nM)	BLI K_D (nM)°	Literature K_D (nM)
4A8 (Chi et al., 2020)	NTD	0.26 ± 0.04	2.5 ± 0.06	0.996 ± 0.045[*]
4-8 (Liu et al., 2020)	NTD	0.63 ± 0.04	2.7 ± 0.7	0.150 ± 0.037[#,§]
4-18 (Liu et al., 2020)	NTD	3.5 ± 0.2	26 ± 4	0.216 ± 0.040[#,§]
5-7 (Liu et al., 2020)	NTD	2.3 ± 0.1	2.5 ± 0.3	0.0950 ± 0.011[#,§]
CM17 (Voss et al., 2021)	NTD	0.38 ± 0.04	3.1 ± 1	9.34 ± 0.16[#]
CM25 (Voss et al., 2021)	NTD	0.96 ± 0.03	4.0 ± 0.2	9.44 ± 0.055[#]
CM30 (Voss et al., 2021)	NTD	6.7 ± 0.6	110 ± 30	0.841 ± 0.84[#]
2-43 (Liu et al., 2020)	S1	0.22 ± 0.2	N/A	1.66 ± 1.5[&]
REGN10933 (Hansen et al., 2020)	RBD	0.35 ± 0.05	1.3 ± 0.2	0.0417[@]
REGN10987 (Hansen et al., 2020)	RBD	0.39 ± 0.02	0.87 ± 0.07	0.0428[@]

° Measured in this study using the full IgG.

* Measured via bio-layer interferometry (BLI).

Measured via enzyme-linked immunosorbent assays (ELISAs)

@ Measured via surface plasmon resonance (SPR).

§ Digitized with WebPlotDigitizer and fit with a 4PL curve in GraphPad Prism 9. Error bar: 95% CI of the fit.

& Digitized with WebPlotDigitizer and fit with a 4PL curve in GraphPad Prism 9. Original data were measured via cell-surface competition binding assay. Error bar: 95% CI of the fit.

Table S2. Biolayer interferometry of 4A8 to spike variants of concern, Related to Figure 4 and Figure S7

Lineage	4A8 mAb<br>K_D (nM)
WT	2.3 ± 0.4
B.1.1.7	N/D
B.1.351	34 ± 3
B.1.1.248	2.0 ± 0.4
B.1.427/B.1.429	N/D

N/D: Not determined because 4A8 binding was below the detection limit.

Table S3. Synthetic DNA sequences for cloning, Related to STAR methods

Oligo ID	DNA sequence (5' to 3')	Purpose
Primers		
P1	TACCACGCGTGAATTCTCGAGG	PCR of pcDNA5 bb (Rev)
P2	CGGCCGCTCGAGTCTAGAG	PCR of pcDNA5 bb (Fwd)
P3	ATGTTCGTGTTCCTGGTGCTCC	PCR of SARS-CoV-2 Spike (Fwd)
P4	GCCCAGGAATGTGCTCAGC	PCR of SARS-CoV-2 Spike (Rev)
P5	tgaacgctgaCTGCAGGCAAGCTTGGCAC	PCR of MBP-TEV bb (Fwd)
P6	ggttttcagaCGAATTAGTCTGCGCGTCTTTC	PCR of MBP-TEV bb (Rev)
P7	gactaattcgTCTGAAAACCTGTATTTCCAG	PCR LCB1 insert (Fwd)
P8	ttgcctgcagTCAGCGTTCAACTTCCTC	PCR LCB1 insert (Rev)
P9	acctgccaccTGTGGGCTACCTGCAACC	PCR of Spike Display bb for Part1 DO plasmid (Fwd)
P10	acctgccaccGTCACCAGTGGAACCTGG	PCR of Spike Display bb for Part1 DO plasmid (Rev)
P11	cactggtgacGGTGGCAGGTGGAAAGTG	PCR of sfGFP DO insert for Part1 DO plasmid (Fwd)
P12	gtagcccacaGGTGGCAGGTGTATAAACG	PCR of sfGFP DO insert for Part1 DO plasmid (Rev)
P13	acctgccaccGCCACCGTCTGCGGTCCT	PCR of Spike Display bb for Part2 DO plasmid (Fwd)
P14	acctgccaccCACATAGTAGGCTGCAGCTCCG	PCR of Spike Display bb for Part2 DO plasmid (Rev)
P15	ctactatgtgGGTGGCAGGTGGAAAGTG	PCR of sfGFP DO insert for Part2 DO plasmid (Fwd)
P16	agacggtggcGGTGGCAGGTGTATAAACG	PCR of sfGFP DO insert for Part2 DO plasmid (Rev)
P17	GCGTTTATACACCTGCCACC GATCTACAAGACCCCACCTATCAAGGAC	PCR of Spike Display bb for Part3 DO plasmid (Fwd)
P18	TTCACTTTCCACCTGCCACC TGGCGGGGGCGTGGAGTAAC	PCR of Spike Display bb for Part3 DO plasmid (Rev)
P19	GTTACTCCACGCCCCCGCCA GGTGGCAGGTGGAAAGTGAA	PCR of sfGFP DO insert for Part3 DO plasmid (Fwd)
P20	GTCCTTGATAGGTGGGGTCTTGTAGAT CGGTGGCAGGTGTATAAACGC	PCR of sfGFP DO insert for Part3 DO plasmid (Rev)
P21	ACCTGCCACCCAGAGCGCGCCGCAC	PCR of Spike Display bb for Part4 DO plasmid (Fwd)
P22	ACCTGCCACCGATCTGCTTCACTTGGGCG	PCR of Spike Display bb for Part4 DO plasmid (Rev)
P23	GAAGCAGATCGGTGGCAGGTGGAAAGTG	PCR of sfGFP DO insert for Part4 DO plasmid (Fwd)
P24	GCGCGCTCTGGGTGGCAGGTGTATAAACG	PCR of sfGFP DO insert for Part4 DO plasmid (Rev)

P25	ACCTGCCACCGCAGGGATCCGGATACATCCC CG	PCR of Spike Display bb for Part5 DO plasmid (Fwd)
P26	ACCTGCCACCTCTGGGGGAAGCTCATCAGG	PCR of Spike Display bb for Part5 DO plasmid (Rev)
P27	TTCCCCCAGAGGTGGCAGGTGGAAAGTG	PCR of sfGFP DO insert for Part5 DO plasmid (Fwd)
P28	GGATCCCTGCGGTGGCAGGTGTATAAACG	PCR of sfGFP DO insert for Part5 DO plasmid (Rev)
P29	acctgccaccGCAGGGATCCGGATACATC	PCR of Spike Display bb for Part1-5 DO plasmid (Fwd)
P30	acctgccaccGTCACCAGTGGAACCTGG	PCR of Spike Display bb for Part1-5 DO plasmid (Rev)
P31	cactggtgacGGTGGCAGGTGGAAAGTG	PCR of sfGFP DO insert for Part1-5 DO plasmid (Fwd)
P32	ggatccctgcGGTGGCAGGTGTATAAACG	PCR of sfGFP DO insert for Part1-5 DO plasmid (Rev)
gBlocks		
G1	CCTCGAGAATTCACGCGTGGTACCTCTAGAG TCGACCCGGGATGGAGACAGACACACTCCTG CTATGGGTACTGCTGCTCTGGGTTCCAGGTTC CACTGGTGACATGTTCGTGTTCCTGGTGCT	Ig-Kappa leader
G2	TGCTGAGCACATTCCTGGGCAGATCCGACTA CAAAGACCATGACGGTGATTATAAAGATCAT GACATCGATTACAAGGATGACGATGACAAG AGCGCCTGGTCCCACCCCCAGTTCGAGAAGG GATCCCTGGAGGTGCTGTTCCAGGGCCCAGG CGGCGGCGGTAGTGGAGGGGGCGGATCTGG CGGCTCAGCTGCTGTGGGCCAGGACACGCAG GAGGTCATCGTGGTGCCACACTCCTTGCCCTT TAAGGTGGTGGTGATCTCAGCCATCCTGGCC CTGGTGGTGCTCACCATCATCTCCCTTATCAT CCTCATCATGCTTTGGCAGAAGAAGCCACGT TAACGGCCGCTCGAGTCTAG	C-terminal linker
G3	ACCAACAAGGACCATAGCATATGTCCCATCA CCATCACCATCACCATCACTCTGAAAACCTG TATTTCCAGAGCGGCGGTGGCGACAAGGAAT GGATCCTGCAGAAAATTTATGAAATCATGCG CCTCCTGGACGAGCTGGGCCACGCTGAAGCG TCCATGCGTGTTTCCGACCTGATCTACGAATT CATGAAGAAAGGCGACGAACGCCTCCTGGA GGAAGCTGAACGCCTCCTGGAGGAAGTTGAA CGCTGACTGCAGGCAAGCTTGGC	LCB1

Methods S1. Protocols for vector assembly, transient expression in human cell culture, and analysis by flow cytometry, Related to STAR Methods

Version 1

Oct 12, 2021

Protocol 1A: Spike Display vector assembly

Protocol 1B: Transient expression of Spike Display in human cell culture

Protocol 1C: HEK292T immunostaining and flow cytometry

Protocol 1A: Spike Display vector assembly

All cloning vectors and parts used for Spike Display assemblies have been deposited on Addgene. See the table below for plasmid reference numbers. Addgene plasmids will be shipped as transformed bacteria in stab culture format. Upon receipt, stabs should be kept at 4 °C. Cells from the stab should be streaked out for single colonies on LB Agar plates with the appropriate antibiotic. Using a single colony, inoculate an overnight LB culture and perform plasmid DNA purifications. We recommend sequence verifying all plasmids upon receipt.

Addgene ID	Plasmid name
172721	Spike Display_Part-1 DO
172722	Spike Display_Part-2 DO
172723	Spike Display_Part-3 DO
172724	Spike Display_Part-4 DO
172725	Spike Display_Part-5 DO
172726	Spike Display_Part 1-5 DO
172727	Spike Display_Part 1a Spacer
172728	Spike Display_Part 1b Spacer
172729	Spike Display_Part-1 Spacer
172730	Spike Display_Part-2 Spacer
172731	Spike Display_Part-3 Spacer
172732	Spike Display_Part-4 Spacer
172733	Spike Display_Part-5 Spacer
172734	Spike Display_6P-D614G
172735	Spike Display_B.1.1.7
172736	Spike Display_B.1.351
172737	Spike Display_B.1.1.248
172738	Spike Display_B.1.427/B.1.429

The Spike Display platform features a modular Golden Gate cloning framework for the assembly of spike protein variants. The entirety of the spike protein coding region is divided into 5 parts with junctions strategically positioned at amino acids with low mutational frequencies, according to the GISAID (see below).

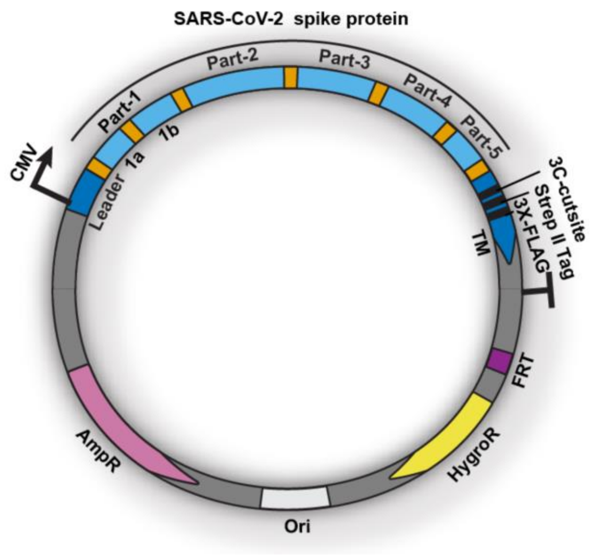

For each of the 5 parts, an entry vector was constructed by cloning in a superfolder GFP (sfGFP) bacterial expression cassette with flanking AarI cut sites and unique 4 nt overhangs matching the wild-type SARS-CoV-2 sequence of each part junction, using PCR and Hi-Fi DNA assembly (see below). A part 1-5 entry vector was made with the entire SARS-CoV-2 coding sequence replaced with the sfGFP cassette to enable multi-part assemblies of complex spike variants or entirely different spike proteins.

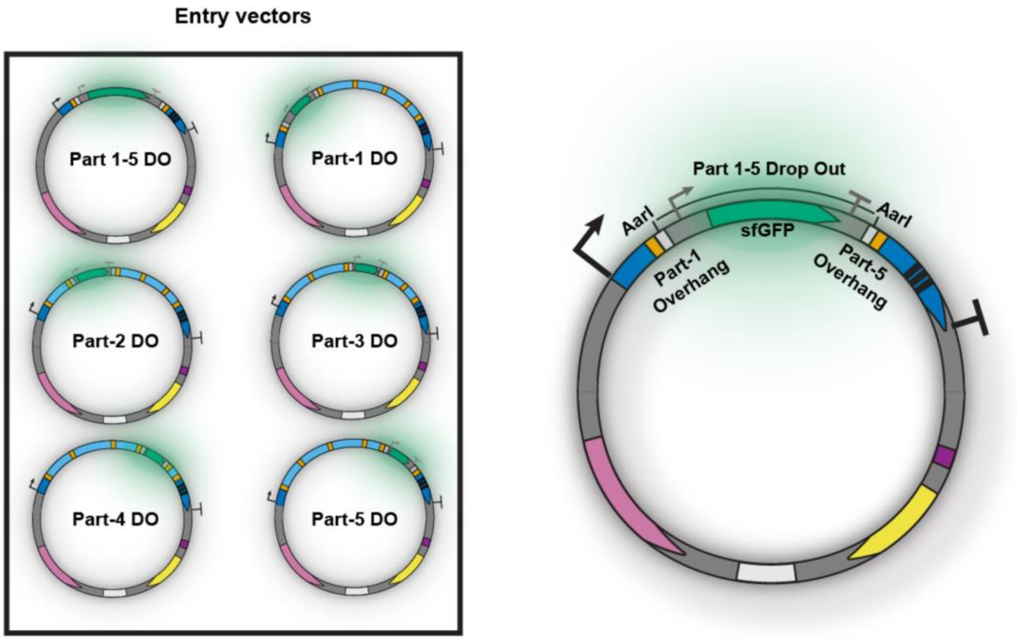

When assembling a new Spike Display vector with the desired mutations, first identify the amino acid (a.a.) position in the spike protein coding sequence. Use the table below to determine which

part your desired mutation(s) fall into. For example, the nonsynonymous mutation N501Y (N->Y mutation at position 501 in the SARS-CoV-2 spike protein coding sequence) is in Part-2.

Part	5' Overhang	3' Overhang	3' Overhang junction (a.a.)	Coding sequence positions (a.a.)
1a	TGAC	GCAA	136-137	1-135
1b	GCAA	TGTG	266-267	138-265
1	TGAC	TGTG	266-267	1-265
2	TGTG	GCCA	522-523	268-521
3	GCCA	GATC	787-788	524-786
4	GATC	CAGA	1054-1055	789-1053
5	CAGA	GCAG	1207-1208	1054-1206

Next, dsDNA gene blocks (synthetic oligos) need to be designed an ordered for each modified part. For these gene blocks to be compatible with the Golden Gate cloning framework used here, 5’ and 3’ overhangs containing AarI endonuclease recognition sequences need to be included (see below). During the Golden Gate assemblies AarI will remove its own recognition sequence and expose 4 nt overhangs on the 5’ and 3’ ends of each Part, which are complementary to the flanking Part or plasmid backbone overhangs.

Gene block designs

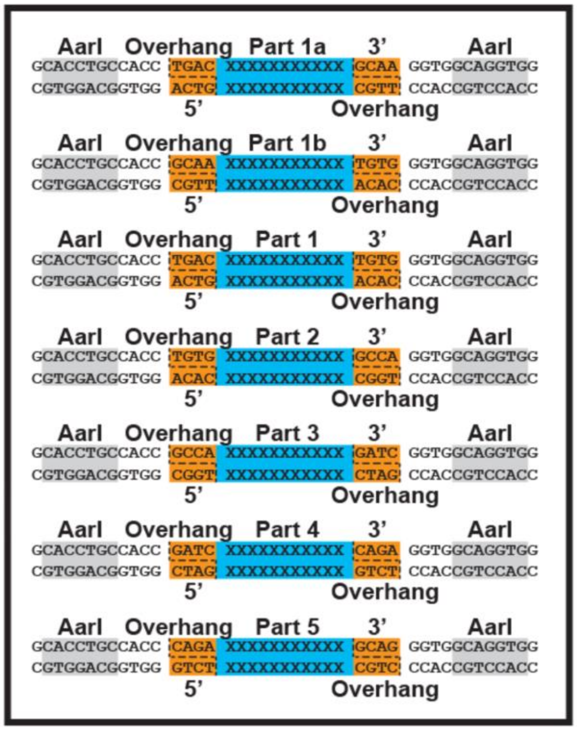

Complete Part sequences for the SARS-CoV-2 spike protein (D614G, ^{682}GSAS^{685}, F817P, A892P, A899P, A942P, K986P, V987P) with the appropriate overhangs are included in the table below. These sequences can be used as templates for ordering custom Parts (dsDNA oligos) with the desired mutations.

<u>Design tips</u>:

1. Consider codon optimization when creating nonsynonymous mutations. As this expression system is tailored for human cell cultures, human codon optimization should be used.
2. Ensure that desired substitutions, insertions, and deletions are in the appropriate reading frame.
3. Perform *in silico* assemblies with the designed parts and entry vectors for QC.
4. Remove any of the following internal cut sites generated during the design of gene block Parts: BsaI, BsmBI, AarI
5. If a desired mutation is located at a Part junction (4 nt overhangs), a larger gene fragment can be purchased which spans the junction. For example, a desired sequence change located at the 3' end of Part 3 can be designed by ordering a Part 3-4 gene fragment. Alternatively, traditional cloning strategies such as site-directed mutagenesis or Gibson assembly can be performed for these special cases.

Part name	Gene Block sequence (5'-3')
Part-1a	GCATCGTCTCATCGGCACCTGCCACCTGACATGTTCGTGTTCCTG<br>GTGCTCCTGCCTCTGGTGAGCAGCCAGTGCGTGAACCTGACCAC<br>CCGAACCCAGCTCCCACCAGCCTACACCAACAGCTTTACACGGG<br>GCGTGTACTACCCTGACAAGGTGTTCAGATCTAGCGTCCTGCAC<br>AGCACTCAGGACCTCTTCCTGCCGTTCTTCAGCAACGTGACATGG<br>TTCCACGCCATCCACGTGAGCGGCACAAACGGAACCAAGCGGTT<br>TGATAACCCCGTCCTGCCATTCAATGATGGAGTTTACTTCGCCAG<br>TACCGAGAAGAGTAACATCATCCGGGGCTGGATCTTCGGCACCA<br>CCCTGGATAGCAAAACACAGAGCCTCCTGATCGTGAACAATGCC<br>ACGAACGTCGTGATCAAGGTGTGCGAGTTCCAGTTTTGCAAGGT<br>GGCAGGTGGACCTGAGACGGCAT
Part-1b	GCATCGTCTCATCGGCACCTGCCACCGCAATGATCCTTTCCTGGG<br>TGTGTACTACCACAAGAACAACAAGAGCTGGATGGAAAGCGAG<br>TTCAGAGTCTACAGCAGCGCCAACAACTGCACATTCGAGTACGT<br>CTCTCAGCCTTTTCTGATGGACCTTGAGGGGAAACAAGGCAACT<br>TCAAGAACCTGAGAGAATTCGTGTTCAAGAACATCGACGGCTAC<br>TTCAAAATCTACTCCAAGCACACACCCATCAACCTGGTCCGGGA<br>CCTCCCTCAGGGCTTCAGCGCCCTGGAACCCCTGGTCGACCTGCC<br>CATAGGCATCAACATAACGCGGTTCCAAACCCTGCTGGCCCTGC<br>ATAGATCCTACCTGACTCCTGGCGACAGCAGCAGCGGATGGACC<br>GCCGGAGCTGCAGCCTACTATGTGGGTGGCAGGTGGACCTGAGA<br>CGGCAT
Part-1	GCATCGTCTCATCGGCACCTGCCACCTGACATGTTCGTGTTCCTG<br>GTGCTCCTGCCTCTGGTGAGCAGCCAGTGCGTGAACCTGACCAC<br>CCGAACCCAGCTCCCACCAGCCTACACCAACAGCTTTACACGGG<br>GCGTGTACTACCCTGACAAGGTGTTCAGATCTAGCGTCCTGCAC

	AGCACTCAGGACCTCTTCCTGCCGTTCTTCAGCAACGTGACATGG<br>TTCCACGCCATCCACGTGAGCGGCACAAACGGAACCAAGCGGTT<br>TGATAACCCCGTCCTGCCATTCAATGATGGAGTTTACTTCGCCAG<br>TACCGAGAAGAGTAACATCATCCGGGGCTGGATCTTCGGCACCA<br>CCCTGGATAGCAAAACACAGAGCCTCCTGATCGTGAACAATGCC<br>ACGAACGTCGTGATCAAGGTGTGCGAGTTCCAGTTTTGCAATGA<br>TCCTTTCCTGGGTGTGTACTACCACAAGAACAACAAGAGCTGGA<br>TGGAAAGCGAGTTCAGAGTCTACAGCAGCGCCAACAACTGCACA<br>TTCGAGTACGTCTCTCAGCCTTTTCTGATGGACCTTGAGGGGAAA<br>CAAGGCAACTTCAAGAACCTGAGAGAATTCGTGTTCAAGAACAT<br>CGACGGCTACTTCAAAATCTACTCCAAGCACACACCCATCAACC<br>TGGTCCGGGACCTCCCTCAGGGCTTCAGCGCCCTGGAACCCCTG<br>GTCGACCTGCCCATAGGCATCAACATAACGCGGTTCCAAACCCT<br>GCTGGCCCTGCATAGATCCTACCTGACTCCTGGCGACAGCAGCA<br>GCGGATGGACCGCCGGAGCTGCAGCCTACTATGTGGGTGGCAGG<br>TGGACCTGAGACGGCAT
Part-2	GCATCGTCTCATCGGCACCTGCCACCTGTGGGCTACCTGCAACCT<br>AGAACCTTCCTGCTGAAGTACAACGAGAACGGCACAATCACAGA<br>CGCCGTCGACTGCGCCCTGGACCCTCTCTCTGAGACAAAGTGCA<br>CCCTGAAGTCCTTCACCGTGGAAAAGGGCATCTACCAGACCAGC<br>AACTTCCGGGTGCAGCCTACAGAGAGCATCGTGCGATTTCCAAA<br>CATTACCAACCTCTGCCCCTTCGGCGAGGTGTTTAACGCCACAA<br>GATTTGCCTCCGTTTACGCCTGGAATAGAAAGAGAATCAGCAAT<br>TGTGTGGCCGACTACTCCGTGCTGTATAACAGCGCCTCTTTCAGC<br>ACCTTCAAGTGCTACGGCGTTTCCCCAACAAAGCTGAATGACCT<br>GTGCTTCACCAACGTGTACGCCGACTCCTTCGTAATTAGAGGCG<br>ATGAGGTGCGGCAGATCGCACCAGGCCAGACCGGTAAGATCGCT<br>GACTACAACTATAAGCTGCCTGATGATTTTACAGGCTGCGTGAT<br>CGCCTGGAACTCTAACAACCTGGATAGCAAGGTGGGCGGCAACT<br>ACAACTACCTGTACCGGCTGTTTCGCAAGTCTAACCTGAAACCTT<br>TCGAGAGAGACATCTCCACAGAGATCTACCAGGCCGGTTCTACA<br>CCTTGTAACGGGGTGGAAGGCTTCAACTGTTACTTCCCTCTGCAA<br>AGCTACGGCTTCCAGCCTACCAATGGAGTCGGCTACCAGCCATA<br>CCGGGTGGTCGTGCTGTCCTTCGAGTTACTCCACGCCCCCGCCAG<br>GTGGCAGGTGGACCTGAGACGGCAT
Part-3	GCATCGTCTCATCGGCACCTGCCACCGCCACCGTCTGCGGTCCTA<br>AGAAGTCCACCAATCTGGTTAAGAACAAATGCGTGAACTTCAAC<br>TTCAACGGCCTGACCGGGACCGGCGTGCTGACCGAAAGCAACAA<br>AAAGTTCCTCCCCTTCCAGCAGTTCGGCCGTGATATCGCTGACAC<br>CACAGATGCCGTCAGAGATCCACAGACCCTGGAAATCCTGGATA<br>TTACACCCTGCTCCTTCGGAGGAGTTTCTGTGATCACCCCCGGGA<br>CCAATACCAGCAACCAGGTGGCTGTGCTGTACCAAGGTGTTAAC<br>TGCACCGAGGTTCCTGTGGCCATCCACGCCGATCAGCTGACACC<br>TACTTGGAGAGTGTACTCCACTGGCTCCAATGTGTTCCAGACCA<br>GGGCCGGATGTCTGATCGGCGCCGAGCACGTGAATAACAGTTAC<br>GAGTGCGACATCCCTATCGGCGCCGGCATCTGTGCCAGCTACCA<br>GACCCAGACAAACAGCCCTGGGTCTGCTTCCTCTGTAGCTAGCC<br>AGAGCATCATCGCCTACACCATGAGCCTGGGCGCAGAGAACAGC<br>GTGGCCTATTCCAACAACTCTATCGCCATTCCCACCAACTTTACA<br>ATTAGCGTCACAACAGAGATCCTGCCCGTGAGCATGACCAAGAC<br>CAGCGTGGACTGTACAATGTACATCTGTGGCGACAGCACTGAAT

	GCAGCAACCTGCTGCTGCAATACGGCTCCTTTTGCACCCAACTG AACCGGGCGCTGACCGGAATCGCCGTGGAACAGGACAAAAATA CCCAGGAGGTGTTCGCCCAAGTGAAGCAGATCGGTGGCAGGTGG ACCTGAGACGGCAT
Part-4	GCATCGTCTCATCGGCACCTGCCACCGATCTACAAGACCCCACC TATCAAGGACTTCGGCGGCTTTAACTTTAGCCAGATTCTCCCTGA TCCTTCTAAGCCTAGCAAGCGGAGCCCTATCGAGGATCTGCTGTT CAACAAGGTCACCCTGGCCGATGCCGGCTTTATCAAACAGTATG GCGATTGCCTGGGCGACATAGCCGCCAGAGATCTGATCTGCGCC CAGAAATTCAACGGCCTGACAGTTCTCCCACCTCTGCTGACCGA CGAGATGATCGCTCAGTACACCTCTGCCCTGCTGGCTGGCACCA TCACATCTGGGTGGACATTTGGCGCCGGCCCCGCCCTGCAGATC CCCTTTCCCATGCAGATGGCCTATAGATTCAACGGAATCGGCGT GACCCAGAACGTGCTGTATGAAAACCAGAAGCTGATCGCTAACC AGTTCAATTCTGCCATCGGCAAGATCCAGGACTCCCTCTCCTCTA CCCCCAGCGCCCTGGGCAAACTGCAGGACGTGGTGAATCAGAAC GCCCAAGCCCTGAACACCCTGGTGAAGCAGCTCAGCAGCAATTT TGGCGCCATCAGCTCTGTGCTGAACGATATCCTGTCTAGACTGG ACCCTCCAGAAGCCGAAGTCCAGATCGATAGACTGATCACAGGC AGACTGCAGTCCCTGCAAACCTACGTGACCCAACAGCTGATCAG GGCCGCTGAAATAAGAGCCAGCGCCAATCTCGCCGCTACCAAGA TGTCCGAGTGTGTGCTGGGACAGTCTAAACGCGTTGACTTCTGC GGCAAAGGCTATCACCTGATGAGCTTCCCCCAGAGGTGGCAGGT GGACCTGAGACGGCAT
Part-5	GCATCGTCTCATCGGCACCTGCCACCCAGAGCGCGCCGCACGGC GTGGTGTTCCTGCATGTGACATACGTGCCTGCCCAAGAGAAGAA TTTCACAACCGCCCCTGCCATCTGCCACGACGGCAAGGCCCACT TCCCAAGAGAGGGCGTTTTCGTTTCCAATGGCACACACTGGTTC GTGACACAAAGAAACTTCTACGAACCCCAGATTATCACCACCGA CAACACCTTCGTGAGTGGCAATTGTGACGTGGTCATCGGAATCG TGAACAACACAGTGTACGACCCTCTGCAACCTGAGCTGGACTCT TTTAAGGAAGAGCTGGACAAGTACTTTAAAAACCACACCAGCCC CGATGTGGACCTGGGCGACATCAGTGGCATTAACGCCAGCGTGG TGAACATCCAAAAGGAAATCGACAGACTGAACGAGGTGGCCAA GAACCTGAACGAGTCCCTGATCGACCTGCAGGAGCTCGGCAAAT ACGAGCAGGGTGGCAGGTGGACCTGAGACGGCAT

Sequences highlighted in **red** contain BsmBI and AarI recognition sequences as well as the appropriate 5’ and 3’ 4 nt overhangs needed for proper Golden Gate assembly. Simply replace/alter the spike coding sequences in black when designing and ordering gene blocks.

For the assembly of a new Part, the cognate Part-DO vector is required. For example, the assembly of a Part-2 gene block containing a N501Y mutation will require the Part 2 DO vector. These Part + DO vector assemblies should be conducted using the following Golden Gate reaction conditions in PCR strip tubes:

Golden Gate assembly mix (single part):

- 0.25 µL of T7 DNA Ligase (NEB M0318S)
- 0.25 µL of AarI (Thermo Fisher ER1582)
- 1 µL AarI Oligo (1:5 dilution of stock in NFW) (Thermo Fisher)
- 1 µL T4 DNA Ligase Buffer (NEB B0202A)
- 1 µL of the modified Part (10 ng for gene block, 20 ng if in YTK001 plasmid)
- 1 µL of Part DO plasmid (30 ng)
- 5.5 µL of NFW (nuclease free water)

10 µL total reaction volume

Thermocycling (single part):

- 25 cycles
    - 37 °C for 1 minute (digestion)
    - 16 °C for 2 minutes (ligation)
- 1 cycle
    - 37 °C for 30 minutes (final digestion)
    - 80 °C for 20 minutes (heat inactivation)
- 4 °C hold

The Golden Gate cloning framework used for Spike Display allows for the assembly of complex spike variants, containing multiple mutations in different Parts. For example, the alpha variant of concern (VOC), also known as B.1.1.7 contains mutations across all five Parts of the spike coding sequence. To assemble this variant, the Part 1-5 DO vector can be used with customized gene blocks for each of the 5 Parts (see below). These 5 Parts can then be assembled into the Part 1-5 DO vector in a single Golden Gate reaction.

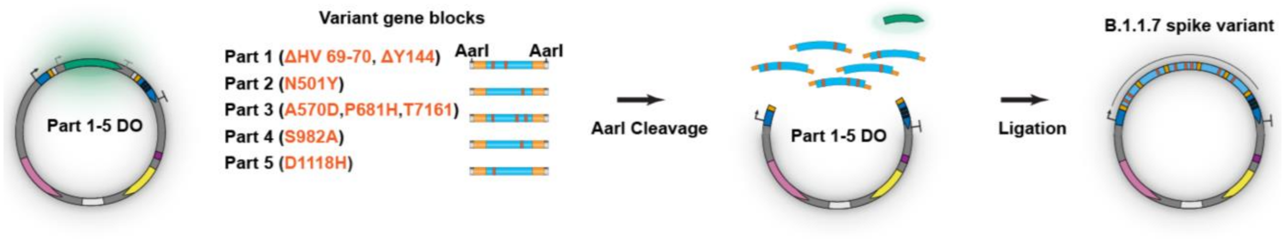

Some spike variants may contain mutations in some Parts but not others. For these assemblies, Part vectors are also available that encode the 6P-D614G spike coding sequences assembled into the YTK001 entry vector (see below). These plasmids can be directly used in the multipart Golden Gate assemblies as they also contain AarI cut sites and the same 4 nt overhangs.

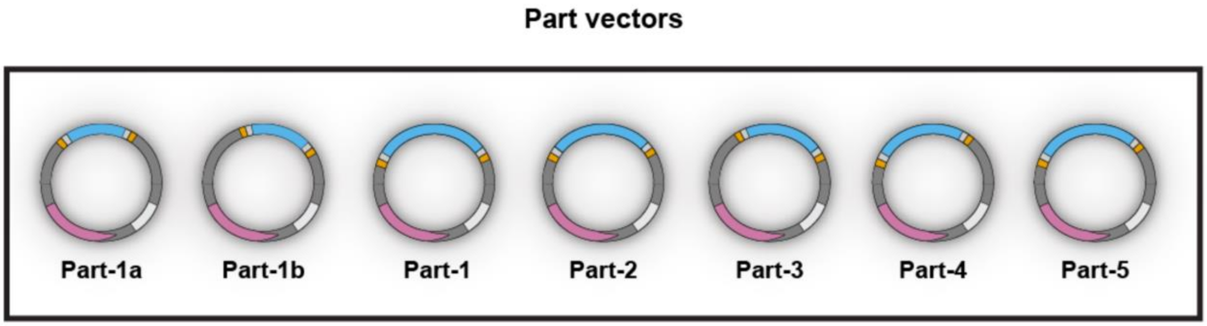

Golden Gate assembly mix (multi-part):

- 0.25 µL of T7 DNA Ligase (NEB M0318S)
- 0.25 µL of AarI (Thermo Fisher ER1582)
- 1 µL AarI Oligo (1:5 dilution of stock in NFW) (Thermo Fisher)
- 1 µL T4 DNA Ligase Buffer (NEB B0202A)
- 1 µL of each modified Part (10 ng for gene block, 20 ng if in YTK001 plasmid)
- 1 µL of Part DO plasmid (30 ng)
- 1.5 µL of NFW (nuclease free water)

10 µL total reaction volume

Thermocycling (multi-part):

- 50 cycles
  - 37 °C for 2 minutes (digestion)
  - 16 °C for 4 minutes (ligation)
- 1 cycle
  - 37 °C for 60 minutes (final digestion)
  - 80 °C for 20 minutes (heat inactivation)
- 4 °C hold

Transformations:

- Thaw out 50 µL of Zymo DH10β Mix & Go Competent Cells (prepared using Zymo T3019) for each plasmid transformation, on ice.
- Transfer 4 µL from each unique assembly to the respective 1.5 mL Eppendorf tube containing 50 µL of competent cells. Flick tubes gently to mix contents and briefly spin down.
- Incubate mixtures on ice for 10 minutes on ice or a 4 °C block.
- Add 150 µL of superior broth (AthenaES 0105) to each tube and perform outgrowth for 1 hour at 37 °C while shaking (tubes on their side for optimal growth/recovery).

- Outgrown cells were plated dropwise on Nunc OmniTrays (5 µL per spot) (Thermo Fisher 140156), containing LB-agar + carbenicillin at 100 µg mL-1. Each plate can fit 96, 5 µL drops. Plates were kept at room temperature until the drops dried and were then transferred to a 37 °C incubator for growth overnight (12-16 hours).
- Colonies should be screened with blue light to discriminate between fluorescent (green/gfp) and non-fluorescent colonies. Colonies still containing the sfGFP-DO and not the desired Part(s) will retain green fluorescence and should not be picked.
- Colonies can be picked into 1 mL of SB media with Carbenicillin (100 µg/mL) in Axygen deep well grow blocks and grown overnight at 37 °C while shaking.
- Once grown, liquid cultures should be pelleted at 3000 g for 10 minutes and miniprepped using the Promega Wizard SV 96 Plasmid DNA Purification Kit (Promega A2250).
- All plasmid assemblies should sequence verified.

Troubleshooting:

- No colonies on the plate:
    - QC competent cells by transforming a Part or Part-DO vector alone. High transformation efficiency commercial competent cells can also be used.
    - Ensure that the correct antibiotics are used for the LB-Agar plate. Part plasmids are Chloramphenicol resistant. Part-DO plasmids are Ampicillin/Carbenicillin resistant.
    - Plate more of the transformed/outgrown cells to compensate for inefficient assemblies.
    - Ensure that the T7 DNA Ligase and T4 DNA Ligase buffer are fresh and have not undergone excessive freeze thaw cycles.
- All colonies are green:
    - Check the concentrations of each Part and Part-DO vector to ensure that correct input concentrations are used for the Golden Gate assembly. These reactions are sensitive to the molar ratio of each DNA component.
    - QC the restriction endonuclease being used with the Part and Part-DO vectors. Run cleaved products on a gel to ensure the separation of insert and backbone. If this reaction fails, either the endonuclease is inactive or the plasmids preparations have impurities, inhibiting the digestion reactions.
    - If using AarI, ensure that the AarI oligo is being added to the reaction. Unlike other endonucleases AarI requires this trigger oligo for efficient cleavage activity.
- Incomplete multi-part assemblies:
    - On occasion, promiscuous cleavage/ligation events will occur during multi-part assemblies causing the dropout of individual Parts. For inefficient multi-part assemblies screen 4 or more colonies and sequences across the entire spike coding sequence.

Protocol 1B: Transient expression of Spike Display in human cell culture

Culturing and seeding cells:

- HEK293T cells should be cultured in:
  - DMEM (Gibco 11995065) containing phenol red, 4 mM L-glutamine, 110 mg L-1 sodium pyruvate, 4.5 g L-1 D-glucose
  - 10% FBS (Gibco 26140079)
  - 2% Pen/Strep (Thermo Fisher 15070063)
- Cells lines should be tested for mycoplasma contamination before use. The Mycoplasma Detection Kit (SouthernBiotech 13100-01) is highly recommended.
- Cells should be maintained in a humidified atmosphere of 5% CO_2 and 37 °C.
- Passage cells every 2-3 days into 10 cm polystyrene coated plates (VWR 10062-880) upon reaching high density.
- Approximately 24 hours before transfection, cells should be seeded into 6-well or 12-well polystyrene coated plates (VWR 10861-696, 10861-698) at a density of 0.3 x 10^6 cells mL^{-1} or 0.1 x 10^6 cells mL^{-1}, respectively.

Transfection:

- Upon reaching 60-80% confluence, cells are ready for transfection.
- For each Spike Display expression vector, we recommend transfecting 2-3 biological replicates (2-3 separate wells of seeded cells).
- For each sample/plasmid set up a sterile Eppendorf tube with Opti-MEM (Gibco 51985091).
  - 100 µL/tube for 12-well plates
  - 200 µL/tube for 6-well plates
- Add endotoxin free plasmid DNA to each tube.
  - 1 µg/tube for 12-well plates
  - 2 µg/tube for 6-well plates
- In a separate Eppendorf tube create a master mix containing:
  - 100 µL or 200 µL of Opti-MEM per sample, for 12- and 6-well plates respectively
  - 3 µL of Lipofectamine 3000 (Thermo Fisher L3000015) per ug of plasmid DNA
  - Recommendation: include 10% excess of each reagent to compensate for solution loss during pipetting.
- Add the Opti-MEM/Lipofectamine master mix to each tube containing plasmid DNA + Opti-MEM
  - 100 µL/tube for 12-well plates
  - 200 µL/tube for 6-well plates
- Incubate reactions in the hood, for 20 minutes. Shorter or longer incubations are detrimental to transfection efficiencies.
- Add each transfection mix (plasmid DNA + Lipofectamine + Opti-MEM) to the appropriate wells containing seeded cells (60-80% confluence) in a drop-wise fashion.
- Return plate(s) to the 37 °C incubator and wait 48 hrs before collecting cells. This will give the spike proteins sufficient time to be post translationally modified, trafficked, and displayed on the cell surface.

Protocol 1C: HEK292T immunostaining and flow cytometry

Collecting cells:

- HEK293T cells with surface-displayed spike should be collected 48 hours post-transfection
- First, wash once with PBS (volume equivalent to the media in each well).
- Next, resuspend in PBS by gentle pipetting until all the cells have been dislodged from the cell surface and are essentially monodispersed (no clusters). HEK293Ts tend to stick and aggregate.
- Transfer resuspended cells from each well to a sterile Eppendorf tube (1 tube per sample).
- Use 20 uL of the resuspended cells to determine cell density. An automated cell counter (Logos Biosystems L40002) was used for this study.
- Cells should then be spun down at 200 g for 1 minute.
- After decanting the supernatant resuspend the cells with chilled PBS-BSA (1% BSA (Sigma-Aldrich A3294), 1x PBS, 2 mM EDTA pH 7.4) to a density of ~3 x 10^6 cells mL^{-1}. Lower cell densities can be used if necessary.
- Cells can be kept in PBS-BSA for several hours if chilled (4 °C). However, for best immunostaining and flow cytometry results, assay cells immediately after collection.

Immunostaining cells:

- Flow cytometry assays can be prepared in Axygen Deep well grow blocks (P-2ML-SQ-C-S).
- Each well contains a predetermined concentration of primary antibody or chimeric cell receptor (ACE2-Fc) diluted in PBS-BSA (500 uL) and 50 μL (1.5 x 10^5) of cells.
- Mixtures should be incubated at room temperature and shaken at 950 rpm for 1 hour. This will provide sufficient time for antibodies or soluble proteins to bind the displayed spike antigen.
- Cells are then pelleted by spinning the plate for 2 minutes at 500 g in a swinging bucket rotor.
- Wash cells by carefully decanting the supernatant and adding 500 μL of PBS-BSA to each well. Spin cells again at 500 g for two minutes and repeat wash.
- 500 μL of a secondary antibody solution (5 μM Alexa Fluor® 488 anti-mouse (SouthernBiotech 1031-30) and 10 μM Alexa Fluor® 647 anti-human (SouthernBiotech 2048-31) in PBS-BSA) was added to each well.
- Incubate the plate in the dark, at 4 ˚C while shaking at 950 g for 25 minutes. Longer incubation times will result in the gradual dissociation of the primary antibodies. For antibodies or soluble cell receptors with weak binding affinities, consider primary labels to improve fluorescent signal while analyzing via flow cytometry.
- Wash the wells again, twice with PBS-BSA, and then resuspended in 300 μL of PBS-BSA before running on the SA3800 Spectral Analyzer (SONY).

Flow cytometry:

HEK293T cells were used to establish forward scatter-area (FSC-A) and side scatter-area (SSC-A) gating (see below). Singlet discrimination was then established with forward scatter-height (FSC-H) vs forward scatter-area (FSC-A) and side scatter-height (SSC-H) vs side scatter-area (SSC-A) gates. A minimum of 10,000 singlet events were acquired for each assayed sample. Variations of these gates and cell counts can be used for each assay if need. However, using a template for consistent gating and data analysis is recommended.

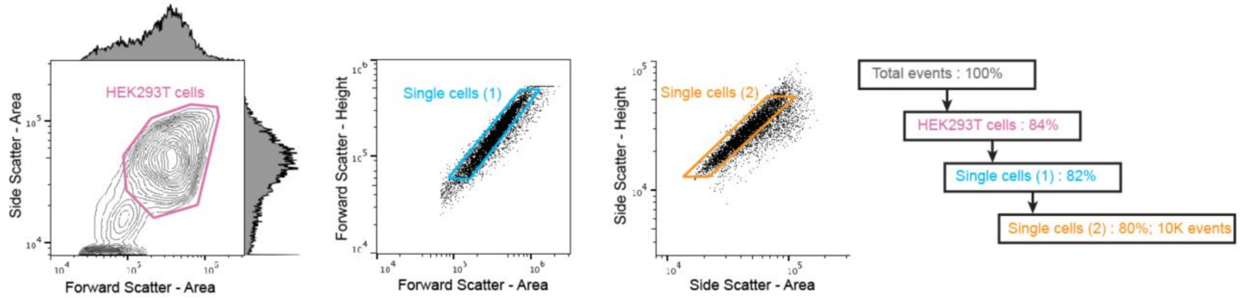

These singlet HEK293T cells are further analyzed in two fluorescent channels, Alexa Fluor 488 (AF-488) and Alexa Fluor 647 (AF-647), using manufacturer-recommended excitation and detection settings.

Spectral unmixing should be applied to all data to reduce the effect of spectral spillover and autofluorescence on downstream calculations. The protocol for spectral unmixing and the creation of fluorescent compensation matrixes will vary depending on the flow cytometer and the software used to analyze the data.

See below for a graphical representation of the antibodies used (left) and the resulting flow cytometry data measuring ACE2 binding of 6P-D614G and B.1.1.7 spike variants (right). Single cells from the gating workflow described above were analyzed using two fluorescent channels, Alexa Fluor-488 and Alexa Fluor-647 for measuring spike expression and antigenicity, respectively.

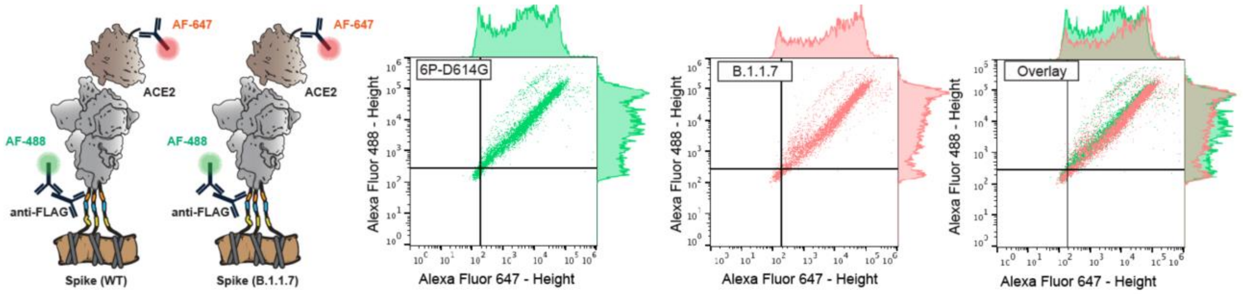

A similar graphical representation is shown for a different antibody (4A8), which interacts with the SARS-CoV-2 spike proteins N-terminal domain (NTD) (see below). Unlike ACE2, 4A8’s binding profile (AF-647 signal) differs greatly between the WT (6P-D614G) and B.1.1.7 spike variants.

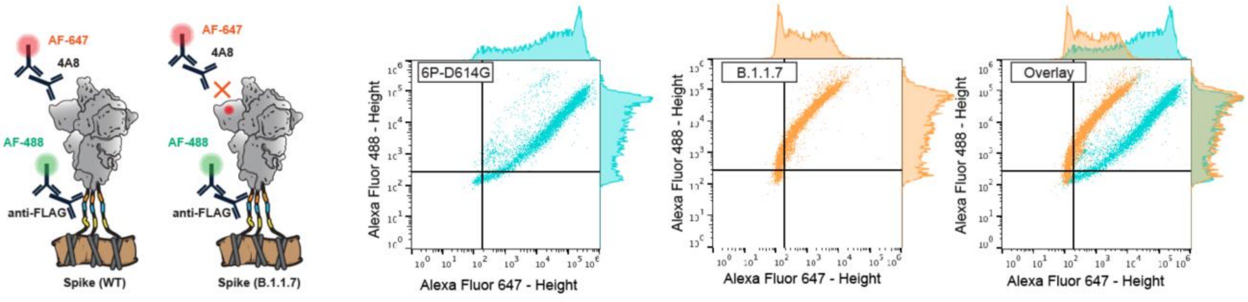

Data analysis:

Median height (H) measurements for the AF-488 and AF-647 channels should be recorded for each sample. Anti-FLAG (AF-488 channel) signal is used to measure spike expression. Spike variant (x) expression relative to WT (6P-D614G) can be calculated using the following equation:

$$Normalized\ expression = \ Log_2(Median{:}\,488\_H_x/Median{:}\,488\_H_{6P\_D614G}$$

Anti-FLAG signal is also used as an internal normalization control to correct for changes in transfection efficiency and spike expression when measuring antibody or ACE2 binding. Normalized binding measurements for spike variants (x) expression relative to WT (6P-D614G) can be calculated using the following equation:

$$Normalized\ binding = \ Log_2\left(\frac{Median{:}\,647\_H_x/Median{:}\,488\_H_x}{Median{:}\,647\_H_{6P\_D614G}/Median{:}\,488\_H_{6P\_D614G}}\right)$$

For antibody titration curves, median height (H) measurements for AF-647 are divided by the respective AF-488 signal for each sample. This provides a normalized binding measurement relative to each sample's expression signal (Anti-FLAG; AF-488). All titrations were conducted using biological triplicate (three separate transfections). All titration points for each curve were then divided by the max value in the set, thus normalizing the curve to a range between 0 and 1 normalized binding units. These values can then fit with a Sigmoidal, 4PL function where X is concentration (least squares fit).

All flow cytometry data for this study was analyzed using FlowJo v9.
