# Supplementary material for: Rapid characterization of spike variants via mammalian cell surface display
Source: Mol Cell. 2021 Dec 16;81(24):5099–5111.e8. doi: 10.1016/j.molcel.2021.11.024 (PMC8675084; doi:10.1016/j.molcel.2021.11.024)
Supplement: Document S2. Article plus supplemental information [file mmc3.pdf]

Technology

Molecular Cell

Rapid characterization of spike variants via mammalian cell surface display

Graphical abstract

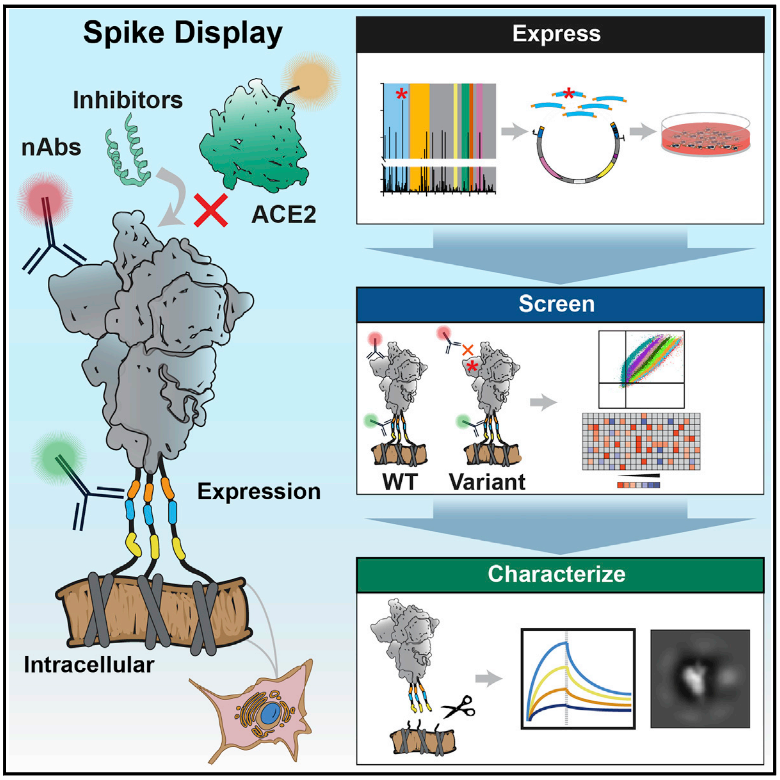

Highlights

- Spike Display accelerates genotype-to-phenotype studies of SARS-CoV-2 spike protein
- N-terminal domain loops N1, N3, and N5 form a public epitope for neutralizing antibodies
- Variants of concern escape several classes of NTD-targeting neutralizing antibodies
- An ACE2 peptide mimic has lost potency against several broadly circulating variants

Authors

Kamyab Javanmardi, Chia-Wei Chou, Cynthia I. Terrace, ..., Kevin N. Dalby, Jimmy D. Gollihar, Ilya J. Finkelstein

Correspondence

kjavanmardi@utexas.edu (K.J.), ilya@finkelsteinlab.org (I.J.F.)

In brief

The SARS-CoV-2 spike protein is a critical target of the immune system. Javanmardi et al. report a mammalian cell display platform for rapidly screening the antigenicity and ACE2 affinity of spike variants. This platform, termed Spike Display, accelerates antigen design and antibody epitope mapping for viral glycoproteins.

Javanmardi et al., 2021, Molecular Cell *81*, 5099–5111
December 16, 2021 © 2021 Published by Elsevier Inc.
https://doi.org/10.1016/j.molcel.2021.11.024

CellPress

Molecular Cell

CellPress

Technology

Rapid characterization of spike variants via mammalian cell surface display

Kamyab Javanmardi,[1,*] Chia-Wei Chou,[1] Cynthia I. Terrace,[2] Ankur Annapareddy,[1] Tamer S. Kaoud,[3] Qingqing Guo,[1] Josh Lutgens,[1] Hayley Zorkic,[1] Andrew P. Horton,[1] Elizabeth C. Gardner,[1] Giaochau Nguyen,[1] Daniel R. Boutz,[2] Jule Goike,[1] William N. Voss,[1] Hung-Che Kuo,[1] Kevin N. Dalby,[3] Jimmy D. Gollihar,[1,2,4,5] and Ilya J. Finkelstein[1,5,6,*]

[1]Department of Molecular Biosciences, The University of Texas at Austin, Austin, TX 78712, USA

[2]CCDC Army Research Laboratory-South, Austin, TX, USA

[3]Division of Chemical Biology and Medicinal Chemistry, College of Pharmacy, The University of Texas at Austin, Austin, TX, USA

[4]Center for Molecular and Translational Human Infectious Diseases Research, Department of Pathology and Genomic Medicine, Houston Methodist Research Institute, Houston Methodist Hospital, Houston, TX, USA

[5]Center for Systems and Synthetic Biology, The University of Texas at Austin, Austin, TX, USA

[6]Lead contact

*Correspondence: kjavanmardi@utexas.edu (K.J.), ilya@finkelsteinlab.org (I.J.F.)

https://doi.org/10.1016/j.molcel.2021.11.024

SUMMARY

The SARS-CoV-2 spike protein is a critical component of vaccines and a target for neutralizing monoclonal antibodies (nAbs). Spike is also undergoing immunogenic selection with variants that increase infectivity and partially escape convalescent plasma. Here, we describe Spike Display, a high-throughput platform to rapidly characterize glycosylated spike ectodomains across multiple coronavirus-family proteins. We assayed ∼200 variant SARS-CoV-2 spikes for their expression, ACE2 binding, and recognition by 13 nAbs. An alanine scan of all five N-terminal domain (NTD) loops highlights a public epitope in the N1, N3, and N5 loops recognized by most NTD-binding nAbs. NTD mutations in variants of concern B.1.1.7 (alpha), B.1.351 (beta), B.1.1.28 (gamma), B.1.427/B.1.429 (epsilon), and B.1.617.2 (delta) impact spike expression and escape most NTD-targeting nAbs. Finally, B.1.351 and B.1.1.28 completely escape a potent ACE2 mimic. We anticipate that Spike Display will accelerate antigen design, deep scanning mutagenesis, and antibody epitope mapping for SARS-CoV-2 and other emerging viral threats.

INTRODUCTION

Severe acute respiratory syndrome coronavirus 2 (SARS-CoV-2) is the causative agent of the COVID-19 pandemic, causing > 200 million infections and > 4.3 million deaths worldwide (as of August 14, 2021). Related betacoronaviruses SARS-CoV-1 and Middle Eastern Respiratory Syndrome (MERS) have also caused epidemics, in 2002 and 2012, respectively (Abdelrahman et al., 2020; Peiris et al., 2003; Zumla et al., 2015). Human coronavirus (HKU1), first discovered in 2004, often manifests as a mild upper respiratory disease (Woo et al., 2005). The large reservoir of diverse and endemic coronaviruses in animals, and their frequent zoonotic transmission, suggests that future human outbreaks are inevitable (Grange et al., 2021; Kreuder Johnson et al., 2015; Li et al., 2020).

Coronaviruses infect cells via attachment of viral transmembrane spike (S) glycoproteins (Li, 2016). SARS-CoV-2 spike interacts with angiotensin-converting enzyme 2 (ACE2) and other cell surface receptors to mediate fusion between the virus envelope and cell membrane (Cantuti-Castelvetri et al., 2020; Yan et al., 2020; Zhang et al., 2020). Spike homotrimers consist of the S1 and S2 functional subdomains (Wrapp et al., 2020). After spike binds ACE2, structural rearrangements in the spike and cleavage by host proteases separate the S1 subunit from the S2 stalk (Li, 2016). The S2 stalk then undergoes further conformational changes that lead to membrane fusion and cell entry. The S1 subunit, which is composed of the N-terminal domain (NTD) and receptor-binding domain (RBD) (Wrapp et al., 2020), is the key determinant of tissue and host tropism (Li, 2016).

Humoral immunity to the spike glycoprotein is the most potent means of protection from SARS-CoV-2 (McMahan et al., 2021). SARS-CoV-2 vaccines generate a strong polyclonal antibody response by delivering spikes via immunization (Baden et al., 2020; Polack et al., 2020; Yu et al., 2020). Spike is also the primary target for prophylactic and therapeutic neutralizing monoclonal antibodies (nAbs) and ACE2 binding inhibitors (Cao et al., 2020; Chan et al., 2021, 2020; Linsky et al., 2020; Piccoli et al., 2020). However, spike mutates and recombines, establishing new variants for immunogenic selection (Bobay et al., 2020). Multiple variants of concern (VOCs) have increased viral transmissibility and antibody escape (Bobay et al., 2020; Li et al., 2020). Since the emergence of a globally dominant D614G

Check for updates

mutation (Korber et al., 2020; Long et al., 2020; Yurkovetskiy et al., 2020), newer VOCs with compound spike mutations have taken hold. The B.1.1.7 (alpha) (Davies et al., 2021; Leung et al., 2021), B.1.351 (beta) (Wang et al., 2021), B.1.1.28 (gamma) (Hoffmann et al., 2021), B.1.427/B.1.429 (epsilon) (Deng et al., 2021; McCallum et al., 2021a), and B.1.617.2 (delta) (Planas et al., 2021) lineages are of particular concern because they partially evade monoclonal antibodies, convalescent sera, and vaccine-induced humoral immunity (Garcia-Beltran et al., 2021). The antigenicity and infectivity of new virus variants are often assayed via live virus, pseudotyped virus, and animal protection experiments. These assays are low throughput and require lengthy viral preparations (Wang et al., 2021).

Cell surface display of the spike protein or its subdomains is a high-throughput platform approach to functionally characterize key aspects of SARS-CoV-2 variants (Starr et al., 2020; Wagner et al., 2019). For example, Starr et al. and others have expressed the RBD on the surface of yeast cells to measure the subdomain expression, ACE2 binding affinity, and RBD-targeting nAb escape (Greaney et al., 2021b, 2021a; Linsky et al., 2020; Starr et al., 2021; Urdaniz et al., 2021). However, all spike VOCs include critical mutations that are outside the RBD. Moreover, the humoral immune response produces potent nAbs that target the NTD as well as the RBD. Here, we describe a new experimental platform that measures spike expression, receptor binding, and antibody escape across variant spike homotrimers on the surface of mammalian cells.

Spike Display is a high-throughput platform to characterize spike glycoproteins from diverse coronavirus families. Complex spike variants are displayed on the surface of mammalian cells and assayed via flow cytometry. Spikes can be cleaved from cell surfaces to further accelerate structural and biophysical characterization. Using this platform, we mapped an NTD supersite that is recognized by the majority of NTD-directed nAbs. We also characterized the individual mutations composing the B.1.1.7, B.1.351, B.1.1.28, B.1.427/B.1.429, and B.1.617.2 variants using flow cytometry and biolayer interferometry (BLI). All five complex variants show escape from NTD-targeting nAbs, whereas B.1.351, B.1.1248, and to a lesser degree B.1.427/B.1.429 escape some RBD-targeting nAbs. Destabilizing mutations such as Δ242-244 and R246I in B.1.351, which enable nAb escape, are compensated for by stabilizing mutations D215G and K417N. The conserved N501Y mutation found in B.1.1.7, B.1.351, and B.1.1.28 also increases ACE2 binding affinity. Most VOCs also escape the ACE2-mimetic LCB1 peptide, suggesting that micropeptide inhibitors must be updated or used as part of a multi-component inhibitor cocktail (Cao et al., 2020). We anticipate that Spike Display will accelerate antigen design, spike characterization, and epitope mapping to aid ongoing and future pandemic countermeasures.

DESIGN

Pandemic countermeasures require the rapid design of antigens for vaccines, profiling patient antibody responses, and the surveillance of emerging viral lineages. Cell surface display can facilitate these goals by coupling the phenotypes of protein variants to their DNA sequence. Screening surface-displayed proteins via flow cytometry also eliminates time-consuming protein purification steps. *S. cerevisiae* is an attractive display chassis that has been used for epitope mapping and deep mutational scanning (DMS) of the SARS-CoV-2 spike RBD (Greaney et al., 2021b, 2021a; Starr et al., 2021, 2020; Urdaniz et al., 2021). These experiments provide valuable insight into the mechanisms for viral evolution and immune escape but also face several limitations. First, yeast is unable to produce full-length spikes. The RBD constitutes only one domain of a highly dynamic and complex homotrimer. Antibodies that target the spike NTD, S2, and other regions outside the RBD are an important source of nAbs and, in turn, viral escape strategies. Second, antigens produced in yeast do not recapitulate mammalian glycosylation (Hamilton et al., 2003). These differences may alter a protein's antigenicity toward cell receptors and antibodies (Grant et al., 2020). To overcome these limitations, we developed a mammalian cell surface display platform for viral glycoproteins.

Spike Display is designed for phenotypic screening of full-length viral glycoproteins on the surface of mammalian cells. As a proof of principle, we displayed the SARS-CoV-2 spike protein on the surface of human embryonic kidney (HEK293T) cells. HEK293Ts and other mammalian cell lines express the spike homotrimers with glycosylation patterns comparable to authentic viral spike proteins (Allen et al., 2021; Yao et al., 2020). We use a SARS-CoV-2 spike ectodomain coding sequence (residues 1–1208) containing six pre-fusion stabilizing prolines (Hsieh et al., 2020; Wrapp et al., 2020) and a mutated furin cleavage site to improve spike stability and expression. The promoters, chimeric introns, and terminators were also optimized to further boost protein expression in mammalian cells. Combinations of N-terminal secretion tags and C-terminal linkers were screened for optimal surface display density. Due to the variability of plasmid transfections in mammalian cell cultures, we included a triple FLAG epitope tag as a proxy for spike expression levels and as an internal control for signal normalization. Transfected cells expressing spikes can be immunostained and analyzed by flow cytometry. A 3C protease cleavage site and a Strep II tag are included in the C-terminal linker to enable the cleavage and rapid purification of surface-displayed spikes. This allows users to characterize spike variants cleaved from cell surfaces using conventional biochemical and biophysical methods (i.e., negative-stain electron microscopy [nsEM] and BLI). We anticipate that Spike Display will be a valuable tool for current and future pandemic countermeasures.

RESULTS

Assessing spike variants on mammalian cell surfaces

We express the SARS-CoV-2 spike ectodomain on the surface of human embryonic kidney (HEK293T) cells. Six proline substitutions stabilize the homotrimeric complex in the pre-fusion state, along with the globally dominant D614G mutation (termed 6P-D614G) (Hsieh et al., 2020; Korber et al., 2020; Long et al., 2020). Spike is directed to cell membranes via an N-terminal Ig kappa secretion signal and a C-terminal PDGFR-β transmembrane domain (Lim et al., 2013). The 58 amino acid (aa) flexible linker includes a triple FLAG (3xFLAG) epitope tag as a proxy for expression, a StrepII tag for purification, and a 3C protease

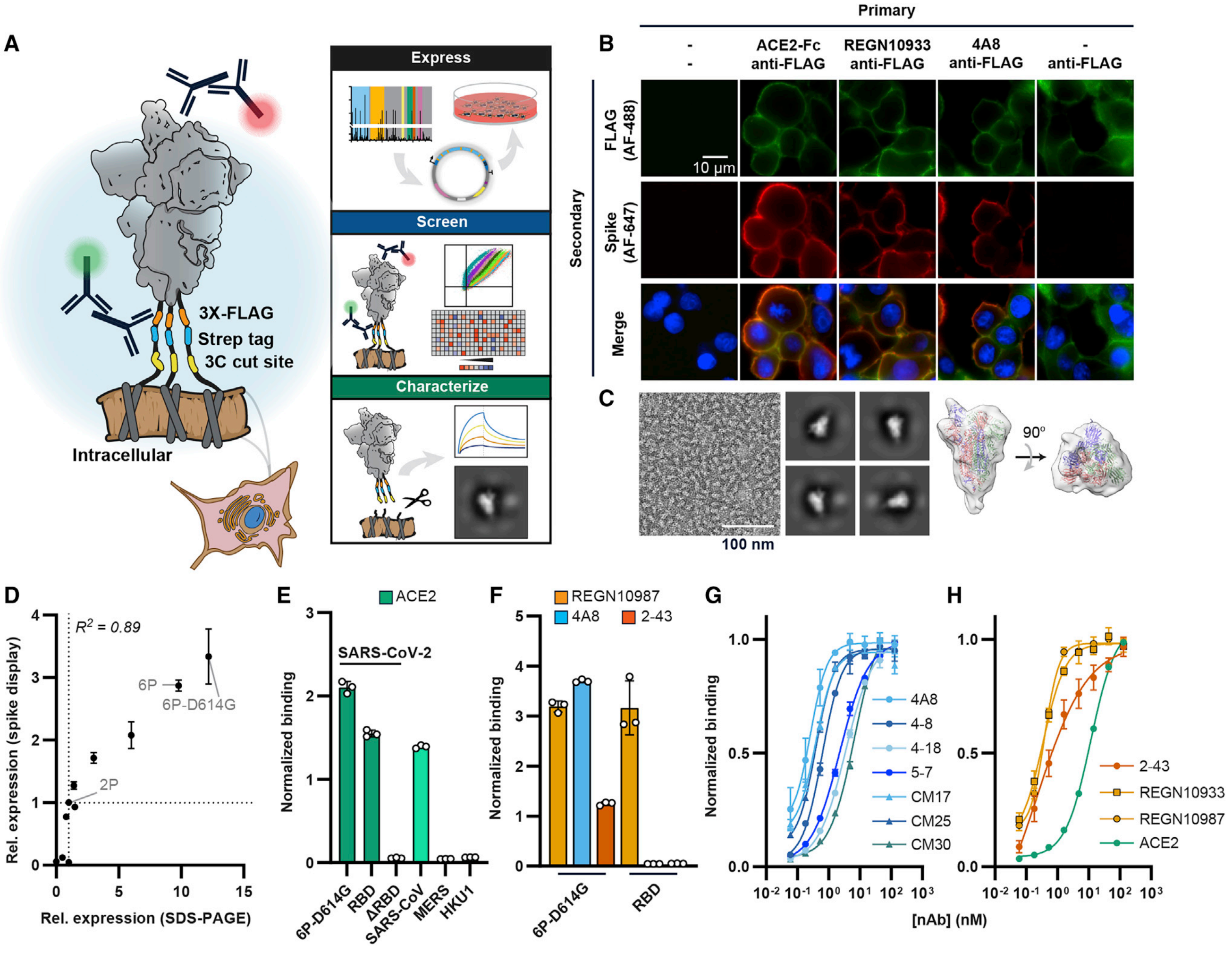

Figure 1. Biophysical characterization of spikes displayed on human cells

(A) Spike ectodomains are displayed on the surface of HEK293T cells. An automated cloning pipeline is coupled with flow cytometry to enable high-throughput screening. Biophysical characterization is performed with spikes cleaved from cell surfaces.

(B) Immunostaining confirms that SARS-CoV-2 (6P-D614G) spikes are localized to cell membranes and bind ACE2 (RBD-directed), REGN10933 (RBD-directed), or 4A8 (NTD-directed). Scale bar, 10 μm.

(C) Negative-stain electron microscopy micrograph (left), 2D class averages (middle), and a 3D model of surface-displayed spikes in the pre-fusion conformation. The majority of particles show a "one RBD up" configuration. Scale bar, 100 nm.

(D) Relative Spike Display signal correlates with recombinant spike expression levels for engineered and clinical spike variants (Hsieh et al., 2020; Long et al., 2020). For both axes, the signal is normalized to spike-2P (two prolines) expression. Pearson correlation is used for statistical analysis.

(E) ACE2 soluble domain binding by diverse coronavirus-family spikes displayed on mammalian cell surfaces.

(F) Normalized binding for RBD- (REGN10987), NTD- (4A8), and S1-directed (2-43) neutralizing antibodies was measured using either the full SARS-CoV-2 spike or the isolated RBD.

(G) Titration of NTD-binding nAbs using Spike Display and flow cytometry.

(H) Titration of ACE2, RBD-directed (REGN10933 and REGN10987), and S1-directed (2-43) nAbs using Spike Display and flow cytometry.

All measurements in (E)–(H) are an average of three biological replicates. Error bars, SD.

cleavage site (Figure 1A). Immunostained fixed cells show spike at the cell membrane (Figure 1B; Figures S1A and S1B). We confirmed the native homotrimeric assembly via nsEM of spikes that were cleaved from the cell surface by 3C protease (Figure 1C). Two-dimensional class averages indicate that > 99% of the surface-displayed spikes are in the pre-fusion conformation, with the majority (71%) of the particles having one RBD up (Figure S1C). These results are consistent with previous assessments of HexaPro (6P) and D614G spike RBD up-down equilibrium (Hsieh et al., 2020; Wrapp et al., 2020; Yurkovetskiy et al., 2020).

We assessed spike expression and antigenicity via flow cytometry. SARS-CoV-2 spike variants and related spike homologs were rapidly constructed via Golden Gate assembly from standardized parts and acoustic liquid handling (Figure 1A; STAR Methods). Each variant was transfected into HEK293T

cells and stained with anti-FLAG and fluorescent secondary (anti-mouse) antibodies. Mutant SARS-CoV-2 spike expression on the surface of mammalian cells strongly correlated with the relative expression of recombinant spike mutants (Figure 1D) (Hsieh et al., 2020; Long et al., 2020). The relative expression of spikes from related betacoronaviruses SARS-CoV-1, MERS, and HKU1 correlated with their reported stability from prior studies (Figures S1D–S1F) (Hsieh et al., 2020; Kirchdoerfer et al., 2016; Pallesen et al., 2017; Wrapp et al., 2020). We next tested whether spike display can be used for antigen design by testing pre-fusion stabilizing proline substitutions into SARS-CoV-1 spike (Figures S1G and S1H). Spike expression improved 5-fold, suggesting further stabilization over the parental SARS-CoV-1 construct. We conclude that Spike Display can be used to rapidly assess the structural expression of coronavirus spike variants and to design next-generation pre-fusion stabilized vaccine targets.

Next, we measured ACE2 binding affinity of surface-displayed spikes. Non-cross-reactive fluorescent secondary antibodies were directed against the human-Fc (ACE2 binding) and mouse-Fc (anti-FLAG; spike expression). This two-color assay enables spike expression-based signal normalization for antigen binding (Figure 1E; Figures S2A–S2C). As expected, SARS-CoV-1, SARS-CoV-2, and the isolated RBD avidly bound ACE2, whereas spike (ΔRBD) did not bind ACE2 above background. The higher affinity of SARS-CoV-2 relative to SARS-CoV-1 spike is consistent with prior *in vitro* measurements with purified proteins (Wrapp et al., 2020). The MERS and HKU1 spikes recognize the human DPP4 receptor and 9-O-acetylated sialic acids, respectively, and have no affinity for ACE2 (Tai et al., 2020).

Spike Display enables rapid flow cytometry-based characterization of nAbs (Starr et al., 2020). An estimated 10% of the nAbs in seroconverted COVID-19 patients bind outside of the RBD, and many RBD binders may bind across multiple subunits (Piccoli et al., 2020; Chi et al., 2020; Liu et al., 2020a; Starr et al., 2020). Therefore, we assayed nAb binding to different spike subdomains. We first tested REGN10987, 4A8, and 2-43, which are RBD-, NTD-, and S1 (quaternary)-binding nAbs, respectively (Figure 1F) (Chi et al., 2020; Hansen et al., 2020; Liu et al., 2020a). The signal generated from human-Fc binding secondary antibodies was normalized to the spike expression signal, enabling accurate binding measurements (STAR Methods). We observed domain-specific nAb binding with full spikes, whereas the RBD alone only bound REGN10987. We next measured quantitative binding affinities of seven NTD-binding nAbs via surface display (Figure 1G; Table S1). We selected nAbs that bind with pM to ~10 nM binding affinities. These peripheral blood mononuclear cell (PBMC)-derived antibodies were identified through diverse discovery platforms, including Ig-seq (Lavinder et al., 2015), and exhibited differing neutralization potencies and NTD-binding epitopes. The binding affinities derived from fitting the sigmoidal titration curves corresponded closely to the affinities measured with recombinant proteins (Figures S2D–S2M). Titration experiments with ACE2, the REGN-COV2 cocktail nAbs (REGN10933 and REGN10987, both binding the RBD), and 2-43 (S1 binder) confirmed that Spike Display is a quantitative platform for measuring antibody and antigen-binding affinities (Figure 1H). Assaying nAb binding on full-length spike ectodomains will thus be a valuable tool for rapid antibody discovery and characterization.

Immune escape from public epitopes in the spike NTD

The SARS-CoV-2 spike NTD elicits high-affinity nAbs in seroconverted and vaccinated patients (Chi et al., 2020; Liu et al., 2020a; Voss et al., 2021). However, 46% of all circulating spike protein mutations (399,299 out of 866,373 total; GISAID database accessed on February 24, 2021) are in the NTD—two-fold higher than a random distribution (expected to be 23%) (Figure 2A; Figure S3; STAR Methods) (Elbe and Buckland-Merrett, 2017). This large number of NTD mutations raises the possibility of immune pressure and subsequent escape.

To elucidate how nAbs bind the NTD, we created high-resolution epitope maps of the five NTD loops, the major target of nearly all nAbs. We cloned 72 alanine spike mutants encompassing all five NTD loops and their adjacent residues (Figure 2B; Figure S5A). Alanine NTD substitutions very mildly destabilized spike expression (Figure S4A). We assayed ten NTD-directed nAbs against this alanine library. The binding site for six of these nAbs has been solved in complex with spike (Cerutti et al., 2021; Chi et al., 2020; Liu et al., 2020a; Voss et al., 2021). In addition to the ten NTD nAbs described above, we also included ACE2 and the RBD-binding REGN10987 nAb as negative controls (Figure 2C).

We first focused on 4A8, which interacts with eight residues in the N3 and N5 loops of the NTD (Chi et al., 2020). Only three of these eight substitutions—Tyr145, Lys147, and Trp152—reduced antibody binding > 5-fold (Figures 2C and 2D). Combining any two substitutions resulted in an additional loss of binding, sometimes to below the detection limit (Figure 2E). Surprisingly, two close contacts in the 4A8 Fab-spike ectodomain structure are dispensable for strong binding. Lys150, which forms a salt bridge with 4A8, only minimally reduced binding affinity. Arg246 on the N5 loop buried in the 4A8-spike interface reduced binding a modest 0.8-fold. Antibodies CM17, CM25, and 1-68 were also sensitive to alanine substitutions at Tyr145, Lys147, and Trp152, suggesting that this loop is a public epitope (Cerutti et al., 2021; McCallum et al., 2021b; Voss et al., 2021). Despite competing with 1-68 in a spike binding assay, 1-87 showed little to no loss in binding from any single-alanine substitution in our library. We speculate that the CDR-H1 and -H3 of the 1-87 are minimally impacted by single alanine substitutions at the NTD surface. Antibodies 5-7 and 4-19 were more sensitive to substitutions in the N1 (residues 14–26) and N5 loops and a region between the N3 and N4 loops. Both of these nAbs have weaker neutralizing IC_{50}s (5-7 = 0.033 $\mu g\ mL^{-1}$ and 4-19 = 0.109 $\mu g\ mL^{-1}$) relative to nAbs, which showed strong N3 loop binding (4-8 = 0.009 $\mu g\ mL^{-1}$, 1-68 = 0.014 $\mu g\ mL^{-1}$, CM25 = 0.012 $\mu g\ mL^{-1}$ IC_{50}s), suggesting that the N3 loop dominates NTD-mediated spike neutralization (Liu et al., 2020a). On the contrary, mutations in the N2 and N4 loops were inconsequential to antibody binding despite their proximity to the NTD supersite. We conclude that Spike Display can provide high-resolution epitope maps that complement structural studies and Fab competition assays.

CellPress

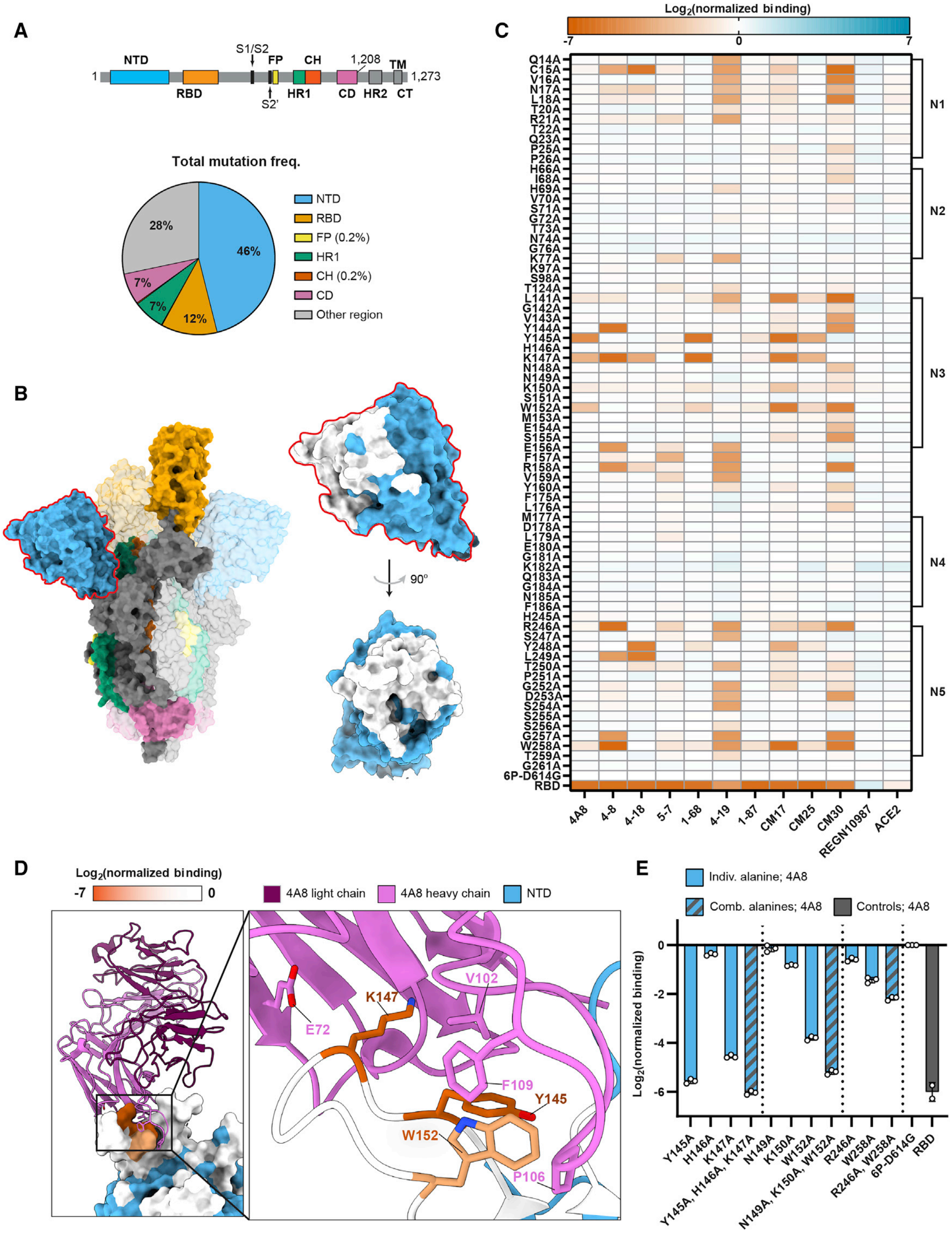

Figure 2. A high-resolution map of NTD-targeting nAb epitopes

(A) Spike domain map (top) (Wrapp et al., 2020) and distribution of all non-synonymous mutations (total = 866,373) found in GISAID (accessed on February 24, 2021) (bottom). The NTD harbors 46% of all mutations while making up 23% of the protein.

(B) Spike trimer structure (PDB: 7DDN [Zhang et al., 2021]) with domains colored as in (A). An enlarged structure of the NTD (blue) with alanine scan positions (white) is shown on the right.

(C) The effect of single alanine substitutions on antibody and ACE2 binding measured by flow cytometry (see STAR Methods and supplemental information for the mean and SD). Red, decreased binding; blue, increased binding, relative to the reference spike (6P-D614G). The RBD is included as a negative control for all NTD-binding antibodies (last row). NTD loops 1–5 are annotated on the right.

(legend continued on next page)

Circulating spike mutants escape NTD- and RBD-directed antibodies

Guided by the alanine scan results, we profiled 46 clinically circulating spike NTD variants for their impact on overall expression and antibody evasion (GISAID database accessed on February 24, 2021). All NTD mutants retained wild-type (WT) levels of ACE2 binding and spike expression (Figure 3A). Substitutions at Tyr145 strongly reduced 4A8 binding, as highlighted by the alanine scan. These polar and charged residues (Asn, Asp, and His) likely disrupt the hydrophobic and/or π/π interactions with 4A8's CDR-3 region. Substitutions at Tyr145 also reduced CM17, CM25, and 1-68 binding. NTD variants that reduced nAb binding > 16-fold appeared at very low frequencies (< 0.001) in the GISAID database. Two noteworthy exceptions include M153T and S254F, both of which show strong escape from CM30 (Figure 3B) (Elbe and Buckland-Merrett, 2017).

Pearson correlation analysis of the NTD alanine and clinical variant binding data showed strong similarities between NTD-binding nAbs and no correlation with REGN10987 (Figure 3C; Figures S5B–S5D). We clustered antibodies into four binding classes based on Pearson correlation (r) values (> 0.5) between their relative binding affinities for the alanine and clinical spike mutants. The largest group consists of 4A8, 1-68, 1-87, CM17, and CM25, all of which originate from the VH1-24 multi-donor class of antibodies (Cerutti et al., 2021; Voss et al., 2021). Interestingly, some antibodies derived from VH3-30 (4-18) have overlapping epitopes with VH1-24-derived nAbs but have significantly different binding modes (Cerutti et al., 2021). ELISA competition assays have implicated distinct binding epitopes for 5-7 and 4-19 compared to the other antibodies in this set (Liu et al., 2020a). Our antibody classifications extend prior structure- and competition-based classifications of NTD-binding antibodies (Cerutti et al., 2021; Liu et al., 2020a).

Deletions in NTD loops N3 and N5 are frequent in circulating viral variants and are possible routes for immune evasion (Figure 3D) (Liu et al., 2020b; McCarthy et al., 2021). Although not yet observed in the GISAID, insertions in these loops may also result in viral escape from nAbs. For example, a lab-evolved SARS-CoV-2 virus with an 11 aa insertion in N5 [$_{248a}$KTRNKSTSRRE$_{248k}$] evaded high titer convalescent sera in an *in vitro* passaging experiment (Andreano et al., 2021). To clarify the escape mechanisms, we assayed these variants against the panel of nAbs described above (Figure 3E). Deletions within the N3 loop (ΔPhe140-Leu141, ΔTyr144/145, and ΔHis146) disrupted binding to most NTD nAbs. A deletion at Phe140, located at the base of the N3 loop, also caused a moderate loss in binding for most of the NTD-binding antibodies. Similarly, a deletion at the base of the N5 loop (ΔAla243-Leu244) and an 11 aa insertion within the N5 loop resulted in reduced binding for most of the nAbs (Figure 3E). A deletion at Ile210 had negligible effects on antibody binding. Together, these results suggest that indels within or adjacent to the N3 and N5 loops effectively abrogate nAb binding, likely due to the deletion of critical residues or spatial reconfigurations of the NTD loops. An insertion in the N5 loop also effectively evades nAbs. As immune escape becomes a major evolutionary factor in an increasingly vaccinated world, NTD insertions may also become more common.

Next, we examined RBD mutations, 11 of which were previously reported to escape therapeutic antibodies from the REGN-COV2 cocktail (REGN10933 and REGN10987) (Figure S6) (Starr et al., 2021). Expression of the full spike trimers and their escape from RBD-binding nAbs correlated well with changes in expression for the RBD alone (Figures S6C–S6E). However, K417N strongly reduced ACE2 binding in the context of the full spike relative to the isolated RBD. Lys417 is sterically occluded when the RBD is in the "down" position, providing a possible explanation for a larger reduction in binding observed with Spike Display compared to yeast display. We also examined a quaternary S1-binding nAb (2-43) and an NTD binder (4A8). As expected, 4A8 binding was not affected by RBD mutations. Three variants (L455A, F486K, and E406W) that reduced REGN10933 binding also reduced 2-43 binding (Figure S6B). These findings underscore the importance of characterizing nAb escape mutations within the context of the fully glycosylated spike trimer.

Characterization of spikes from emerging VOCs

We quantified spike expression, ACE2 affinity, and immune evasion for VOCs (Figure 4). Five lineages—B.1.1.7 (alpha), B.1.351 (beta), B.1.1.28 (gamma), B.1.427/B.1.429 (epsilon), and B.1.617.2 (delta)—harbor eight, nine, ten, three, and seven spike mutations, respectively. Lineage B.1.1.7 increased spike expression 39% relative to the WT (6P-D614G) spike, whereas B.1.351, B.1.1.28, and B.1.427/B.1.429 showed 32%, 47%, and 62% reduction in spike expression, respectively (Figure 4A). All VOCs also harbor the D614G substitution, which significantly boosts spike expression (Figure 1D). K417N (B.1.351) and K417T (B.1.1.28) further positively compensate spike expression in some variants.

We next determine the combined effect of the five VOCs on ACE2 binding and nAb escape (Figures 4B–4F). Lineages B.1.1.7, B.1.351, and B.1.1.28 bound ACE2 with a greater affinity than WT (6P-D614G), with B.1.1.28 showing the greatest increase (1.7-fold over 6P-D614G). Most NTD supersite-targeting nAbs had ~10-fold reduced binding to the B.1.1.7, B.1.351, B.1.427/B.1.429, and B.1.617.2 lineages. BLI using cleaved spikes confirmed the loss of binding across most NTD-binding nAbs (Figures S7A–S7E; Table S2). However, 4-18 and CM30 both retained strong binding to B.1.1.7. Pseudovirus neutralization assays confirmed that 4-18 and CM30 can also prevent cell entry in both the WT and B.1.1.7 strains (Figure 4L; Figures S7F–S7H). Additionally, B.1.351 and B.1.1.28, which have three nearly identical RBD mutations, showed reduced REGN10933 and 2-43 binding. We systematically screened each mutation for all VOCs using 4A8, REGN10933, 2-43, and ACE2 (Figures 4G–4K). Most mutations in the N3 and N5 loops of the NTD (Δ144/145, W152C, Δ242-244, and R246I) reduced 4A8 binding. While other NTD mutations (i.e., L18F, T19R, Δ157, and Δ158)

(D) Co-structure (PDB: 7C2L [Chi et al., 2020]) of the 4A8 Fab (light chain, violet; heavy chain, pink) in complex with the NTD (blue). Alanine scan binding data for 4A8 is superimposed on the NTD and represented on a 0 to −7 binding scale.

(E) Combining multiple alanine mutations abrogates 4A8 binding. RBD is included as a negative control (gray). Mean ± SD of at least two biological replicates.

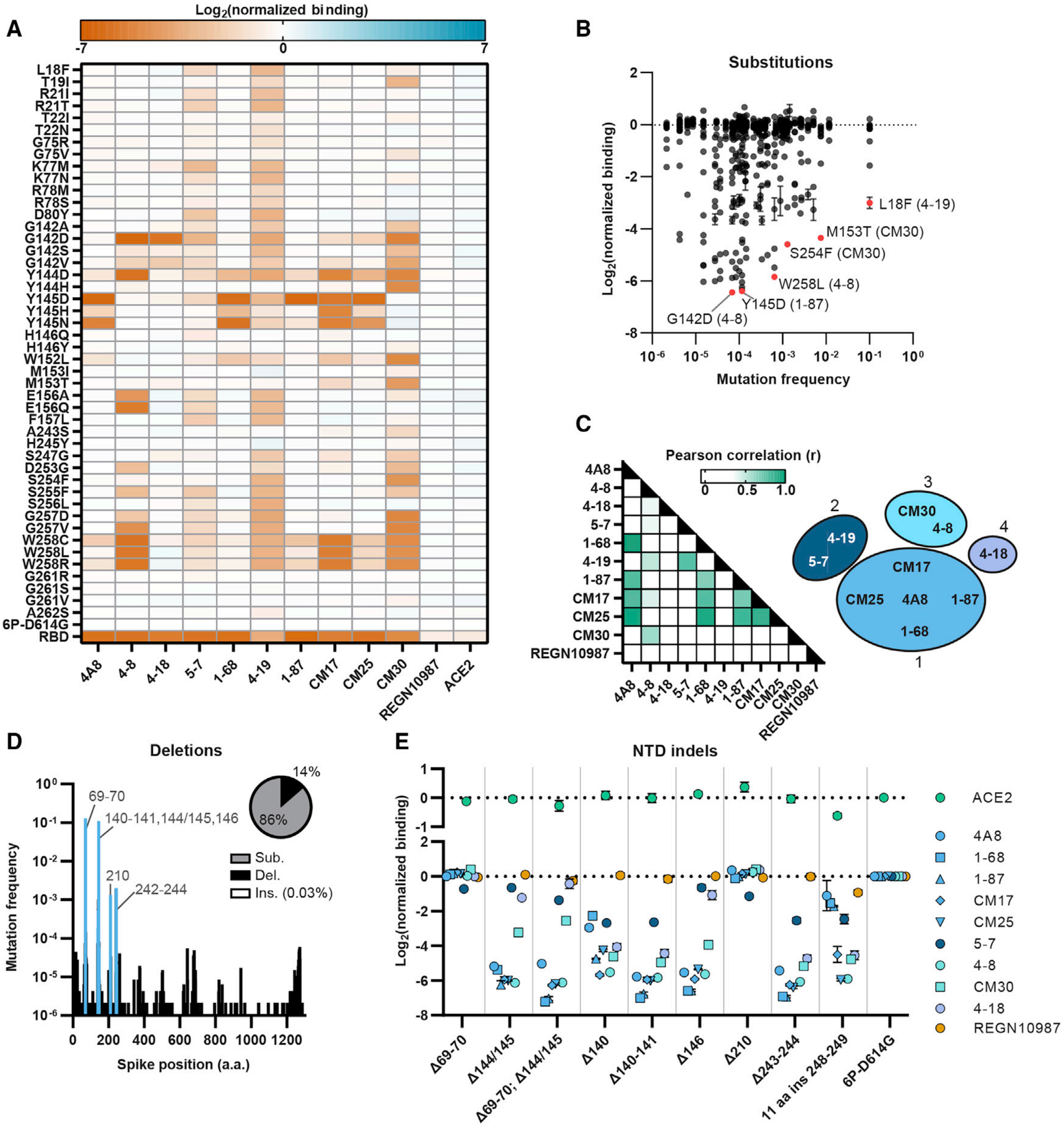

Figure 3. Clinical NTD mutants evade nAb binding

(A) The effect of single amino acid substitutions on antibody or ACE2 binding measured by flow cytometry. Red, decreased binding; blue, increased binding, relative to spike (6P-D614G). RBD is a negative control for all NTD-binding antibodies (last row).

(B) Nearly all nAb-evading NTD mutants occur at a low frequency in the GISAID. Notable mutations and the antibodies they evade are highlighted in red.

(C) Pearson correlation matrix comparing the loss of binding across all antibodies and point mutants (left). The ten NTD-binding antibodies tested cluster in four groups based on r values > 0.5 (right).

(D) Histogram of all deletions located in the spike protein with common NTD deletions (blue). Inset: distribution of all spike substitutions, deletions, and inserts in the GISAID.

(E) Consequences of NTD indels on ACE2 (top) and antibody (bottom) binding (n = 3 biological replicates, mean ± SD reported in supplemental information).

had no detectable effect on 4A8's binding on their own, they evade separate classes of NTD neutralizers, such as 5-7 and 4-19. These nAbs partially bind outside the common N3 and N5 loop supersites. Mutations G142D and E156G have also been observed in the B.1.617.2 lineage. Although these were not included in our B.1.617.2 spike variant, our heatmaps (Figure 2C and Figure 3A) show reduced NTD-targeting nAb binding for mutations at those positions.

The reduction in REGN10933 binding for B.1.351 and B.1.1.28 results from the combined K417N/T and E484K mutations. Mutations S13I and W152C for B1.427/B.1.429 synergistically reduce 4A8 binding, which recapitulates recent findings of nAb

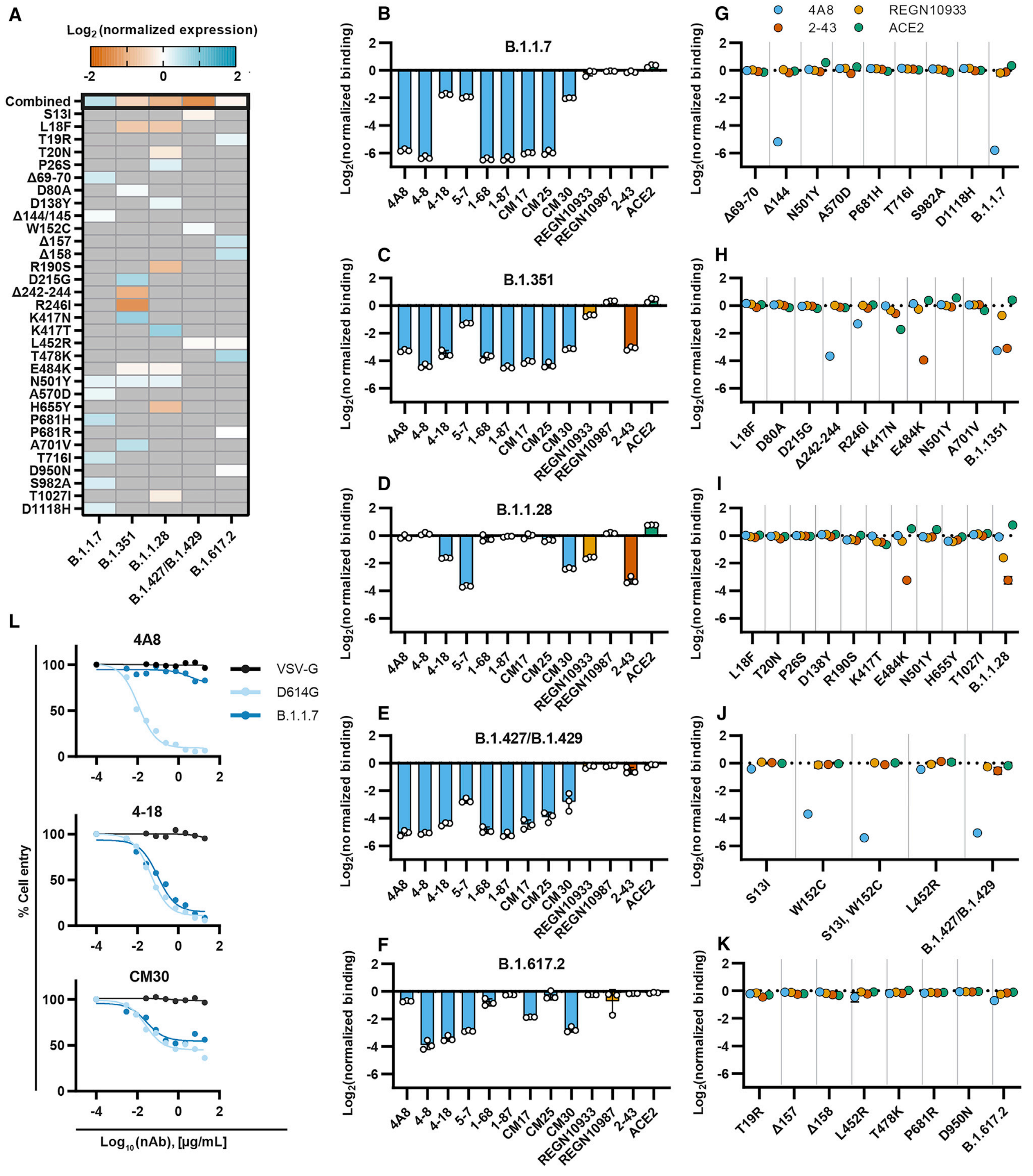

Figure 4. Most variants of concern evade NTD-directed nAbs

(A) Normalized spike expression for four variants of concern and their mutations, measured by flow cytometry. Red, decreased expression; blue, increased expression, relative to spike (6P-D614G). Gray indicates the absence of a mutation in a lineage (mean ± SD of at least twelve biological replicates reported in supplemental information).

(B–F) Relative antibody and ACE2 binding to B.1.1.7 (B), B.1.351 (C), B.1.1.28 (D), B.1.427/B.1.429 (E), and B.1.617.2 (F) as compared to spike (6P-D614G). NTD binders, blue; RBD binders, yellow; S1 binders, red; ACE2, green. Mean ± SD of three biological replicates.

(legend continued on next page)

evasion through novel disulfide bond formation and NTD structural remodeling (McCallum et al., 2021a). Similarly, the net increase in ACE2 binding is due to a combination of E484K and N501Y offsetting the reduced ACE2 binding elicited by the K417N/T. The E484K mutation alone was sufficient to reduce 2-43 binding ~10-fold in both strains. Mutations that reduced nAb binding (Δ242-244 and R246I) were also the most detrimental for spike expression. These results suggest that spike-destabilizing mutations that are beneficial for viral fitness (immune evasion and ACE2 binding) are compensated by other stabilizing mutations. Similar mutation patterns have been described for other viral evolution and escape pathways (Bloom et al., 2010; Friedrich et al., 2004; Wu et al., 2017).

ACE2 mimics are promising SARS-CoV-2 inhibitors, but many were designed against the viral clades circulating in 2019. Their efficacy has not been assessed with modern VOCs (Cao et al., 2020; Chan et al., 2020). We tested how these VOCs bind LCB1, an engineered α-helical peptide that competes with ACE2 for binding to the RBD (Cao et al., 2020). LCB1 has a sub-nM affinity for the RBD *in vitro* and neutralizes live viruses with an IC_{50} of 24 pM (Cao et al., 2020). We developed a competition assay in which cells are co-incubated with increasing concentrations of LCB1 and a fixed ACE2 concentration. Changes in ACE2 binding were then measured by flow cytometry (Figure 5A). Variants B.1.1.7 and B.1.617.2 showed the same sigmoidal curve as the WT (6P-D614G) spike, indicating potent inhibition of ACE2 binding above 1 nM LCB1 (Figure 5C). B.1.351 and B.1.1.28 showed complete escape; ACE2 binding remained unaffected even at > 100 nM (LCB1). The B.1.427/B.1.429 variant showed moderate escape. The K417N/T, E484K, and N501Y point mutations are all in the vicinity of the LCB1-ACE2 interface and are likely to reduce LCB1 binding affinity (Figure 5B). The L452R and T478K mutations are positioned on the periphery of the interface and likely impact LCB1 binding to a lesser degree. Future peptide-based inhibitor therapies will require a cocktail of inhibitors to evade VOCs. Alternatively, these peptides must be continually optimized to improve efficacy against emerging viral variants (Linsky et al., 2020).

DISCUSSION

We developed Spike Display to accelerate vaccine design and to rapidly assess the effects of mutations in emerging virus strains. Pre-fusion stabilized spike is the primary antigen delivered via immunization. Pre-fusion stabilization requires laborious biochemical assays following structure-guided selection of promising stabilizing mutations (Hsieh et al., 2020; Juraszek et al., 2021; Pallesen et al., 2017; Wrapp et al., 2020). Spike Display shortens this workflow by rapidly assessing the expression of individual point mutants and their combinatorial derivatives (Figure 1D). Spikes can be cleaved from cell surfaces for structural and biophysical characterization. We anticipate that Spike Display can be adapted for diverse coronavirus-family spikes and other viral antigens (Figure 1E) (Bruun et al., 2017; Starr et al., 2020; Zhou et al., 2010).

Spike Display complements structure-function studies for high-resolution epitope mapping. We focused on the NTD because it is under heavy mutational pressure in circulating SARS-CoV-2 variants and elicits nAbs in convalescent patients (Chi et al., 2020; Liu et al., 2020a; McCallum et al., 2021b; Suryadevara et al., 2021; Voss et al., 2021). The mechanisms of NTD-mediated viral inhibition remain unknown, but NTD-binding antibodies do not block ACE2 binding. Our NTD scan uncovered that most of the NTD-binding nAbs recognize a public epitope composed of the N3 and N5 loops. These loops may be required for additional conformations that occur after ACE2 recognition. Although circulating variants escape first-generation NTD-binding nAbs, these epitopes are an important target for second-generation antibody therapeutics. We conclude that the NTD is under strong evolutionary pressure and that existing NTD-targeting nAbs must be paired with nAbs that target other epitopes to avoid therapeutic evasion.

Using Spike Display, we screened variants B.1.1.7 (alpha), B.1.351 (beta), B.1.1.28 (gamma), B.1.427/B.1.429 (epsilon), and B.1.617.2 (delta) and their individual mutations (Figure 4). The B.1.1.7, B.1.351, and B.1.1.28 lineages share a common N501Y RBD mutation that likely confers greater transmissibility (Leung et al., 2021; Xie et al., 2021). The B.1.427/B.1.429 variant also exhibits improved transmissibility relative to the parental strain (Deng et al., 2021). Compensatory mutations offset the destabilizing effects of NTD and RBD mutations that partially escape NTD- and RBD-binding nAbs. Variants B.1.1.7, B.1.351, and B.1.427/B.1.429 all escaped NTD-binding nAbs. However, many NTD nAbs are promising therapeutics for B.1.1.28, which does not have any consequential mutations near the NTD supersite. The RBD-binding REGN-COV2 antibody cocktail recognized all variants, with B.1.1.28 showing a modest reduction in binding due to the K417T and H655Y substitutions. B.1.1.7 and B.1.351 also completely evaded a promising micropeptide ACE2 mimic (Figure 5). Future ACE2 mimetic therapies will need to be re-formulated to stay ahead of current and emerging VOCs.

Spike Display accelerates the characterization of antibody-spike interactions and the consequences of non-synonymous spike mutations on these interactions. The entire process, from Golden Gate assembly to flow-based characterization, can be completed in < 5 days. Downstream biophysical studies can use the cell-cleaved spikes without laborious recombinant protein purification. A second-generation Spike Display platform will increase throughput by integrating spike variants in a chromosomal locus and sorting pooled libraries for phenotypic differences (Cheung et al., 2019; Matreyek et al., 2018, 2020). Increasing throughput will allow interrogation of all circulating non-synonymous spike mutations and rapid protein engineering for pre-fusion stabilization. DMS of all possible amino acid substitutions will be broadly useful for characterizing the spike mutational landscape as well the development of pan-coronavirus vaccine antigens.

(G–K). Relative antibody binding measured for each variant's mutations (n = 3 biological replicates, mean ± SD reported in supplemental information).
(L) Pseudovirus neutralization curves comparing D614G (light blue) and B.1.1.7 (dark blue) variants using three neutralizing antibodies (4A8, CM30, and 4-18). VSV-G (black) was included as an infection control (Figures S7F–S7H).

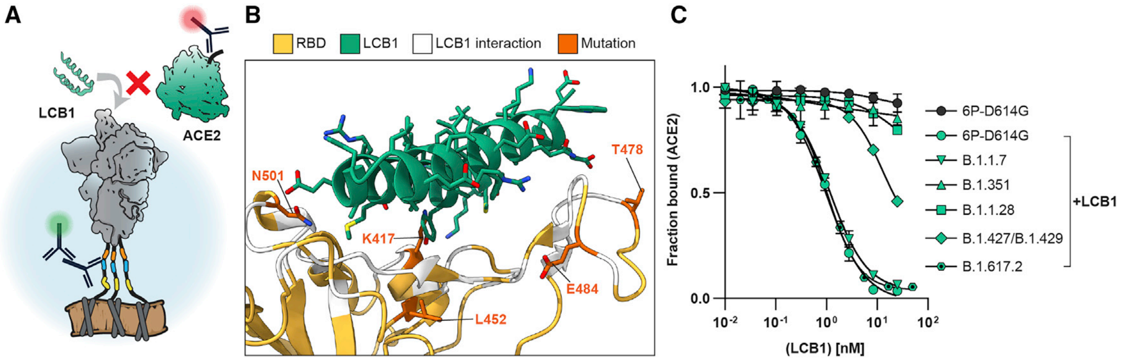

Figure 5. ACE2 mimics lose effectiveness against some variants of concern

(A) Schematic of the LCB1 peptide and ACE2 competing for RBD binding.

(B) Co-structure (PDB: 7JZL [Cao et al., 2020]) of LCB1 (green) binding the RBD (yellow) with contacting residues (white) and four notable VOC mutations (orange) highlighted.

(C) Competition assay with a constant concentration of ACE2 and increasing concentrations of LCB1 using spike displayed VOCs (error bars, SD of three biological replicates as measured by flow cytometry).

Limitations

The following caveats must be kept in mind when designing experiments conducted on surface-tethered proteins. The C-terminal transmembrane domain and linker peptide may alter antibody accessibility relative to native spike proteins. Simultaneous binding of the spike antigen and FLAG epitopes may slightly alter conformations and epitope accessibility. These effects should be considered when profiling antibodies with different binding modes. The glycosylation pattern may also not match that seen in native viruses. Using pre-fusion stabilizing mutations in the base construct (6P-D614G) increases the overall expression level but may alter intra-trimer conformational changes, such as the recently reported opening of the S2 domains (Huang et al., 2021). When profiling large changes in the antigenicity of variant spikes, we compared binding at a limited set of antibody concentrations. Performing a serial dilution series with eight or more antibody concentrations will reflect changes in the apparent binding kinetics (Adams et al., 2016; Greaney et al., 2021b; Starr et al., 2020). If such information is necessary for a particular application, Spike Display can perform comprehensive titration curves. Follow-up viral neutralization assays, structural studies, and other biophysical assays should be conducted to further bolster these *in vitro* measurements.

Spike Display is not yet compatible with high-throughput pooled variant screening and DMS. Current iterations of Spike Display screen viral glycoprotein variants one at a time, making this a useful platform for rapid assessment of protein stability, epitope mapping of antibody-binding sites, and assessment of circulating variants of interest. However, we anticipate that Spike Display library sizes can be extended by several orders of magnitude with recently developed DMS tools in mammalian cells (Matreyek et al., 2020). Using pooled libraries and fluoresce-assisted cell sorting will further increase the experimental throughput of this platform.

STAR★METHODS

Detailed methods are provided in the online version of this paper and include the following:

- KEY RESOURCES TABLE
- RESOURCE AVAILABILITY
  - Lead Contact
  - Materials Availability
  - Data and Code Availability
- EXPERIMENTAL MODEL AND SUBJECT DETAILS
- METHOD DETAILS
  - Mammalian display vectors and spike protein mutants
  - Automated spike variant cloning pipeline
  - Expression and purification of neutralizing anti-spike antibodies
  - Expression and purification of chimeric ACE2-Fc
  - Expression and purification of SARS-CoV-2 spike proteins
  - HEK293T transfection
  - Flow cytometry and data analysis
  - LCB1 cloning and purification
  - LCB1 and ACE2 competition assays
  - Cell immunostaining and microscopy
  - SARS-COV-2 pseudovirus neutralization assay
  - Western blots
  - Spike Display cleavage and spike purification
  - Negative stain EM data collection and processing
  - Biolayer Interferometry
  - Computational analysis of GISAID sequence data
  - Structural analyses and data visualization
- QUANTIFICATION AND STATISTICAL ANALYSIS

SUPPLEMENTAL INFORMATION

Supplemental information can be found online at https://doi.org/10.1016/j.molcel.2021.11.024.

ACKNOWLEDGMENTS

We thank Drs. Ching-Lin Hseih, Jason S. McLellan, Jason Lavinder, and Gregory C. Ippolito for generously sharing ACE2, antibody expression constructs, and purified antibodies. We are grateful to members of the Finkelstein and Ippolito laboratories for carefully reading the manuscript. We also thank our

Center for Biomedical Research Support for their continuous work and dedication throughout the pandemic. This work was supported by the Welch Foundation (F-1808 to I.J.F.; F-1390 to K.N.D.), the Bill and Melinda Gates Foundation (INV-017592 to I.J.F.), and a Cooperative Agreement between UT Austin and ARL (W911NF-17-2-0091 to J.D.G.).

AUTHOR CONTRIBUTIONS

K.J., C.-W.C., J.D.G., and I.J.F. designed the research. K.J. performed the flow experiments. K.J., C.-W.C., C.I.T., A.A., Q.G., E.C.G., G.N., D.R.B., J.G., W.N.V., and H.-C.K. purified antibodies and other reagents. K.J., C.I.T., A.A., Q.G, J.L., and H.Z. cloned spike variants. T.S.K. and K.N.D. provided the pseudovirus neutralization data. K.J., C.-W.C., C.I.T., H.Z., and A.P.H. analyzed the data. K.J. and I.J.F. wrote the paper with editorial assistance from all co-authors.

DECLARATION OF INTERESTS

The authors declare competing financial interests. K.J., C.-W.C., H.-C.K., and I.J.F. have filed patent applications on spike-6p (HexaPro). A patent application submitted by The University of Texas Board of Regents is pending for anti-SARS-CoV-2 monoclonal antibodies described in the manuscript (W.N.V.). The authors declare that the research was conducted in the absence of any commercial or financial relationships that could be construed as a potential conflict of interest. The authors declare no competing non-financial interests.

INCLUSION AND DIVERSITY

One or more of the authors of this paper self-identifies as an underrepresented ethnic minority in science. The author list of this paper includes contributors from the location where the research was conducted who participated in the data collection, design, analysis, and/or interpretation of the work.

Received: April 30, 2021
Revised: September 16, 2021
Accepted: November 19, 2021
Published: December 16, 2021

REFERENCES

Abdelrahman, Z., Li, M., and Wang, X. (2020). Comparative Review of SARS-CoV-2, SARS-CoV, MERS-CoV, and Influenza A Respiratory Viruses. Front. Immunol. *11*, 552909. https://doi.org/10.3389/fimmu.2020.552909.

Adams, R.M., Mora, T., Walczak, A.M., and Kinney, J.B. (2016). Measuring the sequence-affinity landscape of antibodies with massively parallel titration curves. eLife *5*, e23156. https://doi.org/10.7554/eLife.23156.

Allen, J.D., Chawla, H., Samsudin, F., Zuzic, L., Shivgan, A.T., Watanabe, Y., He, W.T., Callaghan, S., Song, G., Yong, P., et al. (2021). Site-Specific Steric Control of SARS-CoV-2 Spike Glycosylation. Biochemistry *60*, 2153–2169. https://doi.org/10.1021/acs.biochem.1c00279.

Andreano, E., Piccini, G., Licastro, D., Casalino, L., Johnson, N.V., Paciello, I., Dal Monego, S., Pantano, E., Manganaro, N., Manenti, A., et al. (2021). SARS-CoV-2 escape from a highly neutralizing COVID-19 convalescent plasma. Proc. Natl. Acad. Sci. USA *118*. e2103154118. https://doi.org/10.1073/pnas.2103154118.

Baden, L.R., Sahly, H.M.E., Essink, B., Kotloff, K., Frey, S., Novak, R., Diemert, D., Spector, S.A., Rouphael, N., Creech, C.B., et al. (2020). Efficacy and Safety of the mRNA-1273 SARS-CoV-2 Vaccine. N. Engl. J. Med. *384*, 403–416.

Bloom, J.D., Gong, L.I., and Baltimore, D. (2010). Permissive secondary mutations enable the evolution of influenza oseltamivir resistance. Science *328*, 1272–1275. https://doi.org/10.1126/science.1187816.

Bobay, L.-M., O'Donnell, A.C., and Ochman, H. (2020). Recombination events are concentrated in the spike protein region of Betacoronaviruses. PLoS Genet. *16*, e1009272. https://doi.org/10.1371/journal.pgen.1009272.

Bruun, T.-H., Grassmann, V., Zimmer, B., Asbach, B., Peterhoff, D., Kliche, A., and Wagner, R. (2017). Mammalian cell surface display for monoclonal antibody-based FACS selection of viral envelope proteins. MAbs *9*, 1052–1064. https://doi.org/10.1080/19420862.2017.1364824.

Cantuti-Castelvetri, L., Ojha, R., Pedro, L.D., Djannatian, M., Franz, J., Kuivanen, S., van der Meer, F., Kallio, K., Kaya, T., Anastasina, M., et al. (2020). Neuropilin-1 facilitates SARS-CoV-2 cell entry and infectivity. Science *370*, 856–860. https://doi.org/10.1126/science.abd2985.

Cao, L., Goreshnik, I., Coventry, B., Case, J.B., Miller, L., Kozodoy, L., Chen, R.E., Carter, L., Walls, A.C., Park, Y.-J., et al. (2020). De novo design of picomolar SARS-CoV-2 miniprotein inhibitors. Science *370*, 426–431. https://doi.org/10.1126/science.abd9909.

Cerutti, G., Guo, Y., Zhou, T., Gorman, J., Lee, M., Rapp, M., Reddem, E.R., Yu, J., Bahna, F., Bimela, J., et al. (2021). Potent SARS-CoV-2 neutralizing antibodies directed against spike N-terminal domain target a single supersite. Cell Host Microbe *29*, 819–833.e7. https://doi.org/10.1016/j.chom.2021.03.005.

Chan, K.K., Dorosky, D., Sharma, P., Abbasi, S.A., Dye, J.M., Kranz, D.M., Herbert, A.S., and Procko, E. (2020). Engineering human ACE2 to optimize binding to the spike protein of SARS coronavirus 2. Science *369*, 1261–1265. https://doi.org/10.1126/science.abc0870.

Chan, K.K., Tan, T.J.C., Narayanan, K.K., and Procko, E. (2021). An engineered decoy receptor for SARS-CoV-2 broadly binds protein S sequence variants. Sci. Adv. *7*, eabf1738.

Cheung, R., Insigne, K.D., Yao, D., Burghard, C.P., Wang, J., Hsiao, Y.E., Jones, E.M., Goodman, D.B., Xiao, X., and Kosuri, S. (2019). A Multiplexed Assay for Exon Recognition Reveals that an Unappreciated Fraction of Rare Genetic Variants Cause Large-Effect Splicing Disruptions. Mol. Cell *73*, 183–194.e8. https://doi.org/10.1016/j.molcel.2018.10.037.

Chi, X., Yan, R., Zhang, J., Zhang, G., Zhang, Y., Hao, M., Zhang, Z., Fan, P., Dong, Y., Yang, Y., et al. (2020). A neutralizing human antibody binds to the N-terminal domain of the Spike protein of SARS-CoV-2. Science *369*, 650–655. https://doi.org/10.1126/science.abc6952.

Crawford, K.H.D., Eguia, R., Dingens, A.S., Loes, A.N., Malone, K.D., Wolf, C.R., Chu, H.Y., Tortorici, M.A., Veesler, D., Murphy, M., et al. (2020). Protocol and Reagents for Pseudotyping Lentiviral Particles with SARS-CoV-2 Spike Protein for Neutralization Assays. Viruses *12*, E513. https://doi.org/10.3390/v12050513.

Daily, J. (2016). Parasail: SIMD C library for global, semi-global, and local pairwise sequence alignments. BMC Bioinformatics *17*, 81. https://doi.org/10.1186/s12859-016-0930-z.

Davies, N.G., Abbott, S., Barnard, R.C., Jarvis, C.I., Kucharski, A.J., Munday, J.D., Pearson, C.A.B., Russell, T.W., Tully, D.C., Washburne, A.D., et al.; CMMID COVID-19 Working Group; COVID-19 Genomics UK (COG-UK) Consortium (2021). Estimated transmissibility and impact of SARS-CoV-2 lineage B.1.1.7 in England. Science *372*, eabg3055.

Deng, X., Garcia-Knight, M.A., Khalid, M.M., Servellita, V., Wang, C., Morris, M.K., Sotomayor-González, A., Glasner, D.R., Reyes, K.R., Gliwa, A.S., et al. (2021). Transmission, infectivity, and antibody neutralization of an emerging SARS-CoV-2 variant in California carrying a L452R spike protein mutation. medRxiv, 2021.03.07.21252647.

Elbe, S., and Buckland-Merrett, G. (2017). Data, disease and diplomacy: GISAID's innovative contribution to global health. Glob Chall *1*, 33–46. https://doi.org/10.1002/gch2.1018.

Friedrich, T.C., Frye, C.A., Yant, L.J., O'Connor, D.H., Kriewaldt, N.A., Benson, M., Vojnov, L., Dodds, E.J., Cullen, C., Rudersdorf, R., et al. (2004). Extraepitopic compensatory substitutions partially restore fitness to simian immunodeficiency virus variants that escape from an immunodominant cytotoxic-T-lymphocyte response. J. Virol. *78*, 2581–2585. https://doi.org/10.1128/JVI.78.5.2581-2585.2004.

Garcia-Beltran, W.F., Lam, E.C., St Denis, K., Nitido, A.D., Garcia, Z.H., Hauser, B.M., Feldman, J., Pavlovic, M.N., Gregory, D.J., Poznansky, M.C., et al. (2021). Multiple SARS-CoV-2 variants escape neutralization by

vaccine-induced humoral immunity. Cell *184*, 2372–2383.e9. https://doi.org/10.1016/j.cell.2021.03.013.

Grange, Z.L., Goldstein, T., Johnson, C.K., Anthony, S., Gilardi, K., Daszak, P., Olival, K.J., O'Rourke, T., Murray, S., Olson, S.H., et al.; Expert Panel; PREDICT Consortium; University of Edinburgh Epigroup members those who wish to remain anonymous (2021). Ranking the risk of animal-to-human spillover for newly discovered viruses. Proc. Natl. Acad. Sci. USA *118*. e2002324118. https://doi.org/10.1073/pnas.2002324118.

Grant, T., Rohou, A., and Grigorieff, N. (2018). *cis*TEM, user-friendly software for single-particle image processing. eLife *7*, e35383. https://doi.org/10.7554/eLife.35383.

Grant, O.C., Montgomery, D., Ito, K., and Woods, R.J. (2020). Analysis of the SARS-CoV-2 spike protein glycan shield reveals implications for immune recognition. Sci. Rep. *10*, 14991. https://doi.org/10.1038/s41598-020-71748-7.

Greaney, A.J., Starr, T.N., Barnes, C.O., Weisblum, Y., Schmidt, F., Caskey, M., Gaebler, C., Cho, A., Agudelo, M., Finkin, S., et al. (2021a). Mapping mutations to the SARS-CoV-2 RBD that escape binding by different classes of antibodies. Nat. Commun. *12*, 4196. https://doi.org/10.1038/s41467-021-24435-8.

Greaney, A.J., Starr, T.N., Gilchuk, P., Zost, S.J., Binshtein, E., Loes, A.N., Hilton, S.K., Huddleston, J., Eguia, R., Crawford, K.H.D., et al. (2021b). Complete Mapping of Mutations to the SARS-CoV-2 Spike Receptor-Binding Domain that Escape Antibody Recognition. Cell Host Microbe *29*, 44–57.e9. https://doi.org/10.1016/j.chom.2020.11.007.

Hamilton, S.R., Bobrowicz, P., Bobrowicz, B., Davidson, R.C., Li, H., Mitchell, T., Nett, J.H., Rausch, S., Stadheim, T.A., Wischnewski, H., et al. (2003). Production of complex human glycoproteins in yeast. Science *301*, 1244–1246. https://doi.org/10.1126/science.1088166.

Hansen, J., Baum, A., Pascal, K.E., Russo, V., Giordano, S., Wloga, E., Fulton, B.O., Yan, Y., Koon, K., Patel, K., et al. (2020). Studies in humanized mice and convalescent humans yield a SARS-CoV-2 antibody cocktail. Science *369*, 1010–1014. https://doi.org/10.1126/science.abd0827.

Hoffmann, M., Arora, P., Groß, R., Seidel, A., Hörnich, B.F., Hahn, A.S., Krüger, N., Graichen, L., Hofmann-Winkler, H., Kempf, A., et al. (2021). SARS-CoV-2 variants B.1.351 and P.1 escape from neutralizing antibodies. Cell *184*, 2384–2393.e12. https://doi.org/10.1016/j.cell.2021.03.036.

Hsieh, C.-L., Goldsmith, J.A., Schaub, J.M., DiVenere, A.M., Kuo, H.-C., Javanmardi, K., Le, K.C., Wrapp, D., Lee, A.G., Liu, Y., et al. (2020). Structure-based design of prefusion-stabilized SARS-CoV-2 spikes. Science *369*, 1501–1505. https://doi.org/10.1126/science.abd0826.

Huang, Y., Nguyen, A.W., Hsieh, C.-L., Silva, R., Olaluwoye, O.S., Wilen, R.E., Kaoud, T.S., Azouz, L.R., Qerqez, A.N., Le, K.C., Bohanon, A.L., et al. (2021). Identification of a conserved neutralizing epitope present on spike proteins from all highly pathogenic coronaviruses. bioRxiv, https://doi.org/10.1101/2021.01.31.428824.

Juraszek, J., Rutten, L., Blokland, S., Bouchier, P., Voorzaat, R., Ritschel, T., Bakkers, M.J.G., Renault, L.L.R., and Langedijk, J.P.M. (2021). Stabilizing the closed SARS-CoV-2 spike trimer. Nature Communications *12*, 244.

Kirchdoerfer, R.N., Cottrell, C.A., Wang, N., Pallesen, J., Yassine, H.M., Turner, H.L., Corbett, K.S., Graham, B.S., McLellan, J.S., and Ward, A.B. (2016). Pre-fusion structure of a human coronavirus spike protein. Nature *531*, 118–121. https://doi.org/10.1038/nature17200.

Korber, B., Fischer, W.M., Gnanakaran, S., Yoon, H., Theiler, J., Abfalterer, W., Hengartner, N., Giorgi, E.E., Bhattacharya, T., Foley, B., et al.; Sheffield COVID-19 Genomics Group (2020). Tracking Changes in SARS-CoV-2 Spike: Evidence that D614G Increases Infectivity of the COVID-19 Virus. Cell *182*, 812–827.e19. https://doi.org/10.1016/j.cell.2020.06.043.

Kreuder Johnson, C., Hitchens, P.L., Smiley Evans, T., Goldstein, T., Thomas, K., Clements, A., Joly, D.O., Wolfe, N.D., Daszak, P., Karesh, W.B., and Mazet, J.K. (2015). Spillover and pandemic properties of zoonotic viruses with high host plasticity. Sci. Rep. *5*, 14830. https://doi.org/10.1038/srep14830.

Lavinder, J.J., Horton, A.P., Georgiou, G., and Ippolito, G.C. (2015). Next-generation sequencing and protein mass spectrometry for the comprehensive analysis of human cellular and serum antibody repertoires. Curr. Opin. Chem. Biol. *24*, 112–120. https://doi.org/10.1016/j.cbpa.2014.11.007.

Lee, M.E., DeLoache, W.C., Cervantes, B., and Dueber, J.E. (2015). A Highly Characterized Yeast Toolkit for Modular, Multipart Assembly. ACS Synth. Biol. *4*, 975–986. https://doi.org/10.1021/sb500366v.

Leung, K., Shum, M.H., Leung, G.M., Lam, T.T., and Wu, J.T. (2021). Early transmissibility assessment of the N501Y mutant strains of SARS-CoV-2 in the United Kingdom, October to November 2020. Euro Surveill. *26*, 2002106. https://doi.org/10.2807/1560-7917.ES.2020.26.1.2002106.

Li, F. (2016). Structure, Function, and Evolution of Coronavirus Spike Proteins. Annu. Rev. Virol. *3*, 237–261. https://doi.org/10.1146/annurev-virology-110615-042301.

Li, X., Giorgi, E.E., Marichannegowda, M.H., Foley, B., Xiao, C., Kong, X.-P., Chen, Y., Gnanakaran, S., Korber, B., and Gao, F. (2020). Emergence of SARS-CoV-2 through recombination and strong purifying selection. Sci. Adv. *6*, eabb9153.

Lim, K.H., Huang, H., Pralle, A., and Park, S. (2013). Stable, high-affinity streptavidin monomer for protein labeling and monovalent biotin detection. Biotechnol. Bioeng. *110*, 57–67. https://doi.org/10.1002/bit.24605.

Linsky, T.W., Vergara, R., Codina, N., Nelson, J.W., Walker, M.J., Su, W., Barnes, C.O., Hsiang, T.-Y., Esser-Nobis, K., Yu, K., et al. (2020). De novo design of potent and resilient hACE2 decoys to neutralize SARS-CoV-2. Science *370*, 1208–1214. https://doi.org/10.1126/science.abe0075.

Liu, L., Wang, P., Nair, M.S., Yu, J., Rapp, M., Wang, Q., Luo, Y., Chan, J.F.-W., Sahi, V., Figueroa, A., et al. (2020a). Potent neutralizing antibodies against multiple epitopes on SARS-CoV-2 spike. Nature *584*, 450–456. https://doi.org/10.1038/s41586-020-2571-7.

Liu, Y., Soh, W.T., Tada, A., Arakawa, A., Matsuoka, S., Nakayama, E.E., Li, S., Ono, C., Torii, S., Kishida, K., et al. (2020b). An infectivity-enhancing site on the SARS-CoV-2 spike protein is targeted by COVID-19 patient antibodies. bioRxiv, https://doi.org/10.1101/2020.12.18.423358.

Long, S.W., Olsen, R.J., Christensen, P.A., Bernard, D.W., Davis, J.J., Shukla, M., Nguyen, M., Saavedra, M.O., Yerramilli, P., Pruitt, L., et al. (2020). Molecular Architecture of Early Dissemination and Massive Second Wave of the SARS-CoV-2 Virus in a Major Metropolitan Area. MBio *11*, e02707-20. https://doi.org/10.1128/mBio.02707-20.

Matreyek, K.A., Starita, L.M., Stephany, J.J., Martin, B., Chiasson, M.A., Gray, V.E., Kircher, M., Khechaduri, A., Dines, J.N., Hause, R.J., et al. (2018). Multiplex assessment of protein variant abundance by massively parallel sequencing. Nat. Genet. *50*, 874–882. https://doi.org/10.1038/s41588-018-0122-z.

Matreyek, K.A., Stephany, J.J., Chiasson, M.A., Hasle, N., and Fowler, D.M. (2020). An improved platform for functional assessment of large protein libraries in mammalian cells. Nucleic Acids Res. *48*, e1. https://doi.org/10.1093/nar/gkz910.

McCallum, M., Bassi, J., De Marco, A., Chen, A., Walls, A.C., Di Iulio, J., Tortorici, M.A., Navarro, M.-J., Silacci-Fregni, C., Saliba, C., et al. (2021a). SARS-CoV-2 immune evasion by the B.1.427/B.1.429 variant of concern. Science *373*, 648–654. https://doi.org/10.1126/science.abi7994.

McCallum, M., De Marco, A., Lempp, F.A., Tortorici, M.A., Pinto, D., Walls, A.C., Beltramello, M., Chen, A., Liu, Z., Zatta, F., et al. (2021b). N-terminal domain antigenic mapping reveals a site of vulnerability for SARS-CoV-2. Cell *184*, 2332–2347.e16. https://doi.org/10.1016/j.cell.2021.03.028.

McCarthy, K.R., Rennick, L.J., Nambulli, S., Robinson-McCarthy, L.R., Bain, W.G., Haidar, G., and Duprex, W.P. (2021). Recurrent deletions in the SARS-CoV-2 spike glycoprotein drive antibody escape. Science *371*, 1139–1142. https://doi.org/10.1126/science.abf6950.

McMahan, K., Yu, J., Mercado, N.B., Loos, C., Tostanoski, L.H., Chandrashekar, A., Liu, J., Peter, L., Atyeo, C., Zhu, A., et al. (2021). Correlates of protection against SARS-CoV-2 in rhesus macaques. Nature *590*, 630–634. https://doi.org/10.1038/s41586-020-03041-6.

Pallesen, J., Wang, N., Corbett, K.S., Wrapp, D., Kirchdoerfer, R.N., Turner, H.L., Cottrell, C.A., Becker, M.M., Wang, L., Shi, W., et al. (2017). Immunogenicity and structures of a rationally designed prefusion MERS-CoV spike antigen. Proc. Natl. Acad. Sci. USA *114*, E7348–E7357. https://doi.org/10.1073/pnas.1707304114.

Peiris, J.S.M., Yuen, K.Y., Osterhaus, A.D.M.E., and Stöhr, K. (2003). The severe acute respiratory syndrome. N. Engl. J. Med. *349*, 2431–2441. https://doi.org/10.1056/NEJMra032498.

Piccoli, L., Park, Y.-J., Tortorici, M.A., Czudnochowski, N., Walls, A.C., Beltramello, M., Silacci-Fregni, C., Pinto, D., Rosen, L.E., Bowen, J.E., et al. (2020). Mapping Neutralizing and Immunodominant Sites on the SARS-CoV-2 Spike Receptor-Binding Domain by Structure-Guided High-Resolution Serology. Cell *183*, 1024–1042.e21. https://doi.org/10.1016/j.cell.2020.09.037.

Planas, D., Veyer, D., Baidaliuk, A., Staropoli, I., Guivel-Benhassine, F., Rajah, M.M., Planchais, C., Porrot, F., Robillard, N., Puech, J., et al. (2021). Reduced sensitivity of SARS-CoV-2 variant Delta to antibody neutralization. Nature *596*, 276–280. https://doi.org/10.1038/s41586-021-03777-9.

Polack, F.P., Thomas, S.J., Kitchin, N., Absalon, J., Gurtman, A., Lockhart, S., Perez, J.L., Pérez Marc, G., Moreira, E.D., Zerbini, C., et al.; C4591001 Clinical Trial Group (2020). Safety and Efficacy of the BNT162b2 mRNA Covid-19 Vaccine. N. Engl. J. Med. *383*, 2603–2615. https://doi.org/10.1056/NEJMoa2034577.

Punjani, A., Rubinstein, J.L., Fleet, D.J., and Brubaker, M.A. (2017). cryoSPARC: algorithms for rapid unsupervised cryo-EM structure determination. Nat. Methods *14*, 290–296. https://doi.org/10.1038/nmeth.4169.

Shelton, S.B., Shah, N.M., Abell, N.S., Devanathan, S.K., Mercado, M., and Xhemalçe, B. (2018). Crosstalk between the RNA Methylation and Histone-Binding Activities of MePCE Regulates P-TEFb Activation on Chromatin. Cell Rep. *22*, 1374–1383. https://doi.org/10.1016/j.celrep.2018.01.028.

Starr, T.N., Greaney, A.J., Hilton, S.K., Ellis, D., Crawford, K.H.D., Dingens, A.S., Navarro, M.J., Bowen, J.E., Tortorici, M.A., Walls, A.C., et al. (2020). Deep Mutational Scanning of SARS-CoV-2 Receptor Binding Domain Reveals Constraints on Folding and ACE2 Binding. Cell *182*, 1295–1310.e20. https://doi.org/10.1016/j.cell.2020.08.012.

Starr, T.N., Greaney, A.J., Addetia, A., Hannon, W.W., Choudhary, M.C., Dingens, A.S., Li, J.Z., and Bloom, J.D. (2021). Prospective mapping of viral mutations that escape antibodies used to treat COVID-19. Science *371*, 850–854. https://doi.org/10.1126/science.abf9302.

Suryadevara, N., Shrihari, S., Gilchuk, P., VanBlargan, L.A., Binshtein, E., Zost, S.J., Nargi, R.S., Sutton, R.E., Winkler, E.S., Chen, E.C., et al. (2021). Neutralizing and protective human monoclonal antibodies recognizing the N-terminal domain of the SARS-CoV-2 spike protein. Cell *184*, 2316–2331.e15. https://doi.org/10.1016/j.cell.2021.03.029.

Tai, W., He, L., Zhang, X., Pu, J., Voronin, D., Jiang, S., Zhou, Y., and Du, L. (2020). Characterization of the receptor-binding domain (RBD) of 2019 novel coronavirus: implication for development of RBD protein as a viral attachment inhibitor and vaccine. Cell. Mol. Immunol. *17*, 613–620. https://doi.org/10.1038/s41423-020-0400-4.

Urdaniz, I.F., Steiner, P.J., Kirby, M.B., Zhao, F., Haas, C.M., Barman, S., Rhodes, E.R., Peng, L., Sprenger, K.G., Jardine, J.G., and Whitehead, T.A. (2021). One-shot identification of SARS-CoV-2 S RBD escape mutants using yeast screening. bioRxiv. 2021.03.15.435309. https://doi.org/10.1101/2021.03.15.435309.

Voss, W.N., Hou, Y.J., Johnson, N.V., Delidakis, G., Kim, J.E., Javanmardi, K., Horton, A.P., Bartzoka, F., Paresi, C.J., Tanno, Y., et al. (2021). Prevalent, protective, and convergent IgG recognition of SARS-CoV-2 non-RBD spike epitopes. Science *372*, 1108–1112. https://doi.org/10.1126/science.abg5268.

Wagner, E.K., Qerqez, A.N., Stevens, C.A., Nguyen, A.W., Delidakis, G., and Maynard, J.A. (2019). Human cytomegalovirus-specific T-cell receptor engineered for high affinity and soluble expression using mammalian cell display. J. Biol. Chem. *294*, 5790–5804. https://doi.org/10.1074/jbc.RA118.007187.

Wang, P., Nair, M.S., Liu, L., Iketani, S., Luo, Y., Guo, Y., Wang, M., Yu, J., Zhang, B., Kwong, P.D., et al. (2021). Antibody resistance of SARS-CoV-2 variants B.1.351 and B.1.1.7. Nature *593*, 130–135. https://doi.org/10.1038/s41586-021-03398-2.

Wickham, H., Averick, M., Bryan, J., Chang, W., McGowan, L.D., François, R., Grolemund, G., Hayes, A., Henry, L., Hester, J., et al. (2019). Welcome to the Tidyverse. Journal of Open Source Software *4*, 1686. https://doi.org/10.21105/joss.01686.

Woo, P.C.Y., Lau, S.K.P., Chu, C.M., Chan, K.H., Tsoi, H.W., Huang, Y., Wong, B.H.L., Poon, R.W.S., Cai, J.J., Luk, W.K., et al. (2005). Characterization and complete genome sequence of a novel coronavirus, coronavirus HKU1, from patients with pneumonia. J. Virol. *79*, 884–895. https://doi.org/10.1128/JVI.79.2.884-895.2005.

Wrapp, D., Wang, N., Corbett, K.S., Goldsmith, J.A., Hsieh, C.-L., Abiona, O., Graham, B.S., and McLellan, J.S. (2020). Cryo-EM structure of the 2019-nCoV spike in the prefusion conformation. Science *367*, 1260–1263. https://doi.org/10.1126/science.abb2507.

Wu, N.C., Xie, J., Zheng, T., Nycholat, C.M., Grande, G., Paulson, J.C., Lerner, R.A., and Wilson, I.A. (2017). Diversity of Functionally Permissive Sequences in the Receptor-Binding Site of Influenza Hemagglutinin. Cell Host Microbe *21*, 742–753.e8. https://doi.org/10.1016/j.chom.2017.05.011.

Xie, X., Liu, Y., Liu, J., Zhang, X., Zou, J., Fontes-Garfias, C.R., Xia, H., Swanson, K.A., Cutler, M., Cooper, D., et al. (2021). Neutralization of SARS-CoV-2 spike 69/70 deletion, E484K and N501Y variants by BNT162b2 vaccine-elicited sera. Nat. Med. *27*, 620–621. https://doi.org/10.1038/s41591-021-01270-4.

Yan, R., Zhang, Y., Li, Y., Xia, L., Guo, Y., and Zhou, Q. (2020). Structural basis for the recognition of SARS-CoV-2 by full-length human ACE2. Science *367*, 1444–1448. https://doi.org/10.1126/science.abb2762.

Yao, H., Song, Y., Chen, Y., Wu, N., Xu, J., Sun, C., Zhang, J., Weng, T., Zhang, Z., Wu, Z., et al. (2020). Molecular Architecture of the SARS-CoV-2 Virus. Cell *183*, 730–738.e13. https://doi.org/10.1016/j.cell.2020.09.018.

Yu, J., Tostanoski, L.H., Peter, L., Mercado, N.B., McMahan, K., Mahrokhian, S.H., Nkolola, J.P., Liu, J., Li, Z., Chandrashekar, A., et al. (2020). DNA vaccine protection against SARS-CoV-2 in rhesus macaques. Science *369*, 806–811. https://doi.org/10.1126/science.abc6284.

Yurkovetskiy, L., Wang, X., Pascal, K.E., Tomkins-Tinch, C., Nyalile, T.P., Wang, Y., Baum, A., Diehl, W.E., Dauphin, A., Carbone, C., et al. (2020). Structural and Functional Analysis of the D614G SARS-CoV-2 Spike Protein Variant. Cell *183*, 739–751.e8. https://doi.org/10.1016/j.cell.2020.09.032.

Zhang, H., Penninger, J.M., Li, Y., Zhong, N., and Slutsky, A.S. (2020). Angiotensin-converting enzyme 2 (ACE2) as a SARS-CoV-2 receptor: molecular mechanisms and potential therapeutic target. Intensive Care Med. *46*, 586–590. https://doi.org/10.1007/s00134-020-05985-9.

Zhang, C., Wang, Y., Zhu, Y., Liu, C., Gu, C., Xu, S., Wang, Y., Zhou, Y., Wang, Y., Han, W., et al. (2021). Development and structural basis of a two-MAb cocktail for treating SARS-CoV-2 infections. Nat. Commun. *12*, 264. https://doi.org/10.1038/s41467-020-20465-w.

Zhou, C., Jacobsen, F.W., Cai, L., Chen, Q., and Shen, W.D. (2010). Development of a novel mammalian cell surface antibody display platform. MAbs *2*, 508–518. https://doi.org/10.4161/mabs.2.5.12970.

Zumla, A., Hui, D.S., and Perlman, S. (2015). Middle East respiratory syndrome. Lancet *386*, 995–1007. https://doi.org/10.1016/S0140-6736(15)60454-8.

STAR★METHODS

KEY RESOURCES TABLE

REAGENT or RESOURCE	SOURCE	IDENTIFIER
Antibodies		
Mouse anti-FLAG M2	Sigma-Aldrich	F3165
Goat anti-Mouse IgG(H+L), Human ads-Alexa Fluor 488	SouthernBiotech	1031-30
Goat anti-Human IgG Fc-Alexa Fluor 647	SouthernBiotech	2048-31
Goat anti-Mouse IgG H&L (IRDYe 680RD)	abcam	ab216776
Beta Tubulin Loading Control Monoclonal Antibody (BT7R)	Thermo Fisher	MA5-16308
Bacterial strains		
NEB 5-alpha Competent *E. coli* (High Efficiency)	NEB	C2987H
Mix & Go Competent Cells - Strain Zymo 10B	Zymo Research	T3019
Rosetta (DE3) Competent Cells	Sigma-Aldrich	70954
Chemicals, peptides, and recombinant proteins		
Superior Broth	AthenaES	0105
Expi293 Expression Medium	Sigma-Aldrich	A1435101
DMEM, high glucose, pyruvate	GIBCO	11995065
Fetal Bovine Serum	GIBCO	26140079
Opti-MEM I Reduced Serum Medium, GlutaMAX Supplement	GIBCO	51985091
Penicillin-Streptomycin (5,000 U/mL)	Thermo Fisher	15070063
Lipofectamine 3000 Transfection Reagent	Thermo Fisher	L3000015
Bovine Serum Albumin	Sigma-Aldrich	A3294
EZCut TEV Protease, Recombinant	BioVision	7847-100
ACE2 Fc Chimera, Human	Genscript	Z03484
Hoechst stain	Thermo Fisher	62249
Odyssey Blocking Buffer (PBS)	LI-COR	927-40000
Surfactant P20	Cytiva	BR100054
cOmplete, EDTA-free Protease Inhibitor Cocktail	Sigma-Aldrich	11873580001
Nano-W (Methylamine Tungstate)	Nanoprobes	#2018
Commercial kits and enzymes		
NEBuilder HiFi DNA Assembly Master Mix	NEB	E2621S
Esp3I	NEB	R0734
T7 DNA Ligase	NEB	M0318S
AarI (2 U/μL)	Thermo Fisher	ER1582
T4 DNA Ligase	NEB	B0202S
Promega Wizard SV 96 Plasmid DNA Purification Kit	Promega	A2250
Expi293 Transfection kit	Sigma-Aldrich	L3287
Mycoplasma Detection Kit	SouthernBiotech	13100-01
Pierce HRV 3C Protease Solution Kit	Thermo Fisher	88946
Bright-Glo Luciferase Assay System	Promega	E2610

(*Continued on next page*)

Continued		
REAGENT or RESOURCE	SOURCE	IDENTIFIER
qPCR Lentivirus Titer Kit	Applied Biological Materials (abm)	LV900
Deposited data		
Raw images	This paper; Mendeley Data	https://doi.org/10.17632/k2kbt5gs6y.1
Spike Display data	This paper	Data S1
Experimental models: Cell lines		
HEK293T	ATCC	CRL-3216
Expi293F Cells	Thermo Fisher	A14527
Oligonucleotides		
Synthetic DNA sequences for cloning, see Table S3	Integrated DNA Technlologies	N/A
Recombinant DNA		
SARS-CoV-2 S HexaPro	Hsieh et al., 2020	Addgene #154754
pcDNA5/FRT/TO/intron/GFP	Shelton et al., 2018	Addgene #113547
pYTK001	Lee et al., 2015	Addgene #65108
HDM-Hgpm2	BEI Resources	NR-52516
pRC-CMV-Rev1b	BEI Resources	NR-52519
HDM-tat1b	BEI Resources	NR-5251
Vector pHAGE2 Containing the ZsGreen Gene	BEI Resources	NR-52520
pCMV-VSV-G Envelope Vector	Cell Biolabs	RV-110
Software and algorithms		
FlowJo v9	BD	N/A
NIS-Elements D	Nikon	MQS33000
cryoSPARC v3.1.0	cryoSPARC	N/A
CisTEM	https://cistem.org/software	N/A
SA3800 Spectral Cell Analyzer Software	SONY	N/A
R Tidyverse	https://www.tidyverse.org/	N/A
ggplot2 (v3.3.5)	https://ggplot2.tidyverse.org/	N/A
ChimeraX 1.1	UCSF	N/A
GraphPad Prism	GraphPad	N/A
Octet Data Analysis software (v11.1)	FortéBio	N/A
Public datasets		
Expression summary of variants with single substitutions	Hsieh et al., 2020	Table S1
Yeast display RBD expression, ACE2 binding, and mAb escape data	Starr et al., 2020, 2021	https://jbloomlab.github.io/SARS-CoV-2-RBD_MAP_clinical_Abs/
Other		
384-well Echo Source Plate	Biolab	PP-0200
96-well Clear Round Bottom 2.2 mL Polypropylene Deep Well Plate (Sterile)	Axygen	P-2ML-SQ-C-S
Nunc OmniTray Single-Well Plate	Thermo Fisher	140156
Protein G magnetic beads	Promega	G7471
Protein A Agarose	Thermo Fisher	15918014
Strep-Tactin Superflow resin	IBA	2-1206-025

(*Continued on next page*)

Continued

REAGENT or RESOURCE	SOURCE	IDENTIFIER
Superose 6 increase 10/300	GE healthcare	GE29-0915-96
Cell Culture Dishes (10 cm)	VWR	10062-880
Cell Culture Plate (6-well)	VWR	10861-696
Cell Culture Plate (12-well)	VWR	10861-698
LUNA-II Automated Cell Counter	Logos biosystems	L40002
SA3800 Spectral Analyzer	SONY	N/A
5 mL HisTrap HP column	GE healthcare	17524802
Cell Imaging Dish, 35 × 10 mm	Eppendorf	0030740009
Ts2R-FL inverted research microscope	Nikon	MFA51010
CF160 Plan Apochromat Lambda 60x oil immersion objective	Nikon	MRD01605
C-FL AT DAPI/HOECHST/ALEXA FLUOR 350; Filter Cube	Chroma	96221
C-FL AT EGFP/FITC/CY2/ALEXA FLUOR 488; Filter Cube	Chroma	96226
C-FL AT TEXAS RED/mCHERY/ALEXA FLUOR 594; Filter Cube	Chroma	96231
SOLA SM II 365 LIGHT ENGINE	Lumencor	77060075
PCO.PANDA USB3.1 SCMOS CAMERA	PCO	77067012
Mini-PROTEAN TGX Precast Protein Gel	Bio-Rad	4561084
Trans-Blot SD. Semi-Dry Electrophoretic Transfer Cell	Bio-Rad	1703940
EM Grids	Electron Microscopy Sciences	CF400-Cu
Talos F200C TEM microscope	Thermo Fisher	N/A
Ceta 16M detector	Thermo Fisher	N/A
Octet Anti-Mouse Fc Capture (AMC) Biosensors	FortéBio	18-5088
Octet RED96e	FortéBio	N/A
IncuCyte ZOOM	Essen BioScience	N/A
QPix 420	Molecular Devices	N/A
Detailed bench protocols for Spike Display assays	This paper	Methods S1

RESOURCE AVAILABILITY

Lead Contact

- Further information and reasonable requests for resources and reagents should be directed to and will be fulfilled by the lead contact, Ilya Finkelstein (ilya@finkelsteinlab.org).

Materials Availability

- Plasmids generated in this study have been deposited to Addgene (Addgene ID: 172721-172738).
- This study did not generate new cell lines or antibodies.

Data and Code Availability

- Original western blot and microscopy images have been deposited at Mendeley and are publicly available as of the date of publication (https://doi.org/10.17632/k2kbt5gs6y.1).
- Additional Supplementary Items are available from Mendeley Data at https://doi.org/10.17632/k2kbt5gs6y.1.
- This paper analyzes existing, publicly available data from previous publications. Datasets are listed in the key resources table.
- This paper does not report original code.
- Any additional information required to reanalyze the data reported in this paper is available from the lead contact upon reasonable request.

EXPERIMENTAL MODEL AND SUBJECT DETAILS

HEK293T cells were cultured in DMEM (GIBCO 11995065) containing phenol red, 4 mM L-glutamine, 110 mg L^{-1} sodium pyruvate, 4.5 g L^{-1} D-glucose, and supplemented with 10% FBS (GIBCO 26140079) and 2% Pen/Strep (Thermo Fisher 15070063). Cells lines were tested for mycoplasma contamination before use via the Mycoplasma Detection Kit (SouthernBiotech 13100-01). Cells were maintained in a humidified atmosphere of 5% CO_2 and 37°C and were passaged every 2-3 days into 10 cm polystyrene coated plates (VWR 10062-880) upon reaching high density.

METHOD DETAILS

Mammalian display vectors and spike protein mutants

Spike Display plasmids incorporate the pre-fusion stabilized SARS-CoV-2 S-6P (“HexaPro”) as the reference sequence for all spike variants (Addgene #154754) (Hsieh et al., 2020). This construct comprises residues 1-1208 of SARS-CoV-2 S gene (GenBank MN908947) with prolines substituted at residues F817, A892, A899, A942, K986, V987, a protease-inactive furin cleavage site (^{682}GSAS^{685}), and the globally dominant D614G mutation (Korber et al., 2020). This construct (6P-D614G) was optimized for mammalian cell surface display and high-throughput Golden Gate cloning as described below.

For mammalian surface display, spike was cloned into a pcDNA5-based vector (Addgene #113547) with the addition of an N-terminal Ig-Kappa leader and C-terminal linker (3xFLAG, Strep Tag II epitope, HRV 3C protease site, and a PDGFR-β transmembrane domain) ordered as two separate gBlocks (IDT) (Table S3). All three parts (N-terminal leader, spike, and C-terminal additions) were assembled with the pcDNA5 backbone using Hi-Fi DNA assembly (NEB E2621S).

To enable high-throughput Golden Gate cloning of cell surface-displayed spike variants, the spike coding region was divided into 5 parts with junctions strategically positioned at amino acids with low mutational frequencies, according to the GISAID. For each of the 5 parts, an entry vector was constructed by cloning in a superfolder GFP (sfGFP) bacterial expression cassette with flanking AarI cut sites and unique 4 nt overhangs matching the WT SARS-CoV-2 sequence of each part junction, using PCR and Hi-Fi DNA assembly (Methods S1). A part 1-5 entry vector was made with the entire SARS-CoV-2 coding sequence replaced with the sfGFP cassette to enable multi-part assemblies of complex spike variants or entirely different spike proteins.

Parts 1,2,3,4, and 5 of the 6P-D614G coding sequence were PCR amplified with flanking AarI cut sites, the matching 4 nt overhangs for spike assembly (Methods S1), Esp3I cut sites, and 4 nt overhangs for YTK001 assembly. The Esp3I (NEB R0734) and adjacent 4 nt overhangs were used to incorporate, via Golden Gate cloning, each part (1-5) coding sequences into the YTK001 (Addgene #65108) entry vector from the yeast toolkit (Lee et al., 2015). All plasmids were verified by Sanger sequencing.

Automated spike variant cloning pipeline

Golden Gate constructs were assembled using a high-throughput automated pipeline that includes an Echo 525 Acoustic Liquid Handler, a Tecan Fluent, and a QPix 420 Colony Picker.

Golden Gate compatible parts were arranged in a 384-well Echo Source Plate (PP-0200) and transferred to 96-well PCR destination plates using the Echo 525. Each well of the 96-well destination plate received the following Golden Gate reaction mixture: 0.25 μL of T7 DNA Ligase (NEB M0318S), 0.25 μL of AarI (Thermo Fisher ER1582), 0.2 μL AarI Oligo (Thermo Fisher), 1 μL T4 DNA Ligase Buffer (NEB B0202A), 1 μL of the insert and plasmid DNA, and nuclease-free water to bring the final volume to 10 μL per reaction.

Reaction mixtures were incubated on a thermocycler according to the following conditions: 25 cycles of digestion and ligation (37°C for 1 min, and 16°C for 2 min), followed by a final digestion (37°C for 30 min), and a heat inactivation step (80°C for 20 min). To improve assembly efficiencies for assemblies with 4+ parts increase the cycled digestion and ligation steps to 3 and 5 min, respectively.

96-well PCR plates with 50 μL of Zymo DH10β Mix & Go Competent Cells (prepared using Zymo T3019) in each well were prepared beforehand so that high-throughput transfers could be done using multichannel pipettes or the Tecan Fluent. To transform the cells, we transferred 4 μL from each unique reaction mixture to respective wells containing 50 μL of Mix & Go Competent Cells. Wells were mixed by pipetting and the cells were incubated at 4°C for 10 min. The DNA-cell mixtures were then transferred to an Axygen deep well grow block (P-2ML-SQ-C-S) with 150 μL of superior broth (AthenaES 0105) in each well and incubated for 1 h at 37°C while shaking at 950 rpm on a plate shaker.

Outgrown cells were plated dropwise on Nunc OmniTrays (5 μL per spot) (Thermo Fisher 140156), containing LB-agar + carbenicillin at 100 μg mL^{-1}. Each plate can fit 96, 5 μL drops. Plates were kept at room temperature until the drops dried and were then transferred to a 37°C incubator for growth overnight (12–16 h).

Colonies were screened the following day and picked using the QPix 420 (Molecular Devices), selecting only white colonies and avoiding green fluorescent colonies, which still contain the sfGFP cassette and not the desired spike sequence. Colonies were picked into 1 mL of SB media with Carbenicillin in Axygen deep well grow blocks and grown overnight at 37°C while shaking. Once grown, liquid cultures were pelleted at 3000 g for 10 min and miniprepped using the Tecan Fluent Robotic Liquid Handler with Promega Wizard SV 96 Plasmid DNA Purification Kit (Promega A2250). All plasmids were verified using Sanger sequencing.

Expression and purification of neutralizing anti-spike antibodies

Previously published VH and VL sequences were purchased as gBlocks (IDT) to create full-length antibody IgGs (Chi et al., 2020; Hansen et al., 2020; Liu et al., 2020a). VHs and VLs were cloned into custom Golden Gate compatible pcDNA3.4 vectors for IgG1 expression.

Expi293 cells were cultured in Expi293 Expression Medium (Sigma-Aldrich A1435101) and maintained in a humidified atmosphere of 8% CO_2 and 37°C while shaking continuously at 125 rpm. Cells were transfected with a 1:3 molar ratio of VH and VL expression vectors using the Expi293 Transfection kit (Sigma-Aldrich L3287) according to the manufacturer's instructions. Five days after transfection, the protein-containing supernatant was collected by two centrifugation steps. Cells and supernatant were first separated by spinning cultures at 300 g for 5 min at 4°C. Cell debris and supernatant were separated by spinning at 3,000 g for 25 min at 4°C. To purify human IgGs, Protein G magnetic beads (Promega G7471) were washed with PBS buffer and then added to the supernatant in a 1:10 volumetric ratio. After incubating supernatant and bead mixtures for 1 h with gyration at room temperature, bead-bound antibodies were pelleted on a magnetic peg stand for washing and final elution with 100 mM glycine-HCl pH 2.5. Residual beads were clarified by running the elute through a 0.22 μm syringe filter, and then neutralized with 2 M Tris buffer pH 7.5. Purified antibodies were kept at 4°C for short-term storage, and frozen at −20°C for long-term storage.

Expression and purification of chimeric ACE2-Fc

Human ACE2-Fc was recombinantly expressed in Expi293 cells with minor modifications to previously published protocols (Wrapp et al., 2020). Simply, the ACE2-Fc expression vector was transfected into Expi293T cells according to the manufacturer's instructions (Sigma-Aldrich L3287). Five days after transfection the supernatant was collected by first spinning cultures at 300 g for 5 min at 4°C. Cell debris and supernatant were further separated by spinning at 10,000 g for 20 min at 4°C. Upon resuspending in PBS, ACE2-Fc was purified over Protein A Agarose (Thermo Fisher 15918014). Once the Protein A Agarose was equilibrated in PBS buffer, the respective supernatant was applied 3x and washed with 10 bed volumes of PBS buffer. The protein was eluted with 100mM glycine-HCL pH 2.4 into 0.1x volume Tris buffer pH 8.5 and 100 mM NaCl. Purified ACE2-Fc was stored at 4°C for short-term storage, and frozen at −20°C for long-term storage.

Expression and purification of SARS-CoV-2 spike proteins

Plasmids were transfected into Expi293 cells and expressed as described previously (Hsieh et al., 2020). Variants were purified from 40mL cell culture. The supernatant was filtered by 0.22 μm filter and then run over StrepTactin Superflow column (IBA 2-1206-025). Spikes were further purified by Superose 6 increase 10/300 (GE GE29-0915-96) size-exclusion column in a buffer containing 2mM Tris pH 8.0, 200mM NaCl, and 0.02% NaN_3. Purified samples were stored at 4°C for short-term storage, and frozen at −20°C for long-term storage.

HEK293T transfection

Approximately 24 h before transfection, cells were seeded into 6-well or 12-well polystyrene coated plates (VWR 10861-696, 10861-698) at a density of 0.3×10^6 cells mL^{-1} or 0.1×10^6 cells mL^{-1}, respectively. Upon reaching 60%–80% confluence, cells were transfected with expression plasmids using Lipofectamine 3000 (Thermo Fisher L3000015) and Opti-MEM (GIBCO 51985091), following the manufacturer's instructions and 3 μL of lipofectamine per μg of plasmid DNA. Cells were assayed or collected 48 h post-transfection.

Flow cytometry and data analysis

HEK293T cells with surface-displayed spike were collected 48 h post-transfection by first washing once with PBS and then resuspending in PBS by gentle pipetting. Cell density was determined using a cell counter (Logos Biosystems L40002). Cells were then spun down at 200 g for 1 min. After decanting the supernatant cells were resuspended in chilled PBS-BSA (1% BSA (Sigma-Aldrich A3294), 1x PBS, 2 mM EDTA pH 7.4) to a density of $\sim 3 \times 10^6$ cells mL^{-1}.

Flow cytometry assays were prepared using an Axygen Deep well grow block (P-2ML-SQ-C-S). Each well contained a predetermined concentration of primary antibody or chimeric cell receptor (ACE2-Fc) diluted in PBS-BSA and 50 μL (1.5×10^5) of cells. Mixtures were incubated at room temperature and shaken at 950 rpm for 1 h. Cells were then pelleted by spinning the plate for 2 min at 500 g in a swinging bucket rotor. Cells were washed twice by decanting the supernatant and adding 500 μL of PBS-BSA to each well. 500 μL of a secondary antibody solution (5 μM Alexa Fluor 488 anti-mouse (SouthernBiotech 1031-30) and 10 μM Alexa Fluor 647 anti-human (SouthernBiotech 2048-31) in PBS-BSA) was added to each well. The plate was incubated in the dark, at 4 þC while shaking at 950 g for 25 min. Wells were washed again, twice with PBS-BSA, and then resuspended in 300 μL of PBS-BSA before running on the SA3800 Spectral Analyzer (SONY).

HEK293T cells were used to establish forward scatter-area (FSC-A) and side scatter-area (SSC-A) gating. Singlet discrimination was then established with forward scatter-height (FSC-H) versus forward scatter-area (FSC-A) and side scatter-height (SSC-H) versus side scatter-area (SSC-A) gates. A minimum of 10,000 singlet events were acquired for each assayed sample. These singlet HEK293T cells were further analyzed in two fluorescent channels, Alexa Fluor 488 (AF-488) and Alexa Fluor 647 (AF-647), using manufacturer-recommended excitation and detection settings. Spectral unmixing was applied to all data to reduce the effect of spectral spillover and autofluorescence on downstream calculations.

Median height (H) measurements for the AF-488 and AF-647 channels were recorded for each sample. Anti-FLAG (AF-488 channel) signal was used to measure spike expression. Spike variant (x) expression relative to WT (6P-D614G) was calculated using the following equation:

$$Normalized\ \ expression = \ Log_2\left(Median : 488\_H_x \Big/ Median : 488\_H_{6P\_D614G}\right)$$

Anti-FLAG signal was also used as an internal normalization control to correct for changes in transfection efficiency and spike expression when measuring antibody or ACE2 binding. Normalized binding measurements for spike variants (x) expression relative to WT (6P-D614G) was calculated using the following equation:

$$Normalized\ \ binding = \ Log_2\left(\frac{Median : 647\_H_x / Median : 488\_H_x}{Median : 647\_H_{6P\_D614G} \Big/ Median : 488\_H_{6P\_D614G}}\right)$$

For titration curves, median height (H) measurements for AF-647 were divided by the respective AF-488 signal for each sample. This provides a normalized binding measurement relative to each sample's expression signal (Anti-FLAG; AF-488). All titrations were conducted using biological triplicate (three separate transfections). All titration points for each curve were then divided by the max value in the set, thus normalizing the curve to a range between 0 and 1 normalized binding units.

All flow cytometry data were analyzed using FlowJo v9.

LCB1 cloning and purification

LCB1 was synthesized and cloned into *E. coli* using a pMAL expression vector via Hi-Fi DNA assembly (NEB E2621S) with a gBlock (IDT) encoding an N-terminal TEV cleavage site and the LCB1 gene sequence17 (Cao et al., 2020). The 6xHis-MBP-LCB1 was transformed into chemically competent Rosetta (DE3) cells (Sigma-Aldrich 70954). Protein expression was performed using LB media supplemented with carbenicillin, induced with isopropyltio-β-galactoside (IPTG) and grown overnight. Cells were pelleted at 4,000 g for 10 min and resuspended in lysis buffer containing 30 mM Tris-HCl pH 7.5 and 300 mM NaCl with DNase and protease inhibitor tablets. The cells were lysed by sonication while on ice. Soluble material was then clarified by centrifugation at 35,000 g for 45 min. The soluble fraction was added to a 5 mL HisTrap HP column (GE Healthcare 17524802) and eluted with elution buffer containing 30 mM Tris-HCl pH 7.5, 300 mM NaCl, and 250 mM Imidazole. The 6xHis-MBP-LCB1 was dialyzed overnight with TEV protease (BioVision 7847-100) at 4°C in buffer containing 30 mM Tris-HCl pH 7.5 and 300 mM NaCl. The TEV protease and 6xHis-MBP tag were removed by running the protein on a 5 mL HisTrap HP column and collecting the flowthrough.

LCB1 and ACE2 competition assays

Spike displaying HEK293T cells were collected and incubated with varying concentrations of LCB1 peptide and a fixed (25 nM) ACE2-Fc concentration. Anti-FLAG (Sigma-Aldrich F3165) was also included at 1 μg mL^{-1} final concentration. Cells were incubated at room temperature in an Axygen Deep well grow block (P-2ML-SQ-C-S), shaking at 950 rpm for 1 h. Cells were then washed with PBS-BSA and stained with secondary antibodies (5 μM Alexa Fluor 488 anti-mouse (SouthernBiotech 1031-30) and 10 μM Alexa Fluor 647 anti-human (SouthernBiotech 2048-31) in PBS-BSA), shaking at 950 rpm for 1 h at 4°C. After two final washes with PBS-BSA, cells were resuspended in PBS-BSA and run on the SA3800 Spectral Analyzer (SONY). Data were analyzed using FlowJo v9.

Cell immunostaining and microscopy

HEK293T cells were seeded into imaging dishes (Eppendorf 0030740009) and transfected with Spike Display plasmids. After 48 h, cells were gently washed twice with PBS and then fixed with 4% formaldehyde for 20 min at room temperature. Cells were washed again with PBS and then blocked with PBS-BSA (1%) blocking buffer for 20 min. Primary antibodies (anti-FLAG M2; Sigma and chimeric ACE2-Fc (Genscript Z03484) were diluted in PBS-BSA to 1 μg mL^{-1} and added to each imaging dish, incubating at room temperature for 1 h. Cells were washed twice with PBS-BSA. Secondary antibodies (Alexa Fluor 488 (SouthernBiotech 1031-30); Alexa Fluor 647 (SouthernBiotech 2048-31) were diluted in PBS-BSA to 10 μg mL^{-1} and added to each imaging dish, incubating at room temperature for 1 h in complete darkness. Cells were washed twice with PBS-BSA. Hoechst stain (Thermo Fisher 62249) was diluted 1:10,000 in PBS-BSA and added to each imaging dish, incubating at room temperature for 15 min. Cells were washed once with PBS-BSA and a final 1 mL of PBS-BSA was added before imaging.

All images were collected with a Nikon Ts2R-FL inverted research microscope equipped with a CF160 Plan Apochromat Lambda 60x oil immersion objective lens. Hoechst stain, Alexa Fluor 488, and Alexa Fluor 647 were excited by a SOLA SM II 365 light engine and filtered through C-FL Hoechst (AT460/50 m), C-FL FITC (AT535/40 m), and C-FL mCherry (ET630/75 m) filter cubes (Chroma), respectively. Images were acquired with a pco.panda sCMOS camera controlled with NIS-Elements D software.

SARS-COV-2 pseudovirus neutralization assay

Most of the plasmids needed for expressing the HIV virion under the CMV promoter were obtained from BEI resources, including HDM-Hgpm2, pRC-CMV-Rev1b, and HDM-tat1b (BEI catalog numbers NR-52516, NR-52519, and NR-5251, respectively)

(Crawford et al., 2020). The lentiviral backbone plasmid, expressing a luciferase reporter under the CMV promoter followed by an IRES and ZsGreen, was also provided by the BEI resources (NR-52520). The envelope plasmid (HDM-IDTSpike-fixK) expresses a codon-optimized WT spike protein of SARS-COV-2 under a CMV promoter (GenBank ID: NC_045512) and was supplied by BEI resources. It was used as a template for site-directed mutagenesis to generate new expression plasmids for the D614G and B.1.1.7 variants. A pCMV-VSV-G plasmid (Cell Biolabs RV-110) was used for expressing the VSV-G (vesicular stomatitis virus glycoprotein). The lentiviral plasmid used to generate the HEK293T stable cell lines expressing the human ACE2 gene (GenBank ID NM_021804) under an EF1a promoter was obtained from the BEI resources as NR52516 (Crawford et al., 2020).

The HIV particles pseudotyped with SARS-CoV-2 spike variants D614G or B.1.1.7 were generated in HEK293T cells, following previously published protocols (Crawford et al., 2020). Cells were transiently co-transfected with plasmids for (1) HIV virion formation proteins (HDM-Hgpm2, pRC-CMV-Rev1b, and HDM-tat1b); (2) one of the envelope proteins (2019-nCoV Spike-D614G mutant, B.1.1.7 variant or VSV-G) and (3) the lentiviral backbone expressing luciferase reporter (pHAGE-CMV-Luc2-IRES-ZsGreen-W). Media was exchanged for new media 24 h post-transfection. Media containing the pseudovirus particles were collected, filtered, and fractionated 72 h post-transfection. Fractions were stored at -80°C until later usage. Lentivirus titer was estimated for each virus particle using the qPCR Lentivirus Titer Kit (abm LV900), following the manufacturer's protocol. Around 106 infectious units of the virus were incubated with each dose of the tested antibody in full media (100 μL) for 1 h at room temperature. The mixture was then added to HEK293T target cells, stably expressing human ACE2 in 96-well white plates with a clear bottom. The % cell confluency in each well was estimated after 60 h of incubation using the IncuCyte ZOOM (Essen BioScience) with a 10X objective. Cells were then treated with Bright-Glo Luciferase Assay System (Promega E2610) to detect luciferase signal (relative luciferase units or RLU) following the manufacturer protocol. The ratio of recorded relative luciferase units in the antibody's presence to the readout in the absence of the antibody was employed to estimate the percentage of virus entry at each dose. Half-maximal inhibitory concentrations (IC_{50}) were calculated using a 3-parameter logistic regression equation (GraphPad Prism v9.0). Experiments were performed in duplicate using different preparations of virus.

Western blots

Equal cell numbers were collected 48 h post-transfection for each sample. Proteins were extracted using RIPA buffer with protease inhibitors (cOmplete, EDTA-free Protease Inhibitor Cocktail; Millipore Sigma 11873580001). Sample input was normalized using a Bradford protein assay that was calibrated with BSA standards. For each sample, 10 μg of total protein was run on a 4%–15% SDS-PAGE gel (Bio-Rad 4561084). Proteins were then transferred onto a PVDF membrane using a Trans-Blot SD. Semi-Dry Transfer Cell (Bio-Rad 1703940) per the manufacturer's instructions. Membranes were blocked in Odyssey Blocking Buffer (PBS) (Li-COR 927-40000) incubated with the following primary antibodies overnight: mouse anti-FLAG M2 antibody (1:10,000; Sigma-Aldrich F3165) for spike protein or RBD detection and mouse anti-beta tubulin antibody (1:5000; Thermo Fisher MA5-16308) as a loading control. The membrane was washed three times with PBST (1x PBS, 0.1% Tween 20) for 10 min and then incubated with the following secondary antibody for 2 h: goat anti-mouse IRDYe 680 conjugated antibody (1:10,000; abcam ab216776). The membrane was washed again with PBST three times for 10 min and imaged on the Odyssey LI-COR. Irrelevant and superfluous lanes from the blot image were removed for clarity. All raw image files are available on Mendeley.

Spike Display cleavage and spike purification

HEK293T cells were transfected with plasmids encoding Spike Display variants and collected 48 h post-transfection by washing once with PBS and resuspending 3-4 × 10^6 cells in a 1.5 mL Eppendorf tube in 1 mL of 3C cleavage buffer (150 mM NaCl, 50 mM Tris-HCl pH 8.0). Five units of 3C protease (Thermo Fisher 88946) were added to each tube and tubes were placed on a shaker at room temperature and 900 rpm for 1 h to separate spike trimers from the cell surface. Supernatant containing the spike protein was collected by spinning tubes at 16,000 g for 1 min and transferring ~1 mL of supernatant to a fresh tube. Supernatants were kept on ice until analysis. For EM imaging, supernatants were purified through a 0.5 mL StrepTactin resin (IBA) column. After spin concentrating the elution fractions to 1 mL, spikes were further purified by size-exclusion chromatography using a Superose 6 Increase 10/300 column (GE Healthcare).

Negative stain EM data collection and processing

Purified SARS-CoV-2 spike variants were diluted to a concentration of 40 μg mL^{-1} in 50 mM Tris-HCL pH 8.0 and 150 mM NaCl. EM grids (Electron Microscopy Science CF400-Cu) were cleaned in a Gatan Solarus 950 plasma cleaner for 45 s. Spikes were deposited on the girds and stained with Nano-W (Nanoprobes #2018). Grids were then imaged on a Talos F200C TEM microscope (Thermo Fisher) equipped with a Ceta 16M detector (Thermo Fisher). 106 micrographs were imaged from a single grid at a magnification of 92,000X, corresponding to a calibrated pixel size of 1.63 Å/pix. Motion correction, CTF estimation and particle picking were performed in CisTEM (Grant et al., 2018). Particle stacks were then analyzed by cryoSPARC v3.1.0 software for 2D classification, *ab initio* 3D reconstruction, heterogeneous 3D refinement, and homogeneous 3D refinement (Punjani et al., 2017).

Biolayer Interferometry

After Cleavage with 3C protease, supernatants containing spikes variants were diluted 2-fold with BLI buffer composed of 10 mM HEPES pH 7.5, 150 mM NaCl, 3 mM EDTA, 0.05% v/v Surfactant P20 (Cytiva BR100054), 1 mg mL^{-1} bovine serum albumin. Analytes

were also serial diluted with BLI buffer. Anti-mouse Fc capture (AMC) biosensors (FortéBio 18-5088) were hydrated with BLI buffer for 10 min in an Octet RED96e (FortéBio). Then, Mouse anti-FLAG M2 (Sigma-Aldrich F3165) antibodies were immobilized to the AMC sensor tips. The following steps were used for each assay: 1) baseline: 60 s with BLI buffer; 2) IgG immobilizing: 360 s with anti-FLAG IgG; 3) spike loading: 360 s with diluted supernatants; 4) baseline: 300 s with BLI buffer; 5) association: 600 s with serial diluted analytes (antibodies or ACE2); 6) dissociation: 600 s with BLI buffer. The data were reference-subtracted and analyzed by Octet Data Analysis software v11.1 using steady-state analysis.

Computational analysis of GISAID sequence data

To identify and obtain SARS-CoV-2 spike protein variants of interest, we performed pairwise sequence alignment of all spike proteins in the GISAID EpiCoV database and the SARS-CoV-2 reference sequence with the GenBank number BCN86353.1 (Elbe and Buckland-Merrett, 2017). We downloaded all published spike protein sequences from the GISAID (accessed on 2/24/2021) as a FASTA file. We then used the Biopython Bio.SeqIO package (https://biopython.org/wiki/SeqIO) to extract the spike protein amino acid sequence and related species information from each entry in the FASTA file. Preliminary filtering was performed to remove non-human sequences, sequences with more than 800 unknown amino acid sequences (“X”), and sequences with < 1250 or > 1280 amino acids. 484,744 out of the 606,304 sequences passed these filters. GB number BCN86353.1 (Elbe and Buckland-Merrett, 2017).

We then used the python parasail package (https://github.com/jeffdaily/parasail-python) to perform a semi-global alignment of each sequence to the reference sequence (Daily, 2016). Parameters were set to no penalties for gaps at the beginning and end of both sequences using a BLOSUM80 substitution scoring matrix, a gap opening penalty of 5 within the sequence, and a gap extension penalty of 2 within the sequence. A BLOSUM80 scoring matrix was used because higher number blosum matrices (blosum80 as opposed to blossom32) compare closely related sequences. The highest scoring sequence alignment was kept, and the aligned sequence was compared position-wise to the reference sequence to identify insertions, deletions, and substitutions. Frequencies of each unique substitution, insertion, and deletion were obtained with the following equation:

$$\text{frequency} = \sum(\text{sequences containing each mutation}) \div \sum(\text{all sequences aligned})$$

Data analysis was performed with the R tidyverse package and ggplot2 (Wickham et al., 2019).

Structural analyses and data visualization

All structures (7DDN (Zhang et al., 2021), 7C2L (Chi et al., 2020), and 7JZL (Cao et al., 2020)) were downloaded from the RCSB-PDB as PDB files and imported into ChimeraX 1.1 for data visualization and image creation. Log2(normalized binding) values were rescaled (−7 to 0 or −1 to 0) and converted to monochromatic ChimeraX color codes with darker red signifying a greater loss in binding and white signifying no change in binding relative to 6P-D614G spike. These colors scales were superimposed on spike protein structures for each amino acid screened by our Spike Display assay. For figures showing grouped antibody epitopes, normalized binding values for all mutations in each position were averaged for all the antibodies comprising that group. These averaged binding values were then converted to a single color and mapped onto spike structures.

QUANTIFICATION AND STATISTICAL ANALYSIS

The means ± SD were calculated and reported for all appropriate data. NTD-targeting nAbs were group based on Pearson correlation r values > 0.5.

Molecular Cell, Volume *81*

Supplemental information

Rapid characterization of spike variants

via mammalian cell surface display

Kamyab Javanmardi, Chia-Wei Chou, Cynthia I. Terrace, Ankur Annapareddy, Tamer S. Kaoud, Qingqing Guo, Josh Lutgens, Hayley Zorkic, Andrew P. Horton, Elizabeth C. Gardner, Giaochau Nguyen, Daniel R. Boutz, Jule Goike, William N. Voss, Hung-Che Kuo, Kevin N. Dalby, Jimmy D. Gollihar, and Ilya J. Finkelstein

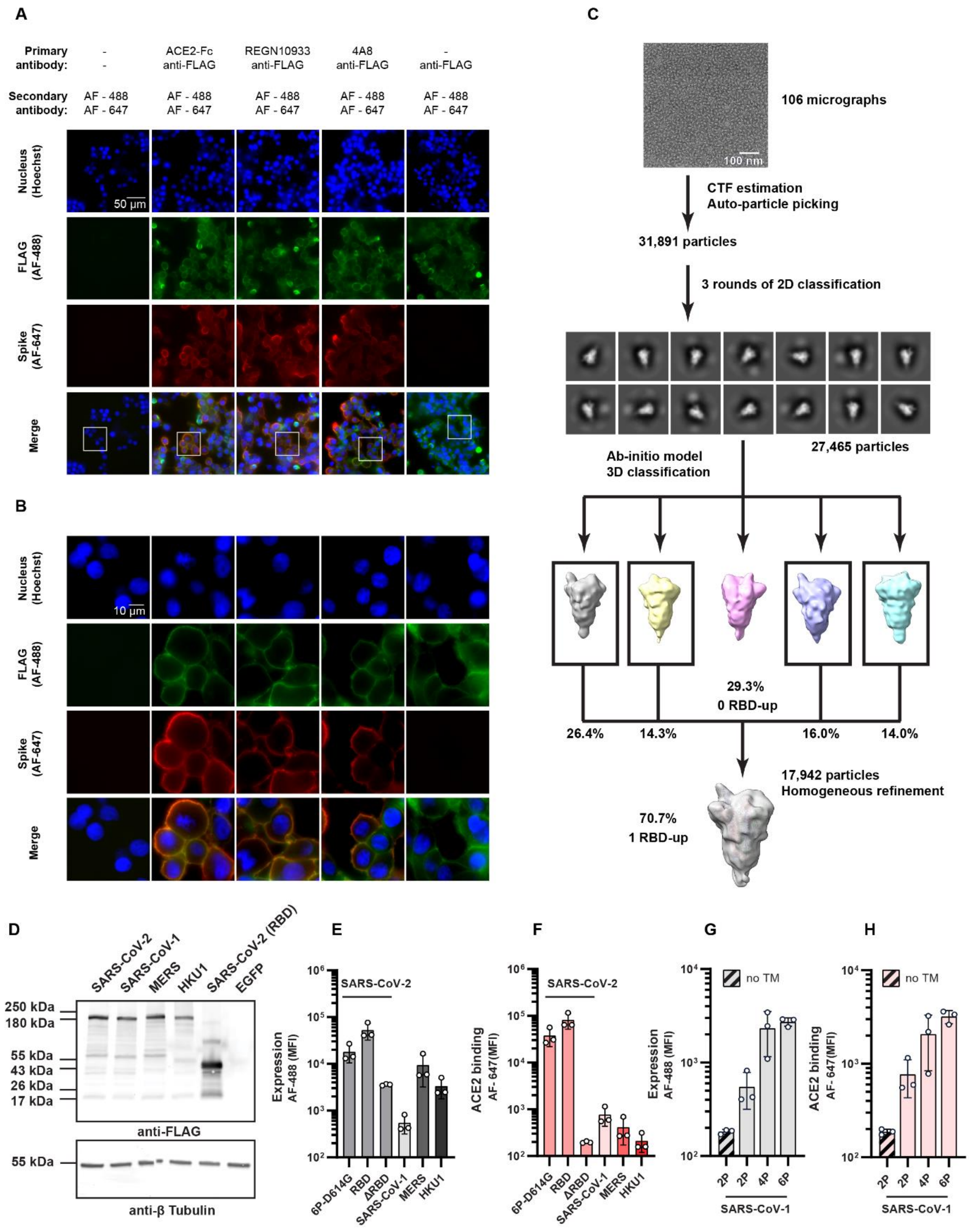
A
Primary antibody:
ACE2-Fc anti-FLAG
REGN10933 anti-FLAG
4A8 anti-FLAG
anti-FLAG
Secondary antibody:
AF - 488
AF - 647
Nucleus (Hoechst)
FLAG (AF-488)
Spike (AF-647)
Merge
50 μm
B
10 μm
C
106 micrographs
100 nm
CTF estimation
Auto-particle picking
31,891 particles
3 rounds of 2D classification
Ab-initio model
3D classification
27,465 particles
29.3%
0 RBD-up
26.4%
14.3%
16.0%
14.0%
17,942 particles
Homogeneous refinement
70.7%
1 RBD-up
D
SARS-CoV-2
SARS-CoV-1
MERS
HKU1
SARS-CoV-2 (RBD)
EGFP
250 kDa
180 kDa
55 kDa
43 kDa
26 kDa
17 kDa
anti-FLAG
55 kDa
anti-β Tubulin
E
SARS-CoV-2
Expression AF-488 (MFI)
6P-D614G
RBD
ΔRBD
SARS-CoV-1
MERS
HKU1
F
SARS-CoV-2
ACE2 binding AF- 647(MFI)
G
no TM
Expression AF-488 (MFI)
2P
2P
4P
6P
SARS-CoV-1
H
no TM
ACE2 binding AF- 647(MFI)
SARS-CoV-1

Figure S1. Characterization of surface displayed SARS-CoV-2 spikes and related homologs, Related to Figure 1

(A) Fluorescent immunostaining of HEK293T cells transiently expressing SARS-CoV-2 spike (6P-D614G). Cells were immunostained 48 hours post transfection with the spike display construct. Full field view of HEK293Ts at 60X magnification. Top row indicates how cells were immunostained; the first column is a negative control. Last column shows no AF-647 cross-reactivity with anti-FLAG antibody.
(B) Higher magnification images from the white rectangular frames in (A). AF = Alexa Fluor.
(C) Schematic of pre-processing, classification, and refinement procedure to generate the map from 106 micrographs (STAR methods). Boxed maps indicate the maps showing 1-RBD up EM density in alignment with HexaPro (S-6P) 1-RBD up structure (6XKL) (Wrapp et al., 2020).
(D) Western blot of spike displayed proteins and RBD from HEK293T cells. Western blots were stained with anti-FLAG antibodies. EGFP is included as a negative control for anti-FLAG, and anti-β tubulin used as a loading control.
(E-F) Raw expression (E) and ACE2 binding (F) signals measured via spike display and flow cytometry. The ΔRBD construct is a negative control for background ACE2 binding.
(G-H) Raw expression (G) and ACE2 binding (H) signals for pre-fusion stabilized SARS-CoV-1 using prolines. 2P = F799P + A874P. 4P = 2P + A881P + S924P. 6P = 4P + K968P + V969P. no TM = SARS-CoV-1 (2P) with no transmembrane domain, used as a negative control for background expression and ACE2 binding. Circles represent biological replicates. Error bars = S.D.

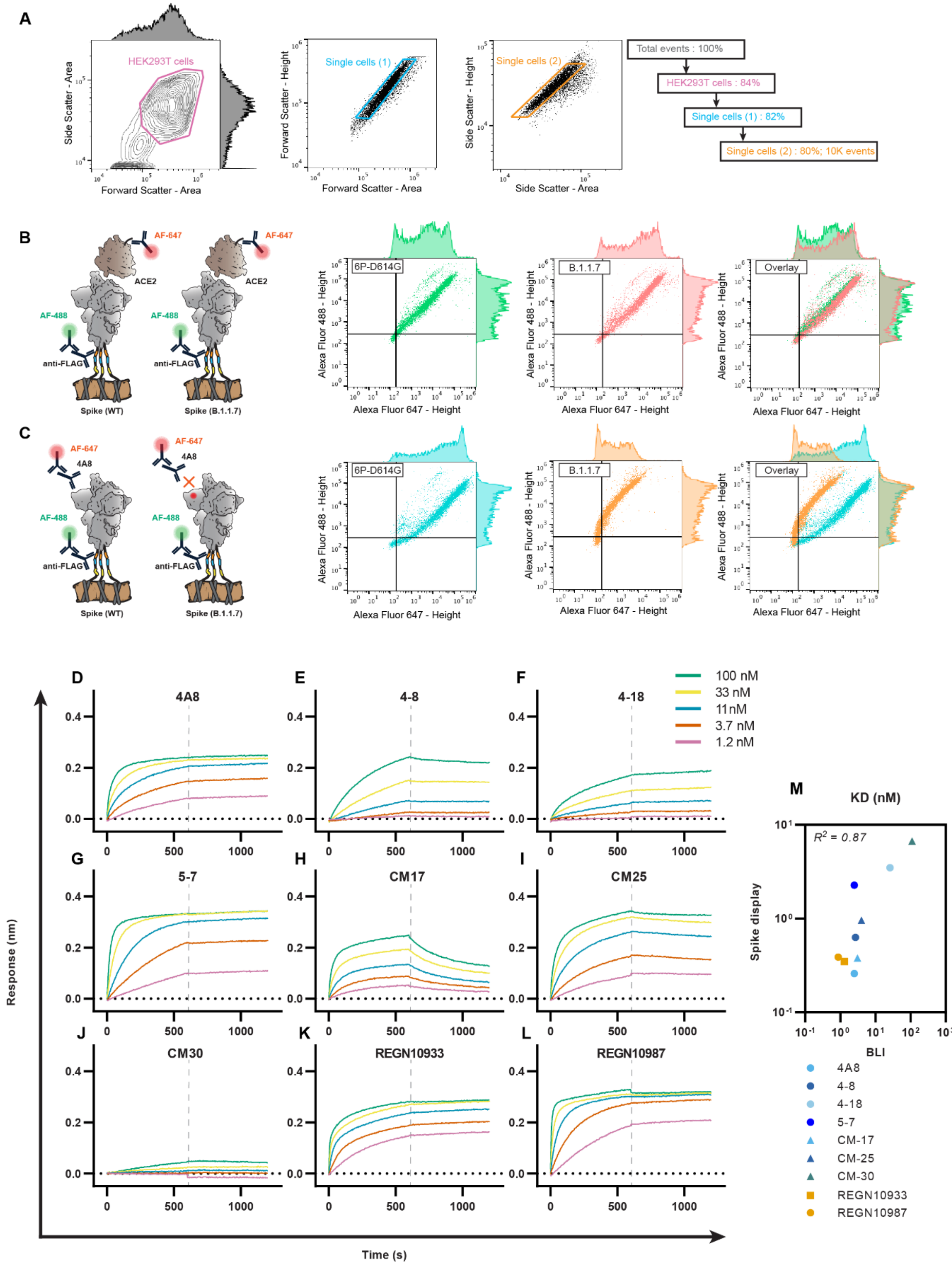
A
HEK293T cells
Side Scatter - Area
Forward Scatter - Area
Single cells (1)
Forward Scatter - Height
Single cells (2)
Side Scatter - Height
Side Scatter - Area
Total events : 100%
HEK293T cells : 84%
Single cells (1) : 82%
Single cells (2) : 80%; 10K events
B
AF-647
ACE2
AF-488
anti-FLAG
Spike (WT)
Spike (B.1.1.7)
6P-D614G
B.1.1.7
Overlay
Alexa Fluor 488 - Height
Alexa Fluor 647 - Height
C
4A8
D
E
F
4-8
4-18
100 nM
33 nM
11nM
3.7 nM
1.2 nM
G
H
I
5-7
CM17
CM25
J
K
L
CM30
REGN10933
REGN10987
Response (nm)
Time (s)
M
KD (nM)
R^2 = 0.87
Spike display
BLI
4A8
4-8
4-18
5-7
CM-17
CM-25
CM-30
REGN10933
REGN10987

Figure S2. Flow cytometry data processing and biolayer interferometry (BLI) of nAb binding to spike (6P-D614G), Related to Figure 1

(A) Representative gating used to select for single HEK293T cells expressing surface displayed spikes. HEK293T cells identified with side scatter-area (SSC-A) vs forward scatter-area (FSC-A) gating followed by doublet discrimination using forward scatter-height (FSH) vs forward scatter-area (FSC-A) and side scatter-height (SSC-H) vs side scatter-area (SSC-A) gates. Representative cell% retained from each gating step shown on the right.
(B) Graphical representation of the antibodies used (left) and the resulting flow cytometry data measuring ACE2 binding of 6P-D614G and B.1.1.7 spike variants (right). Single cells were analyzed using two fluorescent channels, Alexa Fluor-488 and Alexa Fluor-647 for measuring spike expression and antigenicity, respectively.
(C) Graphical representation (left) and flow cytometry data measuring 4A8 antibody binding of 6P-D614G and B.1.1.7 spike variants (right).
(D-L) Histograms representing signal distributions for Alexa Fluor-488 and Alexa Fluor-647 are included for (B) and (C). On- and off-rate curves for antibodies (D) 4A8, (E) 4-8, (F) 4-18, (G) 5-7, (H) CM17, (I) CM25, (J) CM30, (K) REGN10933, and (L) REGN10987.
(M) Correlation of the binding affinities measured by spike display and BLI.

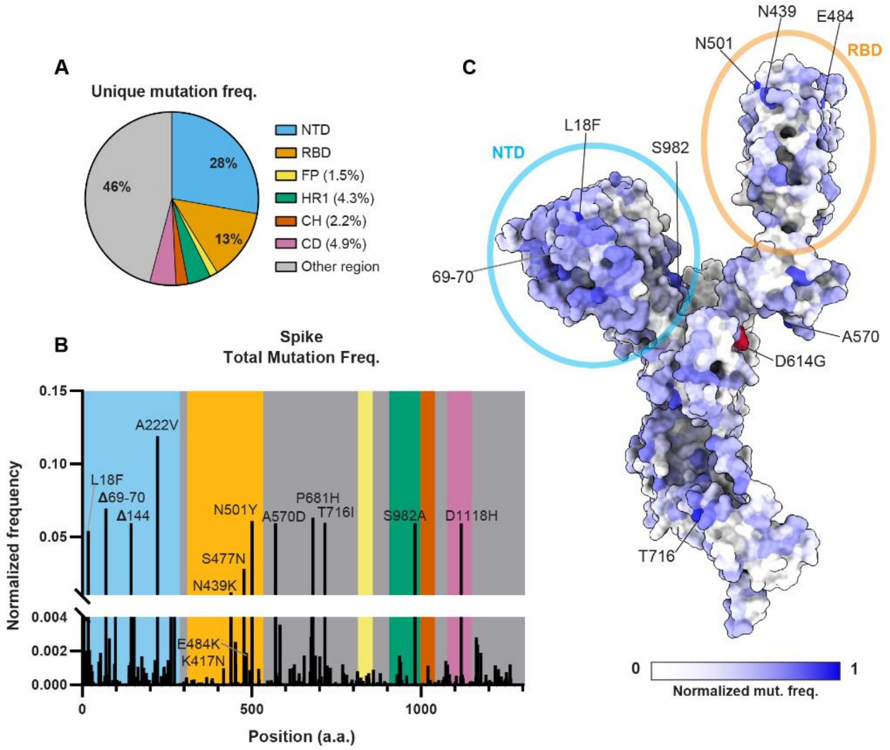

Figure S3. Summary of SARS-CoV-2 spike mutation frequencies, Related to Figure 2

(A) The distribution of spike mutations across key protein domains, as reported in the GISAID (accessed on 24/Feb/2021) (Elbe and Buckland-Merrett, 2017; Shu and McCauley, 2017).
(B) Normalized mutation frequencies, excluding the globally dominant D614G substitution. Spike domains are colored on the graph as in (A) and Figure 2A.
(C) A spike ectodomain monomer colored by mutation frequency (PDB: 7DDN (Zhang et al., 2021)). White: invariant position (no mutations). A few key residues are labeled. Position 614, labeled in red, is excluded from the frequency calculations because it is now globally dominant (Korber et al., 2020).

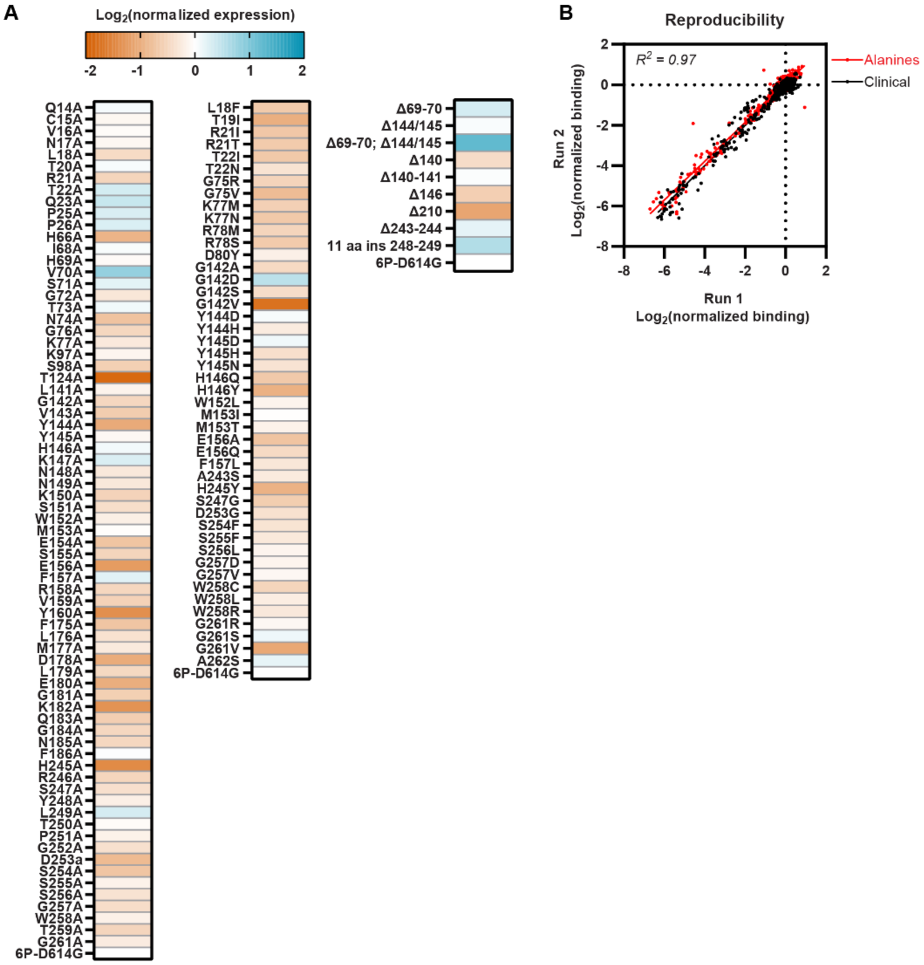

Figure S4. Spike expression measurements and assay reproducibility, Related to Figures 2 and 3

(A) Normalized spike expression for alanine and clinical variants in Figs. 2C, 3A, and 3E.
(B) Comparison of the nAb binding data across two biological replicates and all spike variants indicates excellent reproducibility. Red = alanine scan; Black = all clinical variants. Pearson correlation was identical for both datasets.

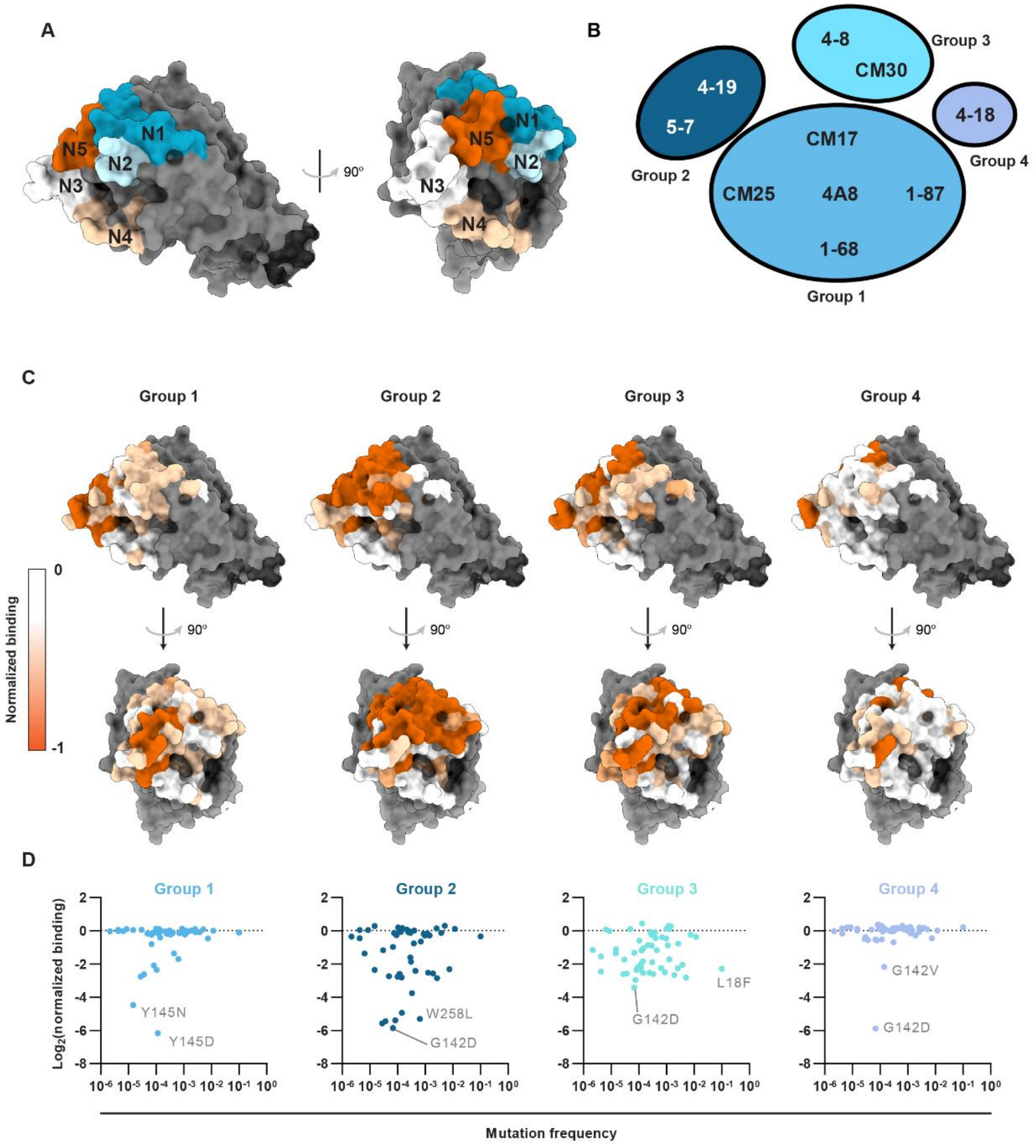
A
N1
N2
N3
N4
N5
90°
B
4-19
5-7
Group 2
4-8
CM30
Group 3
4-18
Group 4
CM17
CM25
4A8
1-87
1-68
Group 1
C
Group 1
Group 2
Group 3
Group 4
Normalized binding
0
-1
90°
D
Log2(normalized binding)
Y145N
Y145D
W258L
G142D
L18F
G142V
Mutation frequency

Figure S5. Antibody classification and epitope mapping, Related to Figure 3

(A) NTD loops N1 (residues 14-26), N2 (residues 67-79), N3 (residues 141-156), N4 (residues 177-186), and N5 (residues 246-260) uniquely colored and labeled on NTD structure (PDB: 7DDN (Zhang et al., 2021)).
(B) NTD binding nAbs grouped according to Pearson correlation matrix (r > 0.5) from Figure 3C.
(C) Normalized binding data from Figures 2C and 3A were averaged for all nAbs in each group and normalized to a -1(loss of binding) to 0 (no change in binding) scale and superimposed on the NTD (PDB: 7DDN (Zhang et al., 2021)). Gray: unmutated positions. Epitope maps highlight distinct binding patterns at the NTD supersite.
(D) Comparison of mutation frequency as a function of NTD nAb escape for each group.

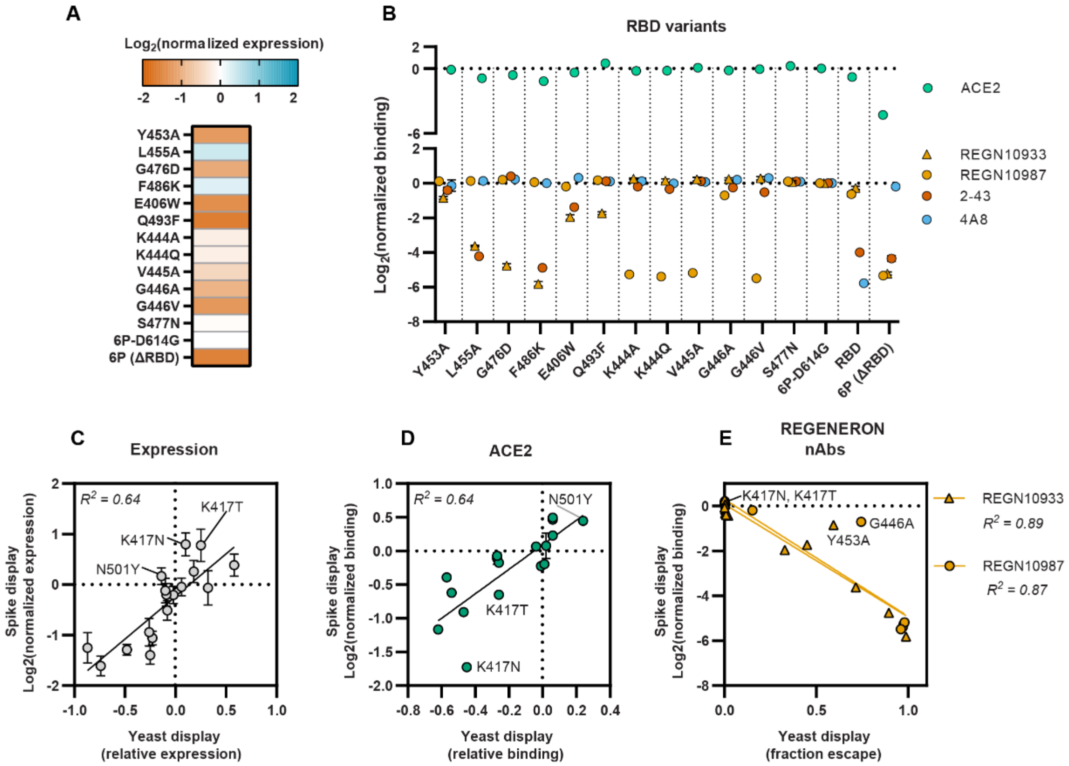

Figure S6. Analysis of RBD mutations and comparison to yeast RBD display, Related to Figure 3

(A-B) Expression (A) and binding (B) data for RBD variants that escape REGENERON nAbs REGN10933 and REGN10987. We also included the high prevalence S477N clinical mutation. Data in (A) and (C) were measured using spike display. Antibodies (2-43 and 4A8) and ACE2 were also screened. (n = 3 biological replicates; error bars = S.D.).
(C-E) Spike display correlates with yeast display of the isolated RBD (Starr et al., 2021, 2020) for (C) expression, (D) ACE2 binding, and (E) REGNERON nAb binding. Pearson correlations are indicated in each panel.

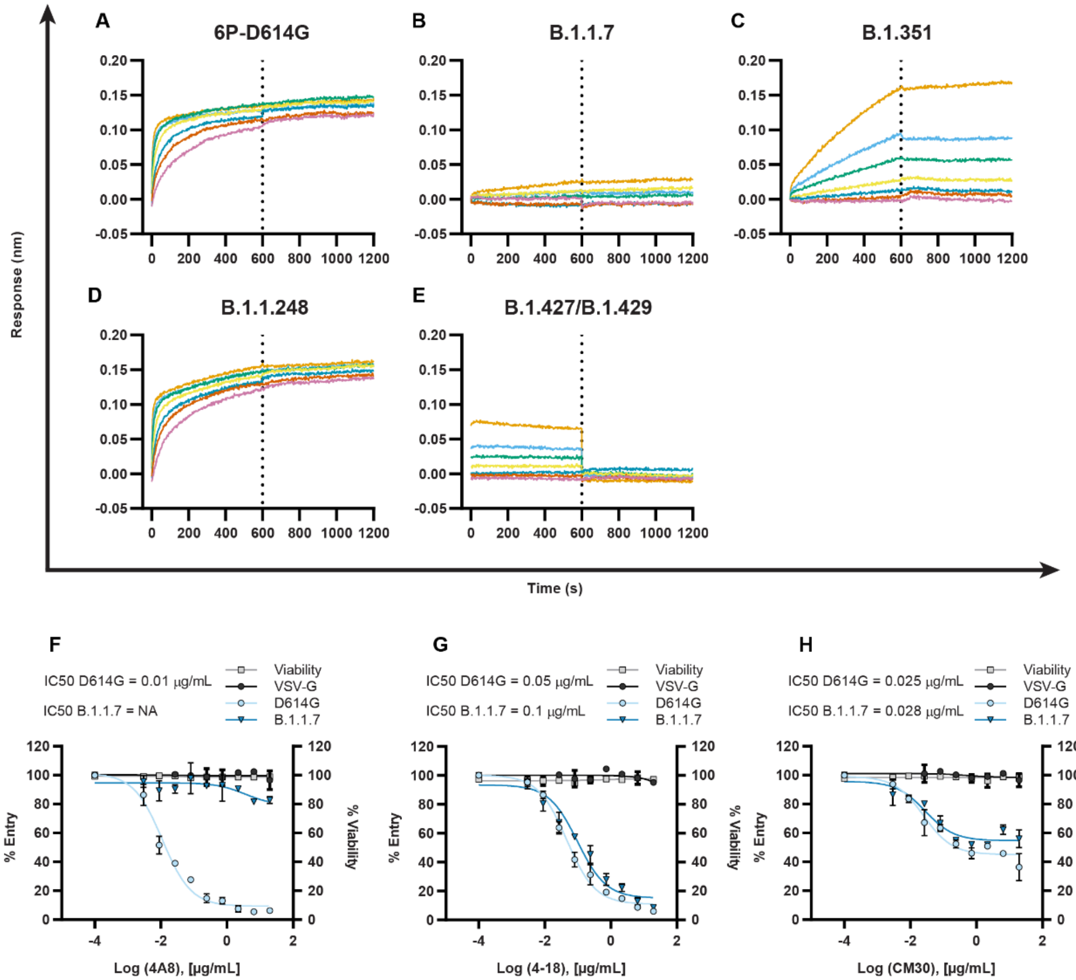

Figure S7. Biolayer interferometry of VOC spikes cleaved from cell surfaces and pseudovirus neutralization assays show nAb escape, Related to Figures 4 and 5

(A-E) BLI spectrogram showing the spike capture strategy. Anti-mouse Fc (AMC) tips are loaded with mouse anti-FLAG antibodies. The tips then capture SARS-CoV-2 spikes cleaved from cell surfaces with 3C protease (STAR methods). BLI tips are further incubated in antibody (4A8) solutions of varying concentrations to observe "on" and "off" rates. (A-E) BLI on and off rate curves for 4A8 against 6P-D614G (A), B.1.1.7 (B), B.1.351 (C), B.1.1.248 (D), and B.1.427/B.1.429 (E) spikes. BLI experimental design matches that shown in (A). On/off rate transition for (A-E) indicated by vertical dotted lines.

(F-H) SARS-CoV-2 pseudovirus neutralization curves using 4A8 (F), 4-18 (G), and CM30 (H) nAbs. Results compare D614G (light blue) and B.1.1.7 (dark blue) pseudovirus variants (STAR methods). VSV-G (black) included as a negative control for neutralization. Cell viability data included in gray. Dots and error bars indicate mean and S.D of three replicates.

Table S1. Comparison of antibody binding affinities using different platforms, Related to Figure 1

Antibody	Specificity	Spike Display K_D (nM)	BLI K_D (nM)°	Literature K_D (nM)
4A8 (Chi et al., 2020)	NTD	0.26 ± 0.04	2.5 ± 0.06	0.996 ± 0.045[*]
4-8 (Liu et al., 2020)	NTD	0.63 ± 0.04	2.7 ± 0.7	0.150 ± 0.037[#,§]
4-18 (Liu et al., 2020)	NTD	3.5 ± 0.2	26 ± 4	0.216 ± 0.040[#,§]
5-7 (Liu et al., 2020)	NTD	2.3 ± 0.1	2.5 ± 0.3	0.0950 ± 0.011[#,§]
CM17 (Voss et al., 2021)	NTD	0.38 ± 0.04	3.1 ± 1	9.34 ± 0.16[#]
CM25 (Voss et al., 2021)	NTD	0.96 ± 0.03	4.0 ± 0.2	9.44 ± 0.055[#]
CM30 (Voss et al., 2021)	NTD	6.7 ± 0.6	110 ± 30	0.841 ± 0.84[#]
2-43 (Liu et al., 2020)	S1	0.22 ± 0.2	N/A	1.66 ± 1.5[&]
REGN10933 (Hansen et al., 2020)	RBD	0.35 ± 0.05	1.3 ± 0.2	0.0417[@]
REGN10987 (Hansen et al., 2020)	RBD	0.39 ± 0.02	0.87 ± 0.07	0.0428[@]

° Measured in this study using the full IgG.

* Measured via bio-layer interferometry (BLI).

Measured via enzyme-linked immunosorbent assays (ELISAs)

@ Measured via surface plasmon resonance (SPR).

§ Digitized with WebPlotDigitizer and fit with a 4PL curve in GraphPad Prism 9. Error bar: 95% CI of the fit.

& Digitized with WebPlotDigitizer and fit with a 4PL curve in GraphPad Prism 9. Original data were measured via cell-surface competition binding assay. Error bar: 95% CI of the fit.

Table S2. Biolayer interferometry of 4A8 to spike variants of concern, Related to Figure 4 and Figure S7

Lineage	4A8 mAb K_D (nM)
WT	2.3 ± 0.4
B.1.1.7	N/D
B.1.351	34 ± 3
B.1.1.248	2.0 ± 0.4
B.1.427/B.1.429	N/D

N/D: Not determined because 4A8 binding was below the detection limit.

Table S3. Synthetic DNA sequences for cloning, Related to STAR methods

Oligo ID	DNA sequence (5’ to 3’)	Purpose
Primers		
P1	TACCACGCGTGAATTCTCGAGG	PCR of pcDNA5 bb (Rev)
P2	CGGCCGCTCGAGTCTAGAG	PCR of pcDNA5 bb (Fwd)
P3	ATGTTCGTGTTCCTGGTGCTCC	PCR of SARS-CoV-2 Spike (Fwd)
P4	GCCCAGGAATGTGCTCAGC	PCR of SARS-CoV-2 Spike (Rev)
P5	tgaacgctgaCTGCAGGCAAGCTTGGCAC	PCR of MBP-TEV bb (Fwd)
P6	ggttttcagaCGAATTAGTCTGCGCGTCTTTC	PCR of MBP-TEV bb (Rev)
P7	gactaattcgTCTGAAAACCTGTATTTCCAG	PCR LCB1 insert (Fwd)
P8	ttgcctgcagTCAGCGTTCAACTTCCTC	PCR LCB1 insert (Rev)
P9	acctgccaccTGTGGGCTACCTGCAACC	PCR of Spike Display bb for Part1 DO plasmid (Fwd)
P10	acctgccaccGTCACCAGTGGAACCTGG	PCR of Spike Display bb for Part1 DO plasmid (Rev)
P11	cactggtgacGGTGGCAGGTGGAAAGTG	PCR of sfGFP DO insert for Part1 DO plasmid (Fwd)
P12	gtagcccacaGGTGGCAGGTGTATAAACG	PCR of sfGFP DO insert for Part1 DO plasmid (Rev)
P13	acctgccaccGCCACCGTCTGCGGTCCT	PCR of Spike Display bb for Part2 DO plasmid (Fwd)
P14	acctgccaccCACATAGTAGGCTGCAGCTCCG	PCR of Spike Display bb for Part2 DO plasmid (Rev)
P15	ctactatgtgGGTGGCAGGTGGAAAGTG	PCR of sfGFP DO insert for Part2 DO plasmid (Fwd)
P16	agacggtggcGGTGGCAGGTGTATAAACG	PCR of sfGFP DO insert for Part2 DO plasmid (Rev)
P17	GCGTTTATACACCTGCCACC GATCTACAAGACCCCACCTATCAAGGAC	PCR of Spike Display bb for Part3 DO plasmid (Fwd)
P18	TTCACTTTCCACCTGCCACC TGGCGGGGGCGTGGAGTAAC	PCR of Spike Display bb for Part3 DO plasmid (Rev)
P19	GTTACTCCACGCCCCCGCCA GGTGGCAGGTGGAAAGTGAA	PCR of sfGFP DO insert for Part3 DO plasmid (Fwd)
P20	GTCCTTGATAGGTGGGGTCTTGTAGAT CGGTGGCAGGTGTATAAACGC	PCR of sfGFP DO insert for Part3 DO plasmid (Rev)
P21	ACCTGCCACCCAGAGCGCGCCGCAC	PCR of Spike Display bb for Part4 DO plasmid (Fwd)
P22	ACCTGCCACCGATCTGCTTCACTTGGGCG	PCR of Spike Display bb for Part4 DO plasmid (Rev)
P23	GAAGCAGATCGGTGGCAGGTGGAAAGTG	PCR of sfGFP DO insert for Part4 DO plasmid (Fwd)
P24	GCGCGCTCTGGGTGGCAGGTGTATAAACG	PCR of sfGFP DO insert for Part4 DO plasmid (Rev)

P25	ACCTGCCACCGCAGGGATCCGGATACATCCC CG	PCR of Spike Display bb for Part5 DO plasmid (Fwd)
P26	ACCTGCCACCTCTGGGGGAAGCTCATCAGG	PCR of Spike Display bb for Part5 DO plasmid (Rev)
P27	TTCCCCCAGAGGTGGCAGGTGGAAAGTG	PCR of sfGFP DO insert for Part5 DO plasmid (Fwd)
P28	GGATCCCTGCGGTGGCAGGTGTATAAACG	PCR of sfGFP DO insert for Part5 DO plasmid (Rev)
P29	acctgccaccGCAGGGATCCGGATACATC	PCR of Spike Display bb for Part1-5 DO plasmid (Fwd)
P30	acctgccaccGTCACCAGTGGAACCTGG	PCR of Spike Display bb for Part1-5 DO plasmid (Rev)
P31	cactggtgacGGTGGCAGGTGGAAAGTG	PCR of sfGFP DO insert for Part1-5 DO plasmid (Fwd)
P32	ggatccctgcGGTGGCAGGTGTATAAACG	PCR of sfGFP DO insert for Part1-5 DO plasmid (Rev)
gBlocks		
G1	CCTCGAGAATTCACGCGTGGTACCTCTAGAG TCGACCCGGGATGGAGACAGACACACTCCTG CTATGGGTACTGCTGCTCTGGGTTCCAGGTTC CACTGGTGACATGTTCGTGTTCCTGGTGCT	Ig-Kappa leader
G2	TGCTGAGCACATTCCTGGGCAGATCCGACTA CAAAGACCATGACGGTGATTATAAAGATCAT GACATCGATTACAAGGATGACGATGACAAG AGCGCCTGGTCCCACCCCCAGTTCGAGAAGG GATCCCTGGAGGTGCTGTTCCAGGGCCCAGG CGGCGGCGGTAGTGGAGGGGGCGGATCTGG CGGCTCAGCTGCTGTGGGCCAGGACACGCAG GAGGTCATCGTGGTGCCACACTCCTTGCCCTT TAAGGTGGTGGTGATCTCAGCCATCCTGGCC CTGGTGGTGCTCACCATCATCTCCCTTATCAT CCTCATCATGCTTTGGCAGAAGAAGCCACGT TAACGGCCGCTCGAGTCTAG	C-terminal linker
G3	ACCAACAAGGACCATAGCATATGTCCCATCA CCATCACCATCACCATCACTCTGAAAACCTG TATTTCCAGAGCGGCGGTGGCGACAAGGAAT GGATCCTGCAGAAAATTTATGAAATCATGCG CCTCCTGGACGAGCTGGGCCACGCTGAAGCG TCCATGCGTGTTTCCGACCTGATCTACGAATT CATGAAGAAAGGCGACGAACGCCTCCTGGA GGAAGCTGAACGCCTCCTGGAGGAAGTTGAA CGCTGACTGCAGGCAAGCTTGGC	LCB1

Methods S1. Protocols for vector assembly, transient expression in human cell culture, and analysis by flow cytometry, Related to STAR Methods

Version 1

Oct 12, 2021

Protocol 1A: Spike Display vector assembly

Protocol 1B: Transient expression of Spike Display in human cell culture

Protocol 1C: HEK292T immunostaining and flow cytometry

Protocol 1A: Spike Display vector assembly

All cloning vectors and parts used for Spike Display assemblies have been deposited on Addgene. See the table below for plasmid reference numbers. Addgene plasmids will be shipped as transformed bacteria in stab culture format. Upon receipt, stabs should be kept at 4 °C. Cells from the stab should be streaked out for single colonies on LB Agar plates with the appropriate antibiotic. Using a single colony, inoculate an overnight LB culture and perform plasmid DNA purifications. We recommend sequence verifying all plasmids upon receipt.

Addgene ID	Plasmid name
172721	Spike Display_Part-1 DO
172722	Spike Display_Part-2 DO
172723	Spike Display_Part-3 DO
172724	Spike Display_Part-4 DO
172725	Spike Display_Part-5 DO
172726	Spike Display_Part 1-5 DO
172727	Spike Display_Part 1a Spacer
172728	Spike Display_Part 1b Spacer
172729	Spike Display_Part-1 Spacer
172730	Spike Display_Part-2 Spacer
172731	Spike Display_Part-3 Spacer
172732	Spike Display_Part-4 Spacer
172733	Spike Display_Part-5 Spacer
172734	Spike Display_6P-D614G
172735	Spike Display_B.1.1.7
172736	Spike Display_B.1.351
172737	Spike Display_B.1.1.248
172738	Spike Display_B.1.427/B.1.429

The Spike Display platform features a modular Golden Gate cloning framework for the assembly of spike protein variants. The entirety of the spike protein coding region is divided into 5 parts with junctions strategically positioned at amino acids with low mutational frequencies, according to the GISAID (see below).

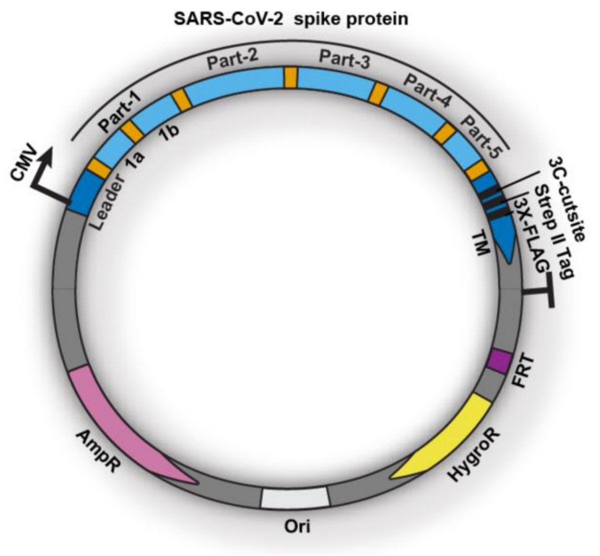

For each of the 5 parts, an entry vector was constructed by cloning in a superfolder GFP (sfGFP) bacterial expression cassette with flanking AarI cut sites and unique 4 nt overhangs matching the wild-type SARS-CoV-2 sequence of each part junction, using PCR and Hi-Fi DNA assembly (see below). A part 1-5 entry vector was made with the entire SARS-CoV-2 coding sequence replaced with the sfGFP cassette to enable multi-part assemblies of complex spike variants or entirely different spike proteins.

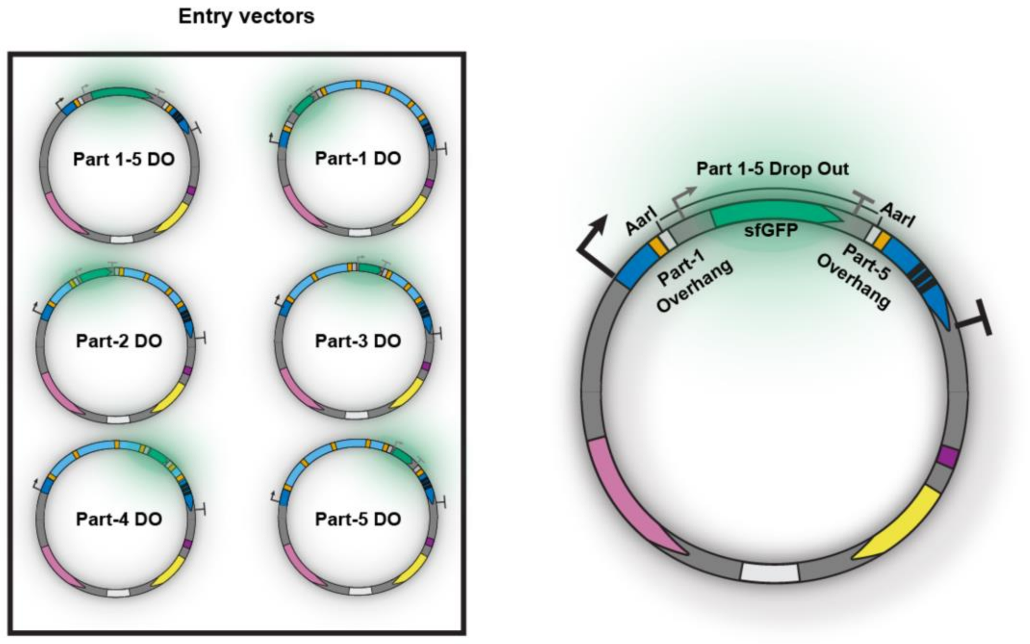

When assembling a new Spike Display vector with the desired mutations, first identify the amino acid (a.a.) position in the spike protein coding sequence. Use the table below to determine which

part your desired mutation(s) fall into. For example, the nonsynonymous mutation N501Y (N->Y mutation at position 501 in the SARS-CoV-2 spike protein coding sequence) is in Part-2.

Part	5' Overhang	3' Overhang	3' Overhang junction (a.a.)	Coding sequence positions (a.a.)
1a	TGAC	GCAA	136-137	1-135
1b	GCAA	TGTG	266-267	138-265
1	TGAC	TGTG	266-267	1-265
2	TGTG	GCCA	522-523	268-521
3	GCCA	GATC	787-788	524-786
4	GATC	CAGA	1054-1055	789-1053
5	CAGA	GCAG	1207-1208	1054-1206

Next, dsDNA gene blocks (synthetic oligos) need to be designed an ordered for each modified part. For these gene blocks to be compatible with the Golden Gate cloning framework used here, 5’ and 3’ overhangs containing AarI endonuclease recognition sequences need to be included (see below). During the Golden Gate assemblies AarI will remove its own recognition sequence and expose 4 nt overhangs on the 5’ and 3’ ends of each Part, which are complementary to the flanking Part or plasmid backbone overhangs.

Gene block designs

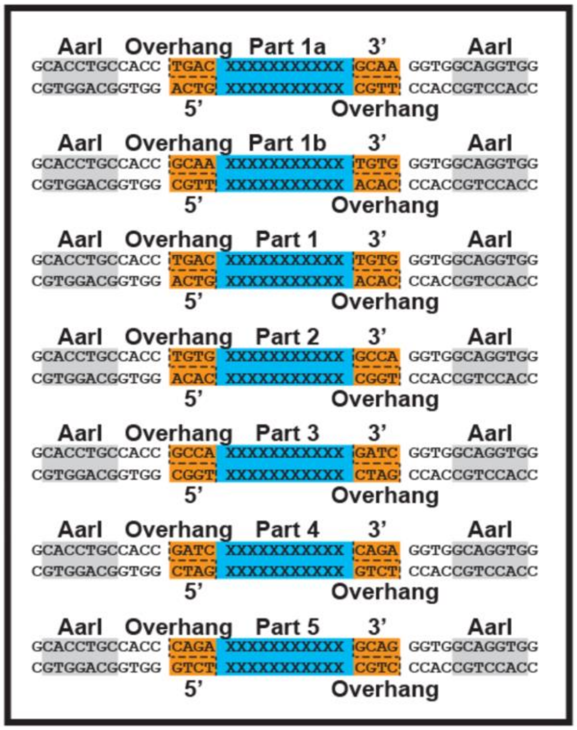

Complete Part sequences for the SARS-CoV-2 spike protein (D614G, ^{682}GSAS^{685}, F817P, A892P, A899P, A942P, K986P, V987P) with the appropriate overhangs are included in the table below. These sequences can be used as templates for ordering custom Parts (dsDNA oligos) with the desired mutations.

<u>Design tips</u>:

1. Consider codon optimization when creating nonsynonymous mutations. As this expression system is tailored for human cell cultures, human codon optimization should be used.
2. Ensure that desired substitutions, insertions, and deletions are in the appropriate reading frame.
3. Perform *in silico* assemblies with the designed parts and entry vectors for QC.
4. Remove any of the following internal cut sites generated during the design of gene block Parts: BsaI, BsmBI, AarI
5. If a desired mutation is located at a Part junction (4 nt overhangs), a larger gene fragment can be purchased which spans the junction. For example, a desired sequence change located at the 3' end of Part 3 can be designed by ordering a Part 3-4 gene fragment. Alternatively, traditional cloning strategies such as site-directed mutagenesis or Gibson assembly can be performed for these special cases.

**Part name**	**Gene Block sequence (5'-3')**
Part-1a	GCATCGTCTCATCGGCACCTGCCACCTGACATGTTCGTGTTCCTG<br>GTGCTCCTGCCTCTGGTGAGCAGCCAGTGCGTGAACCTGACCAC<br>CCGAACCCAGCTCCCACCAGCCTACACCAACAGCTTTACACGGG<br>GCGTGTACTACCCTGACAAGGTGTTCAGATCTAGCGTCCTGCAC<br>AGCACTCAGGACCTCTTCCTGCCGTTCTTCAGCAACGTGACATGG<br>TTCCACGCCATCCACGTGAGCGGCACAAACGGAACCAAGCGGTT<br>TGATAACCCCGTCCTGCCATTCAATGATGGAGTTTACTTCGCCAG<br>TACCGAGAAGAGTAACATCATCCGGGGCTGGATCTTCGGCACCA<br>CCCTGGATAGCAAAACACAGAGCCTCCTGATCGTGAACAATGCC<br>ACGAACGTCGTGATCAAGGTGTGCGAGTTCCAGTTTTGCAAGGT<br>GGCAGGTGGACCTGAGACGGCAT
Part-1b	GCATCGTCTCATCGGCACCTGCCACCGCAATGATCCTTTCCTGGG<br>TGTGTACTACCACAAGAACAACAAGAGCTGGATGGAAAGCGAG<br>TTCAGAGTCTACAGCAGCGCCAACAACTGCACATTCGAGTACGT<br>CTCTCAGCCTTTTCTGATGGACCTTGAGGGGAAACAAGGCAACT<br>TCAAGAACCTGAGAGAATTCGTGTTCAAGAACATCGACGGCTAC<br>TTCAAAATCTACTCCAAGCACACACCCATCAACCTGGTCCGGGA<br>CCTCCCTCAGGGCTTCAGCGCCCTGGAACCCCTGGTCGACCTGCC<br>CATAGGCATCAACATAACGCGGTTCCAAACCCTGCTGGCCCTGC<br>ATAGATCCTACCTGACTCCTGGCGACAGCAGCAGCGGATGGACC<br>GCCGGAGCTGCAGCCTACTATGTGGGTGGCAGGTGGACCTGAGA<br>CGGCAT
Part-1	GCATCGTCTCATCGGCACCTGCCACCTGACATGTTCGTGTTCCTG<br>GTGCTCCTGCCTCTGGTGAGCAGCCAGTGCGTGAACCTGACCAC<br>CCGAACCCAGCTCCCACCAGCCTACACCAACAGCTTTACACGGG<br>GCGTGTACTACCCTGACAAGGTGTTCAGATCTAGCGTCCTGCAC

	AGCACTCAGGACCTCTTCCTGCCGTTCTTCAGCAACGTGACATGG<br>TTCCACGCCATCCACGTGAGCGGCACAAACGGAACCAAGCGGTT<br>TGATAACCCCGTCCTGCCATTCAATGATGGAGTTTACTTCGCCAG<br>TACCGAGAAGAGTAACATCATCCGGGGCTGGATCTTCGGCACCA<br>CCCTGGATAGCAAAACACAGAGCCTCCTGATCGTGAACAATGCC<br>ACGAACGTCGTGATCAAGGTGTGCGAGTTCCAGTTTTGCAATGA<br>TCCTTTCCTGGGTGTGTACTACCACAAGAACAACAAGAGCTGGA<br>TGGAAAGCGAGTTCAGAGTCTACAGCAGCGCCAACAACTGCACA<br>TTCGAGTACGTCTCTCAGCCTTTTCTGATGGACCTTGAGGGGAAA<br>CAAGGCAACTTCAAGAACCTGAGAGAATTCGTGTTCAAGAACAT<br>CGACGGCTACTTCAAAATCTACTCCAAGCACACACCCATCAACC<br>TGGTCCGGGACCTCCCTCAGGGCTTCAGCGCCCTGGAACCCCTG<br>GTCGACCTGCCCATAGGCATCAACATAACGCGGTTCCAAACCCT<br>GCTGGCCCTGCATAGATCCTACCTGACTCCTGGCGACAGCAGCA<br>GCGGATGGACCGCCGGAGCTGCAGCCTACTATGTGGGTGGCAGG<br>TGGACCTGAGACGGCAT
Part-2	GCATCGTCTCATCGGCACCTGCCACCTGTGGGCTACCTGCAACCT<br>AGAACCTTCCTGCTGAAGTACAACGAGAACGGCACAATCACAGA<br>CGCCGTCGACTGCGCCCTGGACCCTCTCTCTGAGACAAAGTGCA<br>CCCTGAAGTCCTTCACCGTGGAAAAGGGCATCTACCAGACCAGC<br>AACTTCCGGGTGCAGCCTACAGAGAGCATCGTGCGATTTCCAAA<br>CATTACCAACCTCTGCCCCTTCGGCGAGGTGTTTAACGCCACAA<br>GATTTGCCTCCGTTTACGCCTGGAATAGAAAGAGAATCAGCAAT<br>TGTGTGGCCGACTACTCCGTGCTGTATAACAGCGCCTCTTTCAGC<br>ACCTTCAAGTGCTACGGCGTTTCCCCAACAAAGCTGAATGACCT<br>GTGCTTCACCAACGTGTACGCCGACTCCTTCGTAATTAGAGGCG<br>ATGAGGTGCGGCAGATCGCACCAGGCCAGACCGGTAAGATCGCT<br>GACTACAACTATAAGCTGCCTGATGATTTTACAGGCTGCGTGAT<br>CGCCTGGAACTCTAACAACCTGGATAGCAAGGTGGGCGGCAACT<br>ACAACTACCTGTACCGGCTGTTTCGCAAGTCTAACCTGAAACCTT<br>TCGAGAGAGACATCTCCACAGAGATCTACCAGGCCGGTTCTACA<br>CCTTGTAACGGGGTGGAAGGCTTCAACTGTTACTTCCCTCTGCAA<br>AGCTACGGCTTCCAGCCTACCAATGGAGTCGGCTACCAGCCATA<br>CCGGGTGGTCGTGCTGTCCTTCGAGTTACTCCACGCCCCCGCCAG<br>GTGGCAGGTGGACCTGAGACGGCAT
Part-3	GCATCGTCTCATCGGCACCTGCCACCGCCACCGTCTGCGGTCCTA<br>AGAAGTCCACCAATCTGGTTAAGAACAAATGCGTGAACTTCAAC<br>TTCAACGGCCTGACCGGGACCGGCGTGCTGACCGAAAGCAACAA<br>AAAGTTCCTCCCCTTCCAGCAGTTCGGCCGTGATATCGCTGACAC<br>CACAGATGCCGTCAGAGATCCACAGACCCTGGAAATCCTGGATA<br>TTACACCCTGCTCCTTCGGAGGAGTTTCTGTGATCACCCCCGGGA<br>CCAATACCAGCAACCAGGTGGCTGTGCTGTACCAAGGTGTTAAC<br>TGCACCGAGGTTCCTGTGGCCATCCACGCCGATCAGCTGACACC<br>TACTTGGAGAGTGTACTCCACTGGCTCCAATGTGTTCCAGACCA<br>GGGCCGGATGTCTGATCGGCGCCGAGCACGTGAATAACAGTTAC<br>GAGTGCGACATCCCTATCGGCGCCGGCATCTGTGCCAGCTACCA<br>GACCCAGACAAACAGCCCTGGGTCTGCTTCCTCTGTAGCTAGCC<br>AGAGCATCATCGCCTACACCATGAGCCTGGGCGCAGAGAACAGC<br>GTGGCCTATTCCAACAACTCTATCGCCATTCCCACCAACTTTACA<br>ATTAGCGTCACAACAGAGATCCTGCCCGTGAGCATGACCAAGAC<br>CAGCGTGGACTGTACAATGTACATCTGTGGCGACAGCACTGAAT

	GCAGCAACCTGCTGCTGCAATACGGCTCCTTTTGCACCCAACTG<br>AACCGGGCGCTGACCGGAATCGCCGTGGAACAGGACAAAAATA<br>CCCAGGAGGTGTTCGCCCAAGTGAAGCAGATCGGTGGCAGGTGG<br>ACCTGAGACGGCAT
Part-4	GCATCGTCTCATCGGCACCTGCCACCGATCTACAAGACCCCACC<br>TATCAAGGACTTCGGCGGCTTTAACTTTAGCCAGATTCTCCCTGA<br>TCCTTCTAAGCCTAGCAAGCGGAGCCCTATCGAGGATCTGCTGTT<br>CAACAAGGTCACCCTGGCCGATGCCGGCTTTATCAAACAGTATG<br>GCGATTGCCTGGGCGACATAGCCGCCAGAGATCTGATCTGCGCC<br>CAGAAATTCAACGGCCTGACAGTTCTCCCACCTCTGCTGACCGA<br>CGAGATGATCGCTCAGTACACCTCTGCCCTGCTGGCTGGCACCA<br>TCACATCTGGGTGGACATTTGGCGCCGGCCCCGCCCTGCAGATC<br>CCCTTTCCCATGCAGATGGCCTATAGATTCAACGGAATCGGCGT<br>GACCCAGAACGTGCTGTATGAAAACCAGAAGCTGATCGCTAACC<br>AGTTCAATTCTGCCATCGGCAAGATCCAGGACTCCCTCTCCTCTA<br>CCCCCAGCGCCCTGGGCAAACTGCAGGACGTGGTGAATCAGAAC<br>GCCCAAGCCCTGAACACCCTGGTGAAGCAGCTCAGCAGCAATTT<br>TGGCGCCATCAGCTCTGTGCTGAACGATATCCTGTCTAGACTGG<br>ACCCTCCAGAAGCCGAAGTCCAGATCGATAGACTGATCACAGGC<br>AGACTGCAGTCCCTGCAAACCTACGTGACCCAACAGCTGATCAG<br>GGCCGCTGAAATAAGAGCCAGCGCCAATCTCGCCGCTACCAAGA<br>TGTCCGAGTGTGTGCTGGGACAGTCTAAACGCGTTGACTTCTGC<br>GGCAAAGGCTATCACCTGATGAGCTTCCCCCAGAGGTGGCAGGT<br>GGACCTGAGACGGCAT
Part-5	GCATCGTCTCATCGGCACCTGCCACCCAGAGCGCGCCGCACGGC<br>GTGGTGTTCCTGCATGTGACATACGTGCCTGCCCAAGAGAAGAA<br>TTTCACAACCGCCCCTGCCATCTGCCACGACGGCAAGGCCCACT<br>TCCCAAGAGAGGGCGTTTTCGTTTCCAATGGCACACACTGGTTC<br>GTGACACAAAGAAACTTCTACGAACCCCAGATTATCACCACCGA<br>CAACACCTTCGTGAGTGGCAATTGTGACGTGGTCATCGGAATCG<br>TGAACAACACAGTGTACGACCCTCTGCAACCTGAGCTGGACTCT<br>TTTAAGGAAGAGCTGGACAAGTACTTTAAAAACCACACCAGCCC<br>CGATGTGGACCTGGGCGACATCAGTGGCATTAACGCCAGCGTGG<br>TGAACATCCAAAAGGAAATCGACAGACTGAACGAGGTGGCCAA<br>GAACCTGAACGAGTCCCTGATCGACCTGCAGGAGCTCGGCAAAT<br>ACGAGCAGGGTGGCAGGTGGACCTGAGACGGCAT

Sequences highlighted in **red** contain BsmBI and AarI recognition sequences as well as the appropriate 5’ and 3’ 4 nt overhangs needed for proper Golden Gate assembly. Simply replace/alter the spike coding sequences in black when designing and ordering gene blocks.

For the assembly of a new Part, the cognate Part-DO vector is required. For example, the assembly of a Part-2 gene block containing a N501Y mutation will require the Part 2 DO vector. These Part + DO vector assemblies should be conducted using the following Golden Gate reaction conditions in PCR strip tubes:

Golden Gate assembly mix (single part):

- 0.25 µL of T7 DNA Ligase (NEB M0318S)
- 0.25 µL of AarI (Thermo Fisher ER1582)
- 1 µL AarI Oligo (1:5 dilution of stock in NFW) (Thermo Fisher)
- 1 µL T4 DNA Ligase Buffer (NEB B0202A)
- 1 µL of the modified Part (10 ng for gene block, 20 ng if in YTK001 plasmid)
- 1 µL of Part DO plasmid (30 ng)
- 5.5 µL of NFW (nuclease free water)

10 µL total reaction volume

Thermocycling (single part):

- 25 cycles
  - 37 °C for 1 minute (digestion)
  - 16 °C for 2 minutes (ligation)
- 1 cycle
  - 37 °C for 30 minutes (final digestion)
  - 80 °C for 20 minutes (heat inactivation)
- 4 °C hold

The Golden Gate cloning framework used for Spike Display allows for the assembly of complex spike variants, containing multiple mutations in different Parts. For example, the alpha variant of concern (VOC), also known as B.1.1.7 contains mutations across all five Parts of the spike coding sequence. To assemble this variant, the Part 1-5 DO vector can be used with customized gene blocks for each of the 5 Parts (see below). These 5 Parts can then be assembled into the Part 1-5 DO vector in a single Golden Gate reaction.

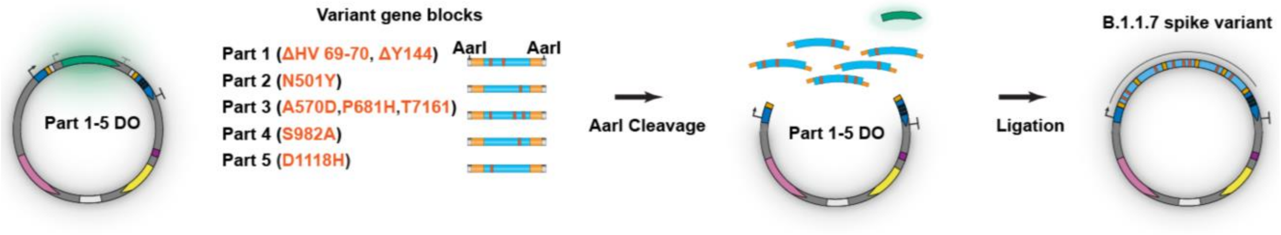

Some spike variants may contain mutations in some Parts but not others. For these assemblies, Part vectors are also available that encode the 6P-D614G spike coding sequences assembled into the YTK001 entry vector (see below). These plasmids can be directly used in the multipart Golden Gate assemblies as they also contain AarI cut sites and the same 4 nt overhangs.

Part vectors

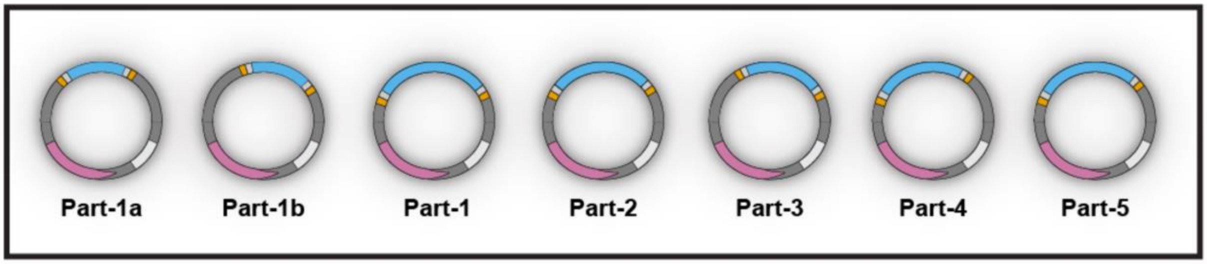

Golden Gate assembly mix (multi-part):

- 0.25 µL of T7 DNA Ligase (NEB M0318S)
- 0.25 µL of AarI (Thermo Fisher ER1582)
- 1 µL AarI Oligo (1:5 dilution of stock in NFW) (Thermo Fisher)
- 1 µL T4 DNA Ligase Buffer (NEB B0202A)
- 1 µL of each modified Part (10 ng for gene block, 20 ng if in YTK001 plasmid)
- 1 µL of Part DO plasmid (30 ng)
- 1.5 µL of NFW (nuclease free water)

10 µL total reaction volume

Thermocycling (multi-part):

- 50 cycles
    - 37 °C for 2 minutes (digestion)
    - 16 °C for 4 minutes (ligation)
- 1 cycle
    - 37 °C for 60 minutes (final digestion)
    - 80 °C for 20 minutes (heat inactivation)
- 4 °C hold

Transformations:

- Thaw out 50 µL of Zymo DH10β Mix & Go Competent Cells (prepared using Zymo T3019) for each plasmid transformation, on ice.
- Transfer 4 µL from each unique assembly to the respective 1.5 mL Eppendorf tube containing 50 µL of competent cells. Flick tubes gently to mix contents and briefly spin down.
- Incubate mixtures on ice for 10 minutes on ice or a 4 °C block.
- Add 150 µL of superior broth (AthenaES 0105) to each tube and perform outgrowth for 1 hour at 37 °C while shaking (tubes on their side for optimal growth/recovery).

- Outgrown cells were plated dropwise on Nunc OmniTrays (5 µL per spot) (Thermo Fisher 140156), containing LB-agar + carbenicillin at 100 µg mL-1. Each plate can fit 96, 5 µL drops. Plates were kept at room temperature until the drops dried and were then transferred to a 37 °C incubator for growth overnight (12-16 hours).
- Colonies should be screened with blue light to discriminate between fluorescent (green/gfp) and non-fluorescent colonies. Colonies still containing the sfGFP-DO and not the desired Part(s) will retain green fluorescence and should not be picked.
- Colonies can be picked into 1 mL of SB media with Carbenicillin (100 µg/mL) in Axygen deep well grow blocks and grown overnight at 37 °C while shaking.
- Once grown, liquid cultures should be pelleted at 3000 g for 10 minutes and miniprepped using the Promega Wizard SV 96 Plasmid DNA Purification Kit (Promega A2250).
- All plasmid assemblies should sequence verified.

Troubleshooting:

- No colonies on the plate:
    - QC competent cells by transforming a Part or Part-DO vector alone. High transformation efficiency commercial competent cells can also be used.
    - Ensure that the correct antibiotics are used for the LB-Agar plate. Part plasmids are Chloramphenicol resistant. Part-DO plasmids are Ampicillin/Carbenicillin resistant.
    - Plate more of the transformed/outgrown cells to compensate for inefficient assemblies.
    - Ensure that the T7 DNA Ligase and T4 DNA Ligase buffer are fresh and have not undergone excessive freeze thaw cycles.
- All colonies are green:
    - Check the concentrations of each Part and Part-DO vector to ensure that correct input concentrations are used for the Golden Gate assembly. These reactions are sensitive to the molar ratio of each DNA component.
    - QC the restriction endonuclease being used with the Part and Part-DO vectors. Run cleaved products on a gel to ensure the separation of insert and backbone. If this reaction fails, either the endonuclease is inactive or the plasmids preparations have impurities, inhibiting the digestion reactions.
    - If using AarI, ensure that the AarI oligo is being added to the reaction. Unlike other endonucleases AarI requires this trigger oligo for efficient cleavage activity.
- Incomplete multi-part assemblies:
    - On occasion, promiscuous cleavage/ligation events will occur during multi-part assemblies causing the dropout of individual Parts. For inefficient multi-part assemblies screen 4 or more colonies and sequences across the entire spike coding sequence.

Protocol 1B: Transient expression of Spike Display in human cell culture

Culturing and seeding cells:

- HEK293T cells should be cultured in:
  - DMEM (Gibco 11995065) containing phenol red, 4 mM L-glutamine, 110 mg L-1 sodium pyruvate, 4.5 g L-1 D-glucose
  - 10% FBS (Gibco 26140079)
  - 2% Pen/Strep (Thermo Fisher 15070063)
- Cells lines should be tested for mycoplasma contamination before use. The Mycoplasma Detection Kit (SouthernBiotech 13100-01) is highly recommended.
- Cells should be maintained in a humidified atmosphere of 5% CO_2 and 37 °C.
- Passage cells every 2-3 days into 10 cm polystyrene coated plates (VWR 10062-880) upon reaching high density.
- Approximately 24 hours before transfection, cells should be seeded into 6-well or 12-well polystyrene coated plates (VWR 10861-696, 10861-698) at a density of 0.3 x 10^6 cells mL^{-1} or 0.1 x 10^6 cells mL^{-1}, respectively.

Transfection:

- Upon reaching 60-80% confluence, cells are ready for transfection.
- For each Spike Display expression vector, we recommend transfecting 2-3 biological replicates (2-3 separate wells of seeded cells).
- For each sample/plasmid set up a sterile Eppendorf tube with Opti-MEM (Gibco 51985091).
  - 100 μL/tube for 12-well plates
  - 200 μL/tube for 6-well plates
- Add endotoxin free plasmid DNA to each tube.
  - 1 μg/tube for 12-well plates
  - 2 μg/tube for 6-well plates
- In a separate Eppendorf tube create a master mix containing:
  - 100 μL or 200 μL of Opti-MEM per sample, for 12- and 6-well plates respectively
  - 3 μL of Lipofectamine 3000 (Thermo Fisher L3000015) per ug of plasmid DNA
  - Recommendation: include 10% excess of each reagent to compensate for solution loss during pipetting.
- Add the Opti-MEM/Lipofectamine master mix to each tube containing plasmid DNA + Opti-MEM
  - 100 μL/tube for 12-well plates
  - 200 μL/tube for 6-well plates
- Incubate reactions in the hood, for 20 minutes. Shorter or longer incubations are detrimental to transfection efficiencies.
- Add each transfection mix (plasmid DNA + Lipofectamine + Opti-MEM) to the appropriate wells containing seeded cells (60-80% confluence) in a drop-wise fashion.
- Return plate(s) to the 37 °C incubator and wait 48 hrs before collecting cells. This will give the spike proteins sufficient time to be post translationally modified, trafficked, and displayed on the cell surface.

Protocol 1C: HEK292T immunostaining and flow cytometry

Collecting cells:

- HEK293T cells with surface-displayed spike should be collected 48 hours post-transfection
- First, wash once with PBS (volume equivalent to the media in each well).
- Next, resuspend in PBS by gentle pipetting until all the cells have been dislodged from the cell surface and are essentially monodispersed (no clusters). HEK293Ts tend to stick and aggregate.
- Transfer resuspended cells from each well to a sterile Eppendorf tube (1 tube per sample).
- Use 20 uL of the resuspended cells to determine cell density. An automated cell counter (Logos Biosystems L40002) was used for this study.
- Cells should then be spun down at 200 g for 1 minute.
- After decanting the supernatant resuspend the cells with chilled PBS-BSA (1% BSA (Sigma-Aldrich A3294), 1x PBS, 2 mM EDTA pH 7.4) to a density of ~3×10^6 cells mL^{-1}. Lower cell densities can be used if necessary.
- Cells can be kept in PBS-BSA for several hours if chilled (4 °C). However, for best immunostaining and flow cytometry results, assay cells immediately after collection.

Immunostaining cells:

- Flow cytometry assays can be prepared in Axygen Deep well grow blocks (P-2ML-SQ-C-S).
- Each well contains a predetermined concentration of primary antibody or chimeric cell receptor (ACE2-Fc) diluted in PBS-BSA (500 uL) and 50 µL (1.5×10^5) of cells.
- Mixtures should be incubated at room temperature and shaken at 950 rpm for 1 hour. This will provide sufficient time for antibodies or soluble proteins to bind the displayed spike antigen.
- Cells are then pelleted by spinning the plate for 2 minutes at 500 g in a swinging bucket rotor.
- Wash cells by carefully decanting the supernatant and adding 500 µL of PBS-BSA to each well. Spin cells again at 500 g for two minutes and repeat wash.
- 500 µL of a secondary antibody solution (5 µM Alexa Fluor® 488 anti-mouse (SouthernBiotech 1031-30) and 10 µM Alexa Fluor® 647 anti-human (SouthernBiotech 2048-31) in PBS-BSA) was added to each well.
- Incubate the plate in the dark, at 4 ˚C while shaking at 950 g for 25 minutes. Longer incubation times will result in the gradual dissociation of the primary antibodies. For antibodies or soluble cell receptors with weak binding affinities, consider primary labels to improve fluorescent signal while analyzing via flow cytometry.
- Wash the wells again, twice with PBS-BSA, and then resuspended in 300 µL of PBS-BSA before running on the SA3800 Spectral Analyzer (SONY).

Flow cytometry:

HEK293T cells were used to establish forward scatter-area (FSC-A) and side scatter-area (SSC-A) gating (see below). Singlet discrimination was then established with forward scatter-height (FSC-H) vs forward scatter-area (FSC-A) and side scatter-height (SSC-H) vs side scatter-area (SSC-A) gates. A minimum of 10,000 singlet events were acquired for each assayed sample. Variations of these gates and cell counts can be used for each assay if need. However, using a template for consistent gating and data analysis is recommended.

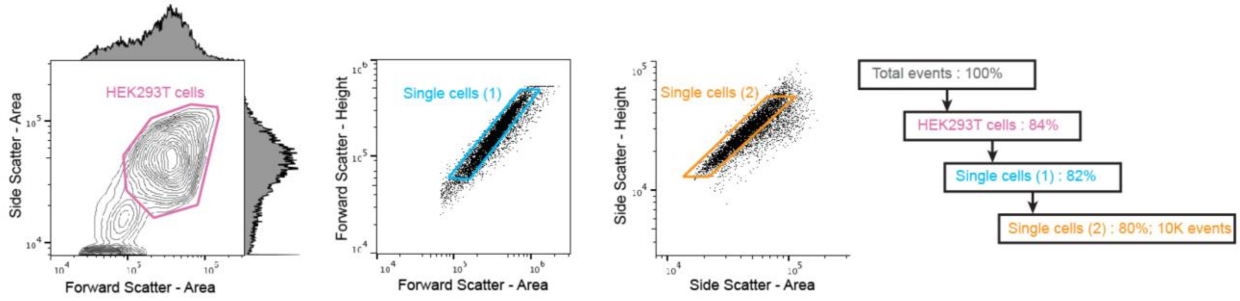

These singlet HEK293T cells are further analyzed in two fluorescent channels, Alexa Fluor 488 (AF-488) and Alexa Fluor 647 (AF-647), using manufacturer-recommended excitation and detection settings.

Spectral unmixing should be applied to all data to reduce the effect of spectral spillover and autofluorescence on downstream calculations. The protocol for spectral unmixing and the creation of fluorescent compensation matrixes will vary depending on the flow cytometer and the software used to analyze the data.

See below for a graphical representation of the antibodies used (left) and the resulting flow cytometry data measuring ACE2 binding of 6P-D614G and B.1.1.7 spike variants (right). Single cells from the gating workflow described above were analyzed using two fluorescent channels, Alexa Fluor-488 and Alexa Fluor-647 for measuring spike expression and antigenicity, respectively.

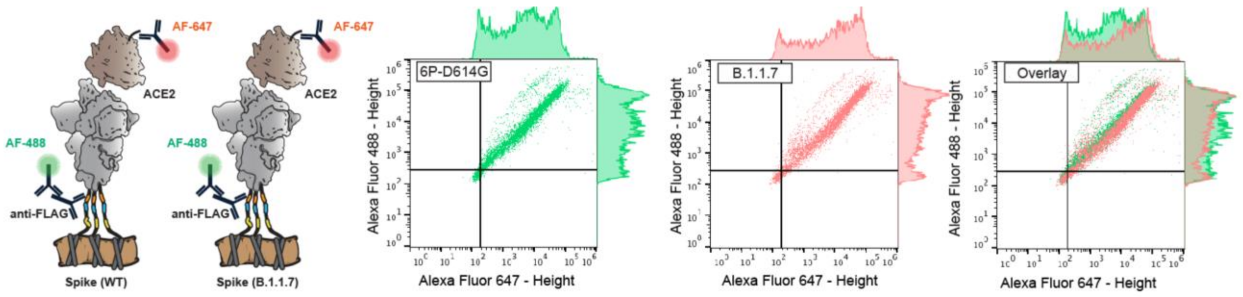

A similar graphical representation is shown for a different antibody (4A8), which interacts with the SARS-CoV-2 spike proteins N-terminal domain (NTD) (see below). Unlike ACE2, 4A8's binding profile (AF-647 signal) differs greatly between the WT (6P-D614G) and B.1.1.7 spike variants.

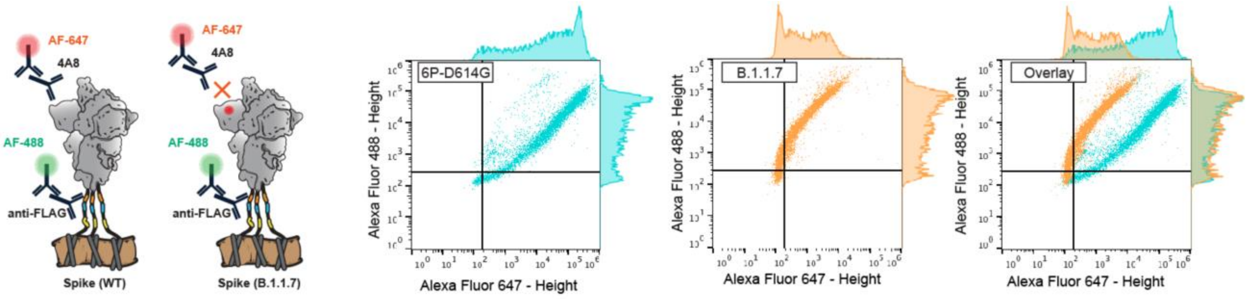

Data analysis:

Median height (H) measurements for the AF-488 and AF-647 channels should be recorded for each sample. Anti-FLAG (AF-488 channel) signal is used to measure spike expression. Spike variant (x) expression relative to WT (6P-D614G) can be calculated using the following equation:

$$Normalized\ expression = \ Log_2(Median{:}\,488\_H_x/Median{:}\,488\_H_{6P\_D614G}$$

Anti-FLAG signal is also used as an internal normalization control to correct for changes in transfection efficiency and spike expression when measuring antibody or ACE2 binding. Normalized binding measurements for spike variants (x) expression relative to WT (6P-D614G) can be calculated using the following equation:

$$Normalized\ binding = \ Log_2\left(\frac{Median{:}\,647\_H_x/Median{:}\,488\_H_x}{Median{:}\,647\_H_{6P\_D614G}/Median{:}\,488\_H_{6P\_D614G}}\right)$$

For antibody titration curves, median height (H) measurements for AF-647 are divided by the respective AF-488 signal for each sample. This provides a normalized binding measurement relative to each sample's expression signal (Anti-FLAG; AF-488). All titrations were conducted using biological triplicate (three separate transfections). All titration points for each curve were then divided by the max value in the set, thus normalizing the curve to a range between 0 and 1 normalized binding units. These values can then fit with a Sigmoidal, 4PL function where X is concentration (least squares fit).

All flow cytometry data for this study was analyzed using FlowJo v9.
